# Characterizing functional connectivity gradients for the hippocampus–amygdala complex in healthy and psychiatric cohorts

**DOI:** 10.1007/s00429-026-03134-4

**Published:** 2026-06-03

**Authors:** Xiang-Shen Liu, Janna N. Vrijsen, Linlin Yan, Koen V. Haak, Rose M. Collard, Philip F. P. van Eijndhoven, Christian F. Beckmann, Guillén Fernández, Indira Tendolkar, Nils Kohn

**Affiliations:** 1 Department of Medical Neuroscience, Donders Institute for Brain, Cognition and Behaviour, Radboud University Medical Centre, Nijmegen, The Netherlands; 2 Department of Psychiatry, Donders Institute for Brain, Cognition and Behaviour, Radboud University Medical Centre, Nijmegen, The Netherlands; 3 Depression Expertise Center, Pro Persona Mental Health Care, Nijmegen, The Netherlands; 4 Department of Cognitive Science and Artificial Intelligence, Tilburg University, Tilburg, The Netherlands; 5 Department of Psychiatry, Radboud University Medical Centre, Nijmegen, The Netherlands; 6 Department of Psychiatry and Psychotherapy, Medical Faculty, LVR-University Hospital Essen, University of Duisburg-Essen, Essen, Germany

**Keywords:** Hippocampus–amygdala complex, Functional connectivity, Connectopic mapping, Serotonin, Dopamine, Psychopathology

## Abstract

The hippocampus and amygdala play essential roles in human cognition and emotion, through their extensive connectivity with other brain regions and close interaction between them. Uncovering the functional organization of the hippocampus–amygdala complex and its relationship with neurotransmitter distribution can enhance our understanding of their biological functionality, and provide a basis for further exploration of the clinical relevance. An emerging functional connectivity analysis method termed connectopic mapping, may offer a novel approach to characterize this functional organization. In this study, we applied connectopic mapping to the hippocampus-amygdala complex, testing its utility with resting-state functional magnetic resonance imaging (fMRI) scans of two independent datasets: one comprising healthy individuals (*N* = 410) and another comprising a psychiatric cohort (*N* = 367). The spatial organization of derived gradient maps was compared to 18 positron emission tomography (PET) or single photon emission computed tomography (SPECT) scan templates for different neurotransmitter systems. Individual gradient–neurotransmitter similarity indices were correlated with mental health outcomes. Our analyses identified six distinct gradient maps in both datasets. The third-order gradients showed stable similarity with 5-HT1A receptor maps across various resting-state scans. Similarities were also observed between gradient maps and the distribution patterns of neurotransmitters within the dopaminergic system. Individual gradient-to-5-HT1A similarity was positively correlated with depressive severity and anxiety sensitivity, highlighting the psychopathological relevance. These findings demonstrate that across the psychiatric continuum, connectopic mapping is a powerful tool for exploring the relationship between functional connectivity organization and neurotransmitter distribution, showing potential as a comprehensive transdiagnostic biomarker.

**Supplementary Information:**

The online version contains supplementary material available at 10.1007/s00429-026-03134-4.

## Introduction

The hippocampus and amygdala, as two adjacent medial temporal lobe (MTL) structures, have consistently been investigated as key brain regions in cognitive affective neuroscience (LaBar and Cabeza 2006; Panksepp et al. 2017). The hippocampus is essential for episodic memory, enabling our memory for daily events built up with spatial, temporal and other contextual information (Barker and Warburton 2011; Moscovitch et al. 2016). The anterior segment of hippocampus is closely adjacent to the amygdala—the central region for emotional responses to environmental stimuli, participating in circuits that process heightened arousal and survival-related emotions (Garavan et al. 2001; Ewbank et al. 2009; Kohn et al. 2011; Hrybouski et al. 2016). Effective functioning of these two anatomically adjacent regions is inseparable from their close interactions with each other (Phelps 2004; Richardson et al. 2004; LaBar and Cabeza 2006), as well as their widespread functional connections with other brain regions. In particular, connections with the medial prefrontal cortex (mPFC), anterior temporal lobe, insula and posterior cortical areas support episodic encoding and retrieval, aversive learning, as well as stress and emotion regulation (Roy et al. 2009; van Marle et al. 2009, 2010; van Kesteren et al. 2010; Poppenk et al. 2013; Kohn et al. 2014; Likhtik and Paz 2015). Maladaptive variations in these neural circuits are commonly observed in mood disorders such as depression (Cullen et al. 2014; Mulders et al. 2015), anxiety disorders (Shin and Liberzon 2010; Brehl et al. 2020) and post-traumatic stress disorder (PTSD; Rauch et al. 2006; Likhtik and Paz 2015), and may also account for certain comorbidity across these.

Notably, studies demonstrated that functional domains within the hippocampus vary along its long, curved posterior-to-anterior axis, with gradients observed in gene expression, place cell field sizes, and behavioral profiles such as emotion-cognition and familiarity-novelty (Poppenk et al. 2013; Strange et al. 2014; Plachti et al. 2019; Quent et al. 2025). Anatomically, dorsoventral (corresponding to anterior-posterior in human) topographical gradients in hippocampal–cortical and subcortical connectivity were revealed in animal studies (Witter 1993; Kishi et al. 2006), which may provide a structural substrate for the functional gradient organization. Using noninvasive imaging techniques such as fMRI, some studies have broadly replicated these topographical gradients in living humans based on resting-state functional connectivity (Vos de Wael et al. 2018; Przeździk et al. 2019; Masouleh et al. 2020; Xie et al. 2024). However, further evidence is still needed to achieve a better understanding of their biological features (e.g., relationships with neurotransmitter modulation and gene expression), whether such topographical organizations generalize across healthy and psychiatric populations, and whether their individual variability may relate to psychiatric phenotypes (Genon et al. 2021; Borne et al. 2023). These investigations will help us build a clear overview of hippocampal function and dysfunction.

Similarly, projections between the amygdala and various cortical regions (e.g., mPFC, inferior temporal cortex), as well as subcortical structures (e.g., the striatum), have also been found to exhibit a topographically organized pattern (Stefanacci and Amaral 2000, 2002; Haber 2003). As a compact, deep-lying nucleus, however, the amygdala’s small size may limit the suitability of investigating this topography in humans using neuroimaging techniques, due to imaging resolution constraints. The hippocampus and amygdala are not only spatially adjacent structures, but both play closely interconnected roles within cognitive and affective functional networks; the amygdala is often regarded as a unit with the hippocampus when examining its functional role or psychiatric aberrance (Rajarethinam et al. 2001; Rutishauser et al. 2006). Following this approach, and considering practical advantages, we would like to integrate the hippocampus and amygdala into a single complex, and capture the functional connectivity organization of this complex, through a data-driven technique connectopic mapping. This method is designed to identify multiple overlapping connectivity patterns, defined as gradients, within a predefined region of interest (ROI), with each gradient representing a distinct topographic mode of connectivity variation within the ROI relative to the rest of the brain (Haak et al. 2018). By decomposing functional connectivity profiles into multiple components, connectopic mapping is particularly suited to understand the functional multiplicity of a given region—the coexistence of multiple overlapping functional organization patterns within a single area, of which the hippocampus and amygdala are typical examples. Thus, in the current study, we applied connectopic mapping to characterize functional connectivity organization for the hippocampus-amygdala complex in both healthy and psychiatric populations.

After obtaining these data-derived topographic gradients, the next necessary step is to better understand their biological features, among which the relationship with neurotransmitter systems represents an important aspect. The hippocampus and amygdala are extensively modulated by multiple neurotransmitter systems that regulate their communication with other regions of functional networks (Vizi and Kiss 1998; Garrido Zinn et al. 2016). Typical examples include the regulation of hippocampal long-term potentiation by *N*-methyl-D-aspartate (NMDA) glutamatergic receptors and beta-noradrenergic receptors (Izquierdo et al. 1993), as well as the role of beta-noradrenergic signaling in the amygdala fear-conditioning pathway (Bush et al. 2010; Krugers et al. 2012). Other neurotransmitter systems, such as serotonin and dopamine, also prominently modulate the fear circuitry, emotional processing and learning (Bocchio et al. 2016; Frick et al. 2022). These essential roles of neurotransmitters in the functional network of the hippocampus–amygdala complex might be captured by topographic gradients. Recent studies have reported spatial correspondences between patterns of resting-state functional connectivity and certain neurotransmitter distributions (Oldehinkel et al. 2022; Saiz-Masvidal et al. 2025). Following this line of work, we also compared spatial topologies of hippocampus-amygdala gradient maps with PET or SPECT scan templates for multiple neurotransmitter systems. The similarity in spatial layouts offers a proxy for estimating the potential association with specific neurotransmitter systems and helps interpret biological meanings of these data-driven gradients (Oldehinkel et al. 2022; Nordin et al. 2025).

Neurotransmitter modulations in the hippocampus-amygdala complex are closely associated with mental health and the development of various psychiatric disorders. For example, altered function of serotonin receptors and transporters in the hippocampus and amygdala is commonly observed in mood disorders (Savitz and Drevets 2013; Albert et al. 2014). Such alterations influence individuals’ coping mechanisms following stress (Puglisi-Allegra and Andolina 2015) and interact with childhood trauma to further shape depressive symptomatology (Bartlett et al. 2023). Variations in connectivity gradients have been shown to sensitively capture individual differences (Liu et al. 2024; Serhan et al. 2025). Here, we characterized the gradient-neurotransmitter spatial correspondence for each participant, and tested whether individual differences in this correspondence could reflect variability in mental health-related factors, with depressive and anxiety levels as indicators of current symptomatology, and childhood trauma as a measure of clinically relevant past experiences.

By applying connectopic mapping to the hippocampus-amygdala complex, we aim to (1) create an overview of its functional connectivity organization by identifying several topographical gradients; (2) examine biological features of these gradients by assessing their relationship with neurotransmitter layouts; and (3) explore if individual differences in the gradient-neurotransmitter relationship are related to mental health factors. To evaluate the generalizability and potential translational relevance of these topographic gradients, we included two independent datasets with different levels of psychopathology: the Healthy Brain Study (HBS) comprising healthy adults (Healthy Brain Study consortium et al. 2021), and ‘Measuring Integrated Novel Dimensions in Neurodevelopmental and Stress-related Mental Disorders’ (MIND-Set) study including a highly comorbid psychiatric cohort (van Eijndhoven et al. 2022).

## Materials and methods

### Participants

In this study, two databases were utilized and analyzed independently. One dataset was from the Healthy Brain Study, jointly conducted by Radboud University, Radboud University Medical Center, and the Max Planck Institute for Psycholinguistics in Nijmegen, the Netherlands. The analysis included all participants from the first data release (*N* = 410; 169 males; mean age = 33.8 ± 2.8 years; see Suppl.Table S1 for characteristic information of the two samples). Participants had no history of psychiatric illness or current diseases affecting the brain, and none were taking brain-targeted medication at the time of participation. The study protocol and more details can be found in Healthy Brain Study Consortium et al. (2021).

Another dataset was from the MIND-Set cohort, collected by the Department of Psychiatry at Radboud University Medical Center and the Donders Institute in Nijmegen, the Netherlands. All participants with available resting-state neuroimaging data were included (*N* = 367; 202 males; age 37.6 ± 14.0 years). In this sample, 286 participants were psychiatric patients (164 males; age 37.6 ± 13.4 years; see Table S1 for *n* per diagnosis), and 81 individuals did not have a current or past psychiatric disorder (37 males; age 37.67 ± 15.80 years). 242 participants were taking one or more medications during their participation. In Table S2, we list the frequencies of different types of nervous system medication use in the sample. More details on the sample were introduced in van Eijndhoven et al. (2022).

### fMRI data acquisition and preprocessing

The HBS study includes three resting-state scans, conducted within one year, each separated by a four-month interval. T2*-weighted resting-state BOLD data were acquired using a multiband-accelerated gradient echo EPI sequence (66 slices; TR = 1000 ms; TE = 34 ms; flip angle = 60°; voxel size = 2.0 × 2.0 × 2.0 mm; FOV = 210 mm), with the duration of 10 min. Preprocessing steps comprised motion correction, distortion correction with field maps, spatial smoothing with a 4 mm FWHM Gaussian kernel, and non-linear registration to MNI152 space. FSL FIX’s ICA and Gradient Distortion Correction were used for further denoising and distortion correction (Healthy Brain Study consortium et al. 2021) (https://osf.io/jzwrg/).

In the MIND-Set study, three resting-state scans were conducted as well. Resting-state scans 1 and 2 each lasted 8.5 min and were separated by a neutral movie clip. Following resting-state scan 2, an aversive movie clip was shown to induce acute stress. Resting-state scan 3, lasting 12.6 min, was then acquired. All resting-state scans used a multi-band 6 protocol with an interleaved slice acquisition sequence to capture T2*-weighted EPI BOLD-fMRI images (66 slices; TR = 1000 ms; TE = 34 ms; flip angle = 60°; voxel size = 2.0 × 2.0 × 2.0 mm; FOV = 210 mm). Preprocessing was performed using FSL version 5.0.11 (FMRIB, Oxford, UK), including brain extraction, motion correction, bias field correction, high-pass temporal filtering (100s cut-off), spatial smoothing with a 4 mm FWHM Gaussian kernel, boundary-based registration to T1-weighted images, and nonlinear registration to standard space (MNI152). Motion-related artifacts were removed using ICA-based Automatic Removal of Motion Artifacts (ICA-AROMA). Further details on fMRI acquisition and preprocessing can be found in previous studies (van Oort et al. 2020; van Eijndhoven et al. 2022).

### Characterizing hippocampus-amygdala connectivity gradients

For both datasets, we applied ConGrads (Haak et al. 2018) to the preprocessed resting-state data (three scans per dataset), using the left and right hippocampus-amygdala complexes as ROIs. The ROI masks were derived from the Harvard-Oxford atlas with a threshold of 20% probability. To better visualize gradient changes along the hippocampal long axis, the functional image data and masks were rotated around the X-axis in MNI152 space by an angle of 37°. With resting-state fMRI data as input, ConGrads identifies several topographic organization modes of functional connectivity variation within the ROI in relation to the rest of the brain. The detailed pipeline of ConGrads has been described elsewhere (Marquand et al. 2017; Haak et al. 2018). In brief, a similarity matrix is first constructed by computing time-series correlations between each voxel within the ROI mask and all other grey-matter voxels. A manifold learning algorithm (Laplacian eigenmaps) is then applied to this similarity matrix to derive a set of topographic gradient maps. Each gradient map captures a distinct mode of spatial variation within the ROI, reflecting gradual changes in its functional connectivity with the rest of the grey matter. These gradient maps are mutually orthogonal and exhibit a hierarchical ordering: the lowest and most dominant mode (the zeroth-order gradient) accounts for the largest proportion of variance in the functional connectivity organization, with the explained variance decreasing for higher-order gradients (Margulies et al. 2016; Nordin et al. 2025).

For each resting-state scan of the two datasets, we derived group-average gradient maps by combining functional image data from all available participants as inputs, separately for the left and right hippocampus-amygdala complexes. To enable statistical comparisons across participants, the order of individual gradient maps was adjusted by swapping gradients based on their spatial correlation with group-average maps (i.e., swapping within the participant when the other gradient map showed a higher correlation coefficient with the group average than the original one). After swapping, as a quality control measure, individual maps were excluded if their spatial correlation with the group-average map was below 0.50 (Oldehinkel et al. 2022). We monitored the proportion of participants retained at this threshold and determined the number of gradients based on the point at which a sharp decline in this proportion was observed.

To characterize the spatial organization of these gradient maps, and validate their statistical representation, a trend surface model (TSM) was applied to both group-average and individual gradient maps (Haak et al. 2018). TSM represents the value of a property (Pi) at each spatial location as a regression function of its coordinates (Xi, Yi, Zi), yielding a set of regression coefficients that act as low-dimensional descriptors of the spatial mode (Gelfand et al. 2010). The degree of similarity in spatial organization can therefore be inferred from similarities in the structure of TSM coefficients. As an alternative to using voxel-wise gradient values, TSM helps to reduce the potential spatial autocorrelation effects, as it does not rely on individual voxel values but instead captures spatial variations across the space, from coarser-to-finer levels (Haak and Beckmann 2020; Nordin et al. 2025). Following previous work on hippocampal gradients (Przeździk et al. 2019), a trend surface regression model with nine coefficients was chosen, consisting of three parameters for each of the X, Y, and Z axes.

### Mapping gradients with neurotransmitters

To better understand the biological feature of these data-driven gradient maps of functional connectivity organization, we examined their relationships with multiple neurotransmitter systems. We utilized PET or SPECT scans for various neurotransmitters from the publicly available JuSpace toolbox (https://github.com/juryxy/JuSpace; version 1.5, downloaded on 6.6.2023). Following prior research exploring striatal connectivity gradients and neurotransmitters (Oldehinkel et al. 2022), a total of 18 neurotransmitter templates were included (see Tables S3 and S4 for a full list and detailed information).

The same trend surface regression model, with nine coefficients, was applied to these PET/SPECT scans, with the left and right hippocampus-amygdala complexes defined as ROIs. Correlation coefficients were then calculated between the TSM coefficients obtained from these neurotransmitter maps and the TSM coefficients characterizing the group average hippocampus-amygdala gradient maps. To standardize comparisons, the absolute correlation coefficients were transformed using Fisher’s r-to-z transformation. To examine the statistical significance of these gradient-neurotransmitter correlations, permutation testing was performed (*N* = 10,000, *p* < 0.05, FDR corrected). A null distribution was generated by permuting the PET/SPECT TSM coefficients (separately for each coefficient) and calculating correlations between gradient TSM coefficients and permuted PET/SPECT TSM coefficients (Fisher r-to-z transformed, absolute values). The observed correlations were then compared to this null distribution.

To provide a comprehensive assessment of the spatial correspondence between gradients and neurotransmitter layouts, in addition to the permutation testing described above, we also estimated TSM-based correlation coefficients between the two using parametric testing (Pearson’s r, FDR correction, *p* < 0.05), as well as voxel-wise correlation coefficients (Oldehinkel et al. 2022). Moreover, we examined whether using different TSM model orders (e.g., TSM with 6 or 12 coefficients) affected these spatial correspondences.

In addition, to assess the potential influence of head motion, participants in HBS were divided into high- and low-motion subgroups based on a median split of their mean relative displacement, and gradient–neurotransmitter spatial similarity was calculated separately for each subgroup. Similarly, within the MIND-Set sample, gradient–neurotransmitter spatial similarity was also estimated after separating psychiatric patients and healthy controls, as well as participants with and without medication use.

### Linking individual gradient-neurotransmitter similarity with behavioral outcomes

Neurotransmitter modulations in the hippocampus-amygdala complex are relevant for psychopathology. Therefore, we also examined whether individual differences in gradient-to-neurotransmitter similarity could be associated with variations in behavioral outcomes for mental health. For neurotransmitters that showed similarity in spatial layouts with the group-average connectivity gradients, we calculated the correlation coefficients between the TSM coefficients of individual gradient maps per participant and TSM coefficients of PET/SPECT data in the JuSpace toolbox. Subsequently, Spearman correlation analysis was conducted between the Fisher r-to-z transformed absolute correlation coefficients and behavioral outcomes (*p* < 0.05, FDR corrected).

For the behavioral outcomes, we included depression and anxiety symptom levels as psychiatric measures, and childhood trauma as a pathogenic environmental factor. In both datasets, the severity of depressive symptoms was measured using the Inventory of Depressive Symptomatology-Self Report (IDS-SR; Rush et al. 2000), and anxiety sensitivity was assessed with the Anxiety Sensitivity Index (ASI; Rodriguez et al. 2004). In the HBS dataset, childhood trauma was measured using the Childhood Trauma Questionnaire-Short Form (CTQ-SF; Hagborg et al. 2022). Both the total score and subscale scores (emotional neglect, physical neglect, emotional abuse, physical abuse) were included. In MIND-Set, childhood trauma frequency and diversity was measured using the questionnaire from the Netherlands Mental Health Survey and Incidence Study (NEMESIS; Graaf et al. 2010). Both the overall index and subscales (representing the frequency of occurrence of emotional neglect, psychological abuse, and physical abuse before the age of 16 years) were included in the analysis. Additionally, for this psychiatric cohort, we accounted for comorbidity levels by summing the number of diagnosed psychiatric disorder clusters (i.e. Mood Disorder, Anxiety Disorder including PTSD and Obsessive Compulsive Disorder, Attention-Deficit/Hyperactivity Disorder, Autism Spectrum Disorder, and Addiction) for each participant.

We also repeated the above correlation analysis using residuals of the behavioral variables after regressing out age, sex, head motion, and medication use, to control for potential confounding factors.

## Results

### The hippocampus-amygdala functional connectivity gradient maps

**Fig. 1:**
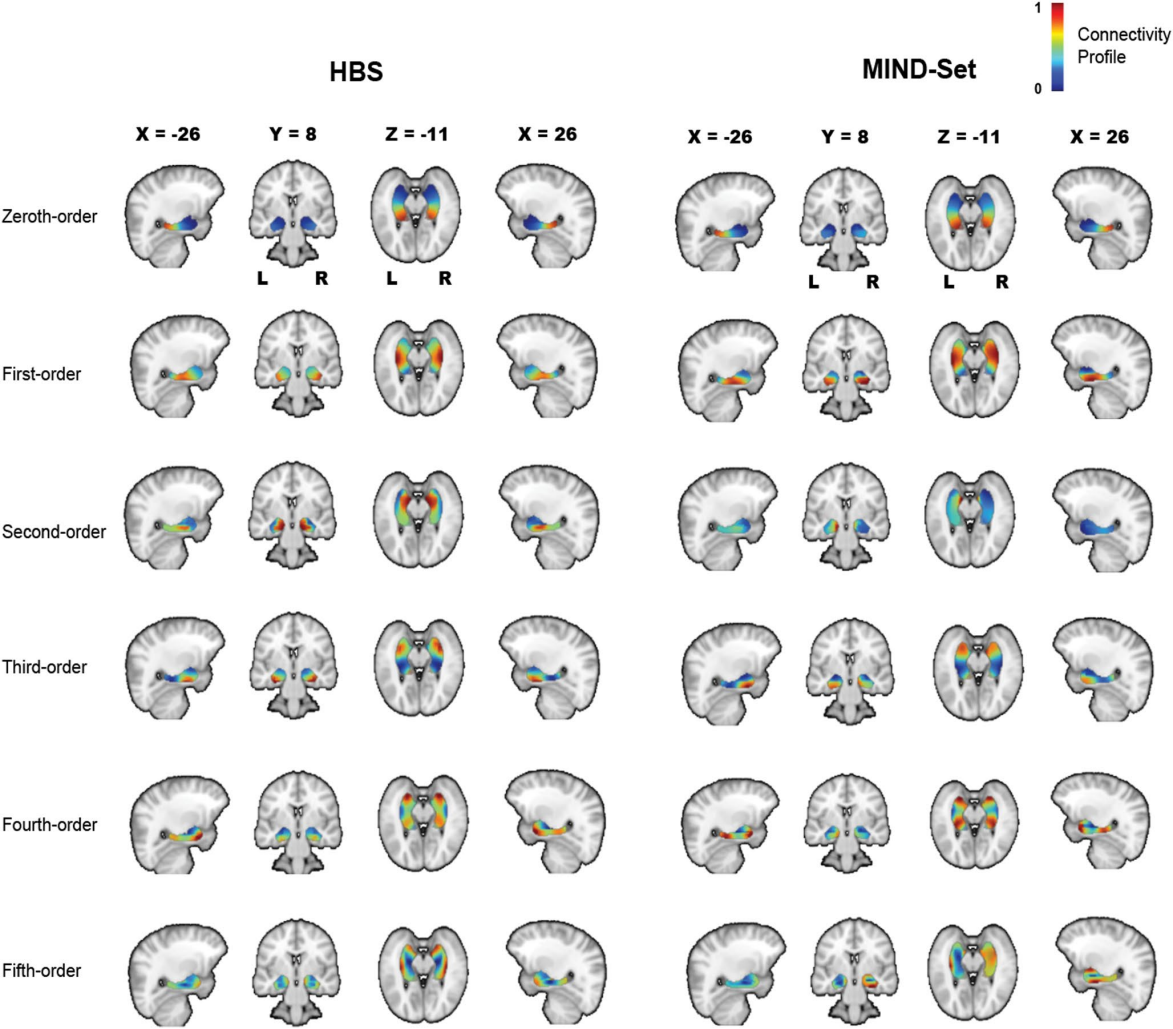
Hippocampus-amygdala gradient maps in the HBS and MIND-Set datasets (the second- and third-order gradients in MIND-Set were swapped for the convenience of comparison to HBS). Gradient values were normalized to the range 0–1, with similar values indicating similar patterns in connectivity profiles. *L* left, *R* right

Across both datasets, we identified six functional connectivity gradients in total (as a sharp decline in the proportion of participants retained after quality assurance was observed at the sixth gradient; Table S5). Figure 1 illustrates the hippocampus-amygdala gradient maps for both datasets. Because gradient maps across different resting states were highly similar (Tables S6 and S7), we visualized the maps derived from resting-state 1 as a representative example. The dominant (zeroth-order) gradient shows a gradual change along the coordinate space, starting in the amygdala and extending along the hippocampal long axis. The first-order gradient is organized from the middle of the hippocampus towards the anterior and posterior ends of the complex. We observed that the second- and third-order gradients appear in reversed order between the HBS and MIND-Set samples, although their overall spatial patterns remain consistent. In the following results, for the convenience of comparison, we used the order in HBS to name gradient maps.

For quality assurance, we excluded participants whose spatial correlation with the group-average gradient maps was lower than 0.5. Table S5 shows the proportion of participants retained based on this threshold. In the HBS dataset, all six connectopic gradients had inclusion rates of 50% or higher. In the MIND-Set dataset, while the fourth-order gradient on the right side during resting-state 2 and the fifth-order gradient bilaterally across all three resting states exhibited lower stability, the first four gradients consistently showed inclusion rates exceeding 50%. The results indicate the robustness of these data-driven connectopic maps, especially for the first four gradients.

### Similarity in spatial layouts between gradient and neurotransmitter maps

**Fig. 2:**
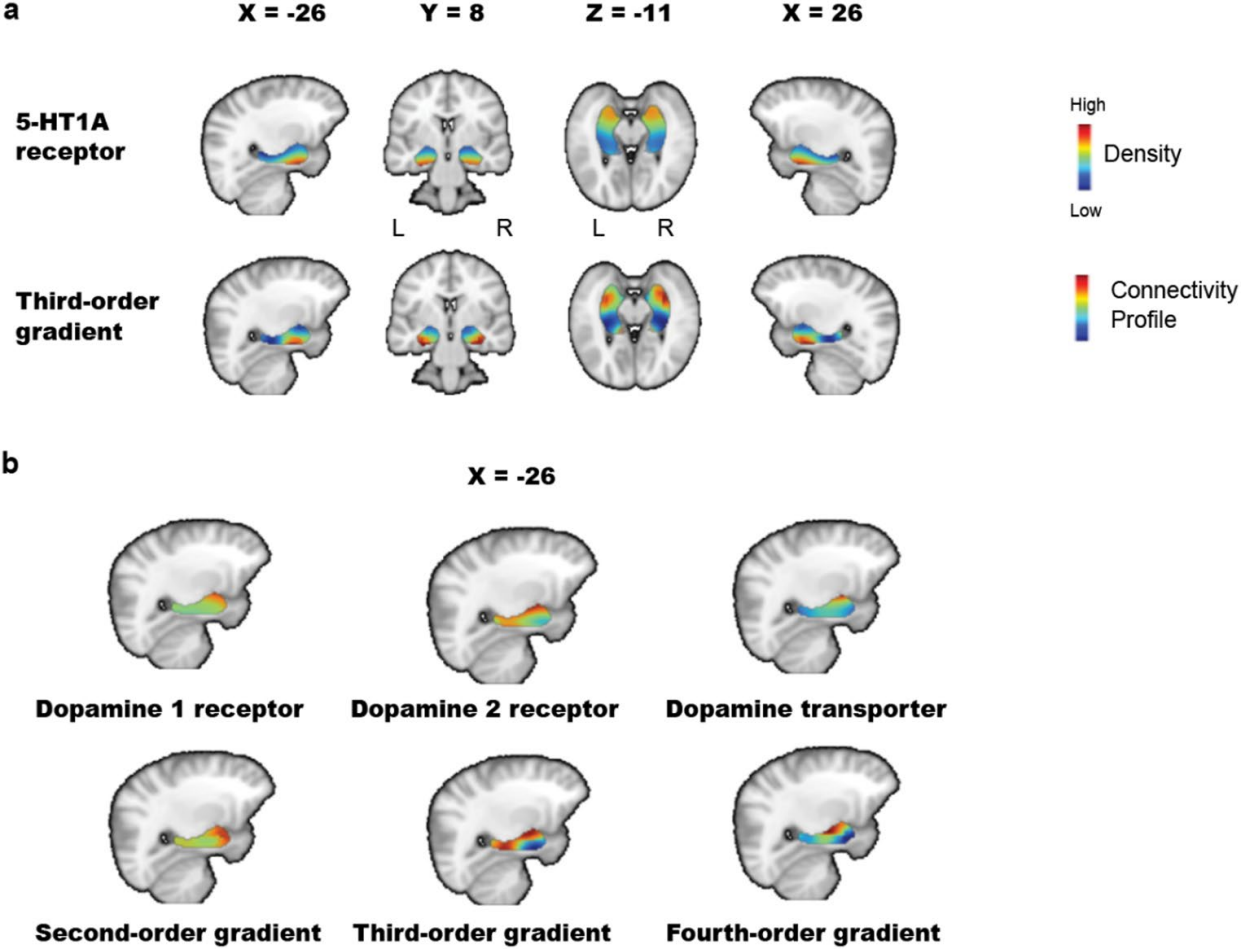
In the hippocampus–amygdala complex, **a** The third-order gradient exhibits spatial patterns similar to those of 5-HT1A receptor maps; **b** Gradients also show spatial similarities with neurotransmitter distributions of the dopamine system: the second-order gradient with dopamine type 1 receptor maps, the third-order gradient (MIND-Set only) with dopamine type 2 receptor maps, and the fourth-order gradient (HBS only) with dopamine transporter maps. *L* left, *R* right

The correlation analysis with permutation testing identified several significant similarities between hippocampus-amygdala gradients and neurotransmitter maps (full statistics in Table S8; also see Fig. S1), with findings highlighting the serotonergic and dopaminergic systems. For the serotonergic system, the third-order gradients showed spatial similarity with the 5-HT1A receptor maps, with significant correlations observed in both datasets (Fig. 2a). For the dopaminergic system, the second-order gradients exhibited similarity with dopamine type 1 (D1) receptor maps on the left side of the complex in both datasets. In MIND-Set only, the third-order gradients showed similarity with dopamine type 2 (D2) receptor maps on the left side. In HBS only, the fourth-order gradients showed similarity with dopamine transporter (DAT) maps on the left side (Fig. 2b). The TSM-based correlations using parametric testing, as well as voxel-wise correlations with gradient values, further supported the spatial similarities between gradients and neurotransmitter layouts described above (Tables S9 and S10). These similarities remained consistent when different TSM model orders were used (Table S11).

Additionally, we split the HBS sample into high- and low-motion subgroups based on mean relative displacement (cutoff = 0.123) and assessed the spatial similarities between gradients and neurotransmitter layouts separately for each subgroup. The results indicated that both subgroups exhibited patterns consistent with those observed in the full sample (Table S12). Similarly, when the MIND-Set sample was divided into psychiatric patients versus healthy controls, or participants with versus without medication, all subgroups showed spatial correspondence patterns comparable to those in the full sample (Tables S13 & S14). These results indicate that the relationship between gradients and neurotransmitter layouts is generally robust to head motion, psychiatric patient versus control status, and medication use.

### Individual gradient-neurotransmitter similarity is associated with mental health outcomes

**Fig. 3:**
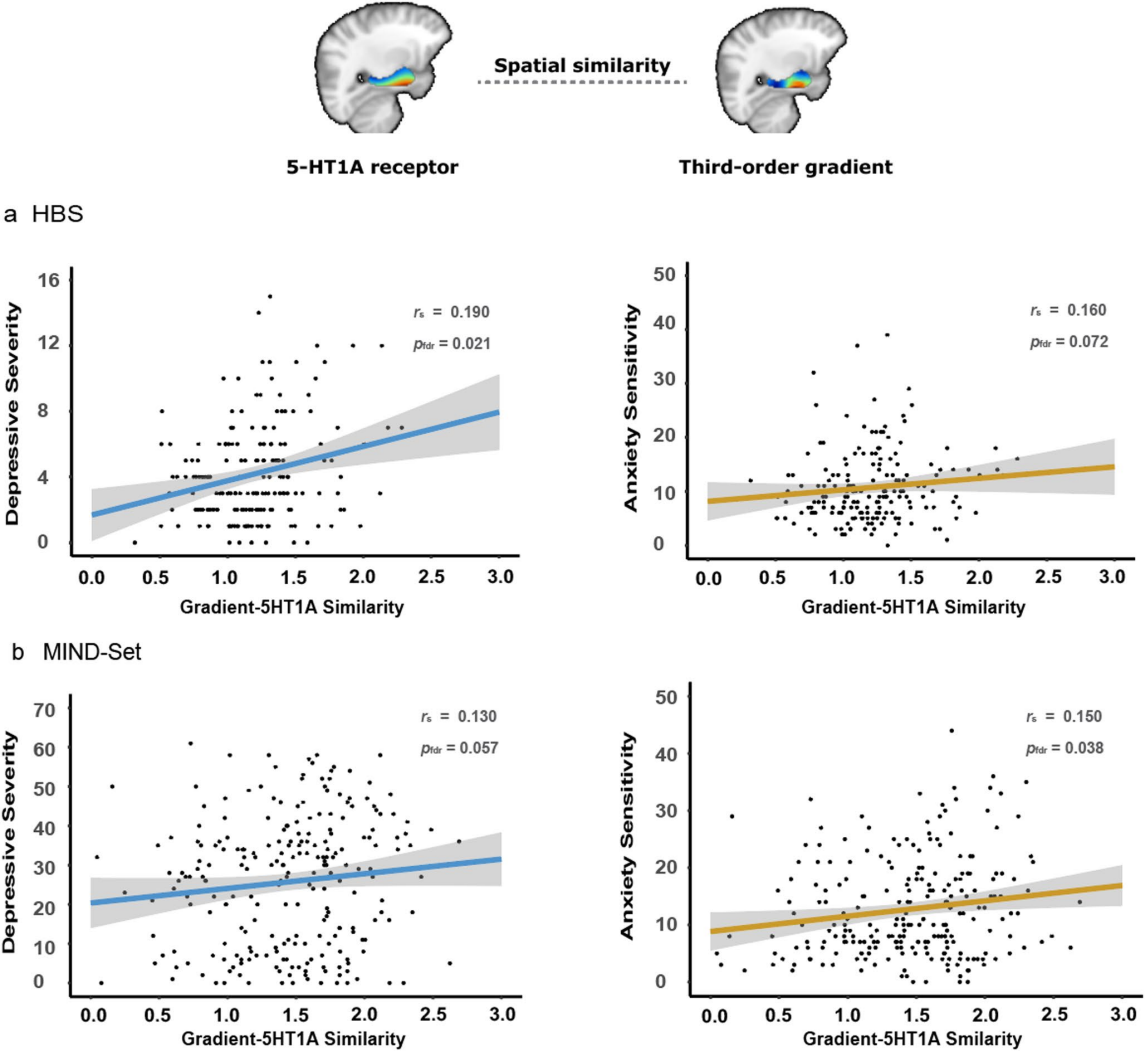
The higher level of spatial similarity between the third-order gradients and 5-HT1A receptor maps (Fisher r-to-z transformed absolute correlation coefficients; the horizontal axis) was associated with higher levels of depressive severity and anxiety sensitivity (the vertical axis). In both datasets, depressive severity was measured as the sum score of IDS-SR, and anxiety sensitivity was assessed with the ASI sum score

For neurotransmitter maps that showed significant spatial similarity with group-average gradients (bilateral 5-HT1A receptors; left D2 receptors in MIND-Set; left D1 receptors; and left DAT in HBS), we computed correlation coefficients between the TSM coefficients of JuSpace PET/SPECT images and individual gradient maps for each participant. Taking Fisher r-to-z transformed absolute correlation coefficients, Spearman correlation analysis revealed several significant associations with anxiety sensitivity and depressive severity. For HBS, the similarity between 5-HT1A receptors and the third-order gradients on the left side of the hippocampus-amygdala complex was positively correlated with depressive severity (*r*_s_ = 0.19, *p*_fdr_ = 0.021), and approaching significance for anxiety sensitivity (*r*_s_ = 0.16, *p*_fdr_ = 0.072). In the MIND-Set dataset, these correlations were observed for the right side (depressive severity: *r*_s_ = 0.13, *p*_fdr_ = 0.057; anxiety sensitivity: *r*_s_ = 0.15, *p*_fdr_ = 0.038; Fig. 3). Given that the two datasets span distinct ranges of anxiety sensitivity and depressive severity (Table S1, also see Fig. S2) —with HBS falling into the relatively lower end and MIND-Set the higher, these replicated correlations may indicate both sensitivity and stability across the symptom spectrum.

As for the dopaminergic system, in MIND-Set, the similarity between D1 receptor and second-order gradient maps (left side) showed a positive correlation with anxiety sensitivity (*r*_s_ = 0.13, *p*_fdr_ = 0.046). This association was not significant in the HBS sample (*r*_s_ = 0.06, *p*_fdr_ = 0.50), although Fisher’s z test comparing the two correlations (e.g., in MIND-Set and HBS) revealed no significant difference (*z* = 0.682, *p* = 0.495). In HBS, the similarity between DAT and fourth-order gradient maps (left side) was negatively associated with anxiety sensitivity (*r*_s_ = – 0.18, *p*_fdr_ = 0.048). Other behavioral variables, including childhood trauma and comorbidity, did not show significant associations with gradient–neurotransmitter correspondence. Full lists of correlations are presented in Tables S15 and S16.

After accounting for age, sex, and head motion (and additionally medication use for the MIND-Set sample), the observed associations exhibited the same patterns (Tables S17 and S18). We also divided the MIND-Set sample into patient and healthy controls, and ran the correlation analyses separately (Table S19). Fisher’s z tests revealed no significant differences in the observed correlations above between two subgroups (*p*s > 0.16).

## Discussion

Widespread functional connections between the hippocampus-amygdala complex and other brain regions play important roles in human cognition and emotion, thereby contributing to the most common psychiatric disorders. In this study, with both healthy and psychiatric cohorts, we (1) identified six distinct connectopic gradients for the hippocampus-amygdala complex, with the first two exhibiting gradual changes along the hippocampal long axis; (2) revealed that neurotransmitters from the serotonin and dopamine systems displayed spatial similarities with connectopic gradients of the hippocampus-amygdala complex; and (3) found that individual variability in these gradient-neurotransmitter similarities was associated with depressive severity and anxiety sensitivity. Our findings highlight the potential of connectopic mapping to bridge functional neuroimaging, biomolecular markers and behavioral measures, offering a novel perspective for understanding brain function and dysfunctions in living human beings.

The first two connectopic gradient maps of the hippocampus–amygdala complex did show gradient changes following the hippocampal long axis. This demonstrates that connectopic mapping can capture the connectivity organization previously identified in animal studies, using fMRI scans of living humans. The spatial organization of our gradient maps also aligns with hippocampal gradients reported in earlier studies (Przeździk et al. 2019; Katsumi et al. 2023; Xie et al. 2024; Nordin et al. 2025). The amygdala exhibited connectivity modes similar to those of the anterior hippocampus, partly because of the spatial proximity, also indicating their close functional coupling. While previous research focused on lower-order connectopic gradients, our study extended it by extracting higher-order gradient maps and revealing their biological significance through links with neurotransmitter distributions. Notably, our findings in the hippocampus–amygdala complex gradient are reproducible across both healthy and psychiatric populations, which contribute to the understanding of transdiagnostic generalizability of the connectopic mapping approach. For small and curved subcortical structures such as the hippocampus and amygdala, one would suspect that the partial volume effect (PVE) could be a potential confound in deriving biologically meaningful gradients, and the use of multiband acceleration in both datasets may introduce further challenges to the temporal signal-to-noise ratio. However, effective denoising strategies applied during preprocessing (e.g., FIX or ICA-AROMA), together with the inherent robustness of ConGrads to voxel-level noise, systematically mitigated the influence of these factors on our results, which is further supported by the reproducibility of gradient maps across individuals and different populations.

Various neurotransmitters contribute to regulating the functionality of the hippocampus and amygdala. Connectopic mapping offers an approach to better understand the relationship between neurotransmitters and hippocampus-amygdala functional connectivity organization, by breaking down overall connectivity patterns into specific topographical gradients. For each identified gradient, we evaluated its similarity in spatial layouts with various neurotransmitter maps, which could serve as an indirect and preliminary measure of the potential influence of each neurotransmitter on functional connectivity organization and help to understand biological features of these gradients.

We found a reproducible spatial similarity between the third-order gradients and 5-HT1A receptor maps. 5-HT is a monoamine neurotransmitter synthesized in both the central nervous system and gastrointestinal cells (Jonnakuty and Gragnoli 2008). The 5-HT1A receptor is one of the most abundant receptor subtypes in the mammalian brain (Popova and Naumenko 2013). In the hippocampus and amygdala, most 5-HT1A receptors function as postsynaptic receptors suppressing pyramidal cell firing (Ögren et al. 2008). Our third-order gradient generally captured the 5-HT1A receptor distribution within the hippocampus-amygdala complex, suggesting a close relationship between functional connectivity organization of the complex and the serotonergic system. In addition to previous reports from animal and PET studies (Jovanovic et al. 2011; Gener et al. 2019), our findings provide evidence for the feasibility of receiving serotonin receptor related readouts from resting-state fMRI scans on an individual basis.

The activity of 5-HT1A receptors is implicated in emotion processing and stress coping (Celada et al. 2013; Puglisi-Allegra and Andolina 2015), with alterations shown in mood disorders. Studies have reported increased hippocampal 5-HT1A receptor binding potential in individuals with depressive episodes and childhood adversity (Bartlett et al. 2023), and elevated 5-HT1A receptor density in people who committed suicide (Underwood et al. 2018). Higher 5-HT1A receptor binding potential was also associated with poor responses to antidepressant treatment (Parsey et al. 2006). In this study, we found that greater similarity between the third-order gradients and 5-HT1A receptor maps was related to more severe depressive symptoms and heightened anxiety sensitivity. Together with previous findings linking elevated 5-HT1A receptor involvement to internalizing psychiatric symptoms, these results suggest that alterations in 5-HT1A receptor activity, as reflected in its relationship with the functional connectivity organization of the hippocampus–amygdala complex, may serve as a potential risk factor underlying mood disorder symptoms. In contrast, individual gradient-to-5-HT1A similarity did not appear to reflect childhood trauma, indicating that this spatial correspondence might more sensitively capture current symptomatology than imprints of past experiences. Notably, this relationship with symptomatology was observed in both the healthy sample (with a narrower symptom range) and the clinical sample (with a broader range), suggesting its sensitivity to transdiagnostic symptom variation. Compared to anatomical or PET studies, connectopic mapping could be a more efficient and cost-effective approach for depicting this alteration in 5-HT1A receptor functionality.

Beyond the serotonergic system, our findings also identified similarities between hippocampus-amygdala gradients and the spatial organization of dopamine-related neurotransmitters: D1 receptors with the second-order gradient, D2 receptors with the third-order gradient, DAT with the fourth-order gradient. D1 and D2 receptors are the most abundant dopamine receptor subtypes (Ayano 2016). D1 receptors have an excitatory role in signaling pathways, whereas D2 receptors exhibit more complex effects, generally with inhibitory properties (Grilli et al. 1988; Beaulieu and Gainetdinov 2011). Together with other dopamine receptors, D1 and D2 receptors coordinate hippocampal plasticity and influence learning and affective behaviors (Edelmann and Lessmann 2018). DAT regulates dopamine reuptake and maintains homeostasis (Madras et al. 2005). These results highlight the role of dopamine system in functional connectivity organization of the hippocampus-amygdala complex. Moreover, we observed that greater gradient-D1 map similarity was associated with higher anxiety sensitivity, while gradient-DAT map similarity showed an inverse relationship. Studies showed the anxiogenic effects of D1 receptor activation; for instance, infusing D1 agonists into the amygdala was proved to induce heightened anxiety behaviors (Guarraci et al. 1999; de la Mora et al. 2010). Reduced DAT availability has been linked to greater anxiety severity in Parkinson’s disease patients (Erro et al. 2012). The correlations between D1, DAT and anxiety sensitivity did align with the previous literatures, indicating alterations in the dopamine system may contribute to anxiety symptoms partly through its association with the functional connectivity organization of the hippocampus-amygdala complex.

Our study has several limitations. First, the neurotransmitter maps used were templates derived from previous studies, rather than data measured directly within HBS and MIND-Set. Heterogeneity across these templates including tracers and reconstruction methods, may have introduced potential confounds in the comparisons. Datasets that include both resting-state fMRI and PET/SPECT scans, with carefully controlled acquisition and processing of neurotransmitter maps, could provide a more sensitive approach and also serve to validate our findings. Second, the interpretation of relationships between gradients and neurotransmitters remains indirect. The observed spatial correspondences may reflect the modulatory influence of neurotransmitters on functional connectivity organization, but other factors such as shared cytoarchitecture, vasculature, myelination, or signal dropout patterns, could also contribute to these similarities. Future studies incorporating multimodal data (e.g., microstructural imaging measures), will be important for disentangling these contributions and determining whether the observed associations remain robust after accounting for these confounds. Third, the correlations between individual gradient–neurotransmitter similarities and mental health measures were relatively small in effect size. This may be partly due to the use of template-based rather than individual-specific neurotransmitter maps. Nevertheless, these exploratory and preliminary findings should be interpreted with caution. Finally, our study provides some evidence for the stability of gradients and their biological features with respect to medication or diagnostic status. Despite this, future research such as longitudinal studies in specific medication groups or designs specifically targeting patient–control comparisons, will further clarify the effects of medication and psychiatric status on functional connectivity gradients, thereby facilitating their translational application in clinical practice.

In conclusion, across both healthy and psychiatric cohorts, our study provides an overview of functional connectivity topography of the hippocampus–amygdala complex. Certain topographic gradients showed spatial correspondence with neurotransmitter distributions from the serotonergic and dopaminergic systems, with individual differences in this correspondence associated with depressive and anxiety symptoms. As an emerging analytical method, connectopic mapping demonstrates promise as a biomarker for assessing neurotransmitter related psychiatric symptomatology with resting- state fMRI.

## Supplementary Information

Below is the link to the electronic supplementary material.

Supplementary Material 1

## Data Availability

The processed data and codes are archived in the institutional repository of the Donders Institute for Brain, Cognition and Behavior, and available upon reasonable request.
