# Supplementary material for: Characterizing functional connectivity gradients for the hippocampus–amygdala complex in healthy and psychiatric cohorts
Source: Brain Struct Funct. 2026 Jun 3;231(5):80. doi: 10.1007/s00429-026-03134-4 (PMC13233971; doi:10.1007/s00429-026-03134-4)
Supplement: Supplementary file 1 — Supplementary Material 1 [file 429_2026_3134_MOESM1_ESM.docx]

**Supplementary Material**

**Table S1.** Sample characteristics for HBS and MIND-Set

|  | **Sample size** | **Age** | **Gender** | **Anxiety Sensitivity** | **Depressive Severity** | **Comorbidity** |
| --- | --- | --- | --- | --- | --- | --- |
| **HBS** | 410 | 33.8 ± 2.8 | 169 Males | 10.9 ± 6.9 | 4.4 ± 3.3 | -- |
| **MIND-Set** | 367 | 37.6 ± 14.0 | 202 Males | 12.7 ± 9.0 | 25.5 ± 16.6 | 1.6 ± 1.2 |
| **Difference** | -- | *t* = -4.72,  *p* < .001 | Chi-suqare = 17.69,  *p* < .001 | *t* = -2.80,  *p* = .005 | *t* = -22.50,  *p* < .001 | -- |

In MIND-Set, 286 participants were psychiatric patients: Mood Disorder = 225, Anxiety Disorder (including Obsessive Compulsive Disorders (OCD) and PTSD) = 80, Attention-Deficit/Hyperactivity Disorder (ADHD) = 110, Autism Spectrum Disorder (ASD) = 91, Addiction = 75. In both datasets, depressive severity was measured as the sum score of IDS-SR, and anxiety sensitivity was assessed with the ASI sum score. Comorbidity in MIND-Set was indexed by the number of diagnosed psychiatric disorder clusters per participant.

**Table S2.** Nervous system medication use in the MIND-Set sample

|  | **N01** | **N02** | **N03** | **N04** | **N05** | **N06** | **N07** | **N01/ N02/ N03/ N04/ N05/ N06/ N07** | **N04B** | **N05A** | **N05B** | **N06A** |
| --- | --- | --- | --- | --- | --- | --- | --- | --- | --- | --- | --- | --- |
| **Number of subjects** | 1 | 39 | 8 | 3 | 73 | 103 | 0 | 150 | 1 | 30 | 39 | 86 |

Medication use was categorized according to the Anatomical Therapeutic Chemical (ATC) Classification System, category N: N01, anesthetics. N02, analgesics. N03, antiepileptics. N04 anti-Parkinson drugs. N05, psycholeptics. N06, psychoanaleptics. N07, other nervous system drugs. N04B, dopaminergic agents. N05A, antipsychotics. N05B, anxiolytics. N06A, antidepressants. In the sample, 150 participants used at least one of N0101/ N02/ N03/ N04/ N05/ N06/ N07. When compared with the frequency for each individual category, this indicates a substantial presence of co-medication within the sample.

**Table S3.** Neurotransmitter templates included from JuSpace

|  | **Template** | **Tracer** | **Provenance** |
| --- | --- | --- | --- |
| **5HT 1A receptor** | WAY_HC36 | [carbonyl-^11^C]WAY-100635 | (Savli et al. 2012)  https://identifiers.org/neurovault.collection:1206 |
| **5HT 1B receptor** | P943_HC22 | [^11^C]P943 | (Savli et al. 2012)  ^https://identifiers.org/neurovault.collection:1206^ |
| **5HT 2A receptor** | ALT_HC19 | [^18^F]altanserin | (Savli et al. 2012)  ^https://identifiers.org/neurovault.collection:1206^ |
| **5HT 4 receptor** | sb20_hc59_beliveau | [^11^C]SB207145 | (Beliveau et al. 2017) |
| **5HT transporter** | DASB_HC30 | [^11^C]DASB | (Savli et al. 2012)  ^https://identifiers.org/neurovault.collection:1206^ |
| **5HT transporter** | MADAM_c11 | [^11^C]MADAM | (Fazio et al. 2016) |
| **Dopamine type 1 receptor** | SCH23390_c11 | [^11^C]SCH23390 | (Kaller et al. 2017) |
| **Dopamine type 2 receptor** | RACLOPRIDE_c11 | [^11^C]raclopride | (Alakurtti et al. 2015) |
| **Dopamine transporter** | DATSPECT | DaTSCAN | (Dukart et al. 2018) |
| **F-Dopa** | FDOPA_f18 | [^18^F]FDOPA | (Gómez et al. 2018) |
| **GABA_A_ receptor** | FLUMAZENIL_c11 | [^11C^]flumazenil | (Dukart et al. 2018) |
| **Kappa-opioid receptor** | KappaOp_LY2795050_10m_ShokriKojori | [^11^C]LY2795050 | (Shokri‐Kojori et al. 2022) |
| **Mu-opioid receptor** | CARFENTANIL_c11 | [^11^C]carfentanil | (Kantonen et al. 2020)  https://identifiers.org/neurovault.image:303255 |
| **Metabotropic glutamate receptor 5** | abp_hc28_dubois | [^11^C]ABP688 | (DuBois et al. 2016) |
| **Cannabinoid receptor 1** | FMPEPd2_hc22_laurikainen | [^18^F]FMPEP-d2 | (Laurikainen et al. 2019) |
| **Noradrenaline transporter** | MRB_c11 | S,S-[^11^C]O-methylreboxetine | (Hesse et al. 2017) |
| **NMDA receptor** | ge179_29hc_galovic2021 | [^18^F]GE-179 | (Galovic et al. 2021) |
| **Vesicular acetylcholine transporter** | feobv_hc4_tuominen | [^18^F]-FEOBV | (Hansen et al. 2022) |

Scan templates for 5HT1A receptor, 5HT1B receptor, 5HT2A receptor, 5HT transporter, dopamine type 1 receptor, dopamine type 2 receptor, dopamine transporter, F-Dopa, GABAA receptor, noradrenaline transporter, and mu opiod receptor were included following Oldehinkel et al. (2022). Additional templates were selected based on their widespread use in neuroimaging studies across both healthy and clinical populations and their demonstrated stability and reliability in representing the spatial distribution of the respective receptors.

**Table S4.** Regional variance metrics within the hippocampus–amygdala complex for all included neurotransmitter templates

|  | **Template** | **Left** | | | | **Right** | | | |
| --- | --- | --- | --- | --- | --- | --- | --- | --- | --- |
|  |  | Mean | SD | CV | Range | Mean | SD | CV | Range |
| 5HT 1A receptor | WAY_HC36 | 41.720 | 19.478 | 0.467 | 101.391 | 39.278 | 19.673 | 0.501 | 100.234 |
| 5HT 1B receptor | P943_HC22 | 8.276 | 2.949 | 0.356 | 25.471 | 6.439 | 2.711 | 0.421 | 22.596 |
| 5HT 2A receptor | ALT_HC19 | 20.055 | 9.460 | 0.472 | 55.719 | 18.711 | 9.123 | 0.488 | 52.328 |
| 5HT 4 receptor | sb20_hc59_beliveau | 5.702 | 2.064 | 0.362 | 12.941 | 5.078 | 1.758 | 0.346 | 11.233 |
| 5HT transporter | DASB_HC30 | 12.249 | 4.884 | 0.399 | 33.476 | 10.472 | 5.826 | 0.556 | 31.639 |
| 5HT transporter | MADAM_c11 | 13.258 | 4.872 | 0.368 | 30.534 | 15.325 | 10.738 | 0.701 | 53.218 |
| Dopamine type 1 receptor | SCH23390_c11 | 12.373 | 4.425 | 0.358 | 26.824 | 12.468 | 4.395 | 0.352 | 29.914 |
| Dopamine type 2 receptor | RACLOPRIDE_c11 | 18.497 | 1.381 | 0.075 | 8.381 | 18.122 | 1.404 | 0.077 | 12.150 |
| Dopamine transporter | DATSPECT | 36.519 | 5.204 | 0.142 | 37.531 | 35.682 | 5.384 | 0.151 | 42.444 |
| F-Dopa | FDOPA_f18 | 35.580 | 6.215 | 0.175 | 41.118 | 35.206 | 6.261 | 0.178 | 45.157 |
| GABA_A_ receptor | FLUMAZENIL_c11 | 52.059 | 8.320 | 0.160 | 54.825 | 49.413 | 8.398 | 0.170 | 51.768 |
| Kappa-opioid receptor | KappaOp_LY2795050_10m_ShokriKojori | 30.626 | 16.521 | 0.539 | 89.850 | 30.763 | 17.000 | 0.553 | 86.961 |
| Mu-opioid receptor | CARFENTANIL_c11 | 20.481 | 12.388 | 0.605 | 74.093 | 19.184 | 12.683 | 0.661 | 73.443 |
| Metabotropic glutamate receptor 5 | abp_hc28_dubois | 52.417 | 9.336 | 0.178 | 63.788 | 49.316 | 9.145 | 0.185 | 63.002 |
| Cannabinoid receptor 1 | FMPEPd2_hc22_laurikainen | 40.324 | 5.613 | 0.139 | 43.358 | 39.946 | 6.186 | 0.155 | 51.526 |
| Noradrenaline transporter | MRB_c11 | 8.251 | 3.024 | 0.367 | 25.614 | 7.972 | 2.842 | 0.357 | 19.279 |
| NMDA receptor | ge179_29hc_galovic2021 | 56.496 | 6.349 | 0.112 | 42.635 | 55.166 | 6.383 | 0.116 | 42.666 |
| Vesicular acetylcholine transporter | feobv_hc4_tuominen | 16.905 | 3.135 | 0.185 | 21.880 | 16.354 | 3.259 | 0.199 | 26.052 |

CV, coefficient of variation.

**Table S5.** The proportions of participants whose spatial correlation with group-average gradients higher than 0.5.

|  |  | **Resting-State1_Left** | **Resting-State1_Right** | **Resting-State2_Left** | **Resting-State2_Right** | **Resting-State3_Left** | **Resting-State3_Right** |
| --- | --- | --- | --- | --- | --- | --- | --- |
| **HBS** | **Zeroth-order** | 99.727% | 99.724% | 100.000% | 99.656% | 100.000% | 98.958% |
|  | **First-order** | 99.454% | 99.724% | 99.308% | 98.969% | 98.592% | 97.917% |
|  | **Second-order** | 96.721% | 94.751% | 96.540% | 92.440% | 94.014% | 90.972% |
|  | **Third-order** | 93.716% | 95.580% | 95.502% | 93.814% | 93.662% | 93.403% |
|  | **Fourth-order** | 86.339% | 80.939% | 86.159% | 79.381% | 80.986% | 77.083% |
|  | **Fifth-order** | 58.470% | 55.801% | 53.633% | 53.608% | 53.873% | 50.000% |
| **MIND-Set** | **Zeroth-order** | 98.638% | 98.093% | 99.454% | 98.093% | 99.725% | 98.630% |
|  | **First-order** | 96.458% | 95.913% | 95.902% | 93.733% | 97.802% | 95.068% |
|  | **Second-order** | 91.826% | 87.738% | 89.344% | 75.749% | 91.758% | 82.192% |
|  | **Third-order** | 87.738% | 83.651% | 86.066% | 78.202% | 92.308% | 82.740% |
|  | **Fourth-order** | 70.845% | 75.477% | 69.945% | 8.719% | 75.549% | 73.699% |
|  | **Fifth-order** | 22.343% | 5.177% | 9.016% | 6.267% | 5.220% | 4.658% |

**Table S6.** Similarity (correlation coefficients) between gradients from resting-state 1 and resting-state 2 or 3, shown separately for same-order and different-order gradients.

|  |  | **Resting-State2_Left** | | **Resting-State3_Left** | | **Resting-State2_Right** | | **Resting-State3_Right** | |
| --- | --- | --- | --- | --- | --- | --- | --- | --- | --- |
|  |  | same | different | same | different | same | different | same | different |
| **HBS** | **Zeroth-order** | 1.000***  *p* < .001 | 0.371 | 1.000***  *p* < .001 | 0.374 | 1.000***  *p* < .001 | 0.409 | 1.000***  *p* < .001 | 0.374 |
|  | **First-order** | 0.998***  *p* < .001 | 0.304 | 0.999***  *p* < .001 | 0.308 | 0.993***  *p* < .001 | 0.330 | 0.999***  *p* < .001 | 0.309 |
|  | **Second-order** | 0.987***  *p* < .001 | 0.060 | 0.990***  *p* < .001 | 0.078 | 0.998***  *p* < .001 | 0.184 | 0.995***  *p* < .001 | 0.168 |
|  | **Third-order** | 0.992***  *p* < .001 | 0.336 | 0.994***  *p* < .001 | 0.336 | 0.999***  *p* < .001 | 0.310 | 0.998***  *p* < .001 | 0.319 |
|  | **Fourth-order** | 0.974***  *p* < .001 | 0.161 | 0.963***  *p* < .001 | 0.171 | 0.990***  *p* < .001 | 0.118 | 0.998***  *p* < .001 | 0.132 |
|  | **Fifth-order** | 0.986***  *p* < .001 | 0.091 | 0.959***  *p* < .001 | 0.093 | 0.982***  *p* < .001 | 0.141 | 0.995***  *p* < .001 | 0.134 |
| **MIND-Set** | **Zeroth-order** | 1.000***  *p* < .001 | 0.397 | 1.000***  *p* < .001 | 0.476 | 0.998***  *p* < .001 | 0.524 | 0.999***  *p* < .001 | 0.450 |
|  | **First-order** | 1.000***  *p* < .001 | 0.332 | 0.999***  *p* < .001 | 0.244 | 0.994***  *p* < .001 | 0.336 | 0.996***  *p* < .001 | 0.355 |
|  | **Second-order** | 1.000***  *p* < .001 | 0.238 | 1.000***  *p* < .001 | 0.299 | 0.777*  *p* = .014 | 0.616 | 0.976***  *p* < .001 | 0.561 |
|  | **Third-order** | 0.999***  *p* < .001 | 0.067 | 0.996***  *p* < .001 | 0.105 | 0.927***  *p* < .001 | 0.378 | 0.976***  *p* < .001 | 0.394 |
|  | **Fourth-order** | 1.000***  *p* < .001 | 0.309 | 0.996***  *p* < .001 | 0.350 | 0.896**  *p* = .001 | 0.404 | 0.990***  *p* < .001 | 0.294 |
|  | **Fifth-order** | 0.586 | 0.175 | 0.408 | 0.178 | 0.610 | 0.312 | 0.987***  *p* < .001 | 0.288 |

Gradients from resting-state 2 or 3 were compared with those from resting-state 1. Trend-surface model coefficients were used to compute Pearson correlation coefficients, calculated separately for gradients of the same order (e.g., zeroth-order gradients from resting-state 1 and resting-state 2) and for gradients of different orders (e.g., the average correlation between zeroth-order gradients from resting-state 1 and all other orders from resting-state 2). ***p*<0.01, ****p*<0.001.

**Table S7.** Similarity (correlation coefficients) between gradients from HBS

and MIND-Set, shown separately for same-order and different-order gradients.

|  | **Resting-State1** | | **Resting-State2** | | **Resting-State3** | |
| --- | --- | --- | --- | --- | --- | --- |
|  | same | different | same | different | same | different |
| **Zeroth-order** | 0.995 ***  *p* < .001 | 0.414 | 0.996 ***  *p* < .001 | 0.390 | 0.995 ***  *p* < .001 | 0.468 |
| **First-order** | 0.802 **  *p* = .009 | 0.446 | 0.825 **  *p* = .006 | 0.463 | 0.805 **  *p* = .009 | 0.364 |
| **Second-order** | 0.942 ***  *p* < .001 | 0.158 | 0.920 ***  *p* < .001 | 0.162 | 0.904 **  *p* = .001 | 0.193 |
| **Third-order** | 0.942 ***  *p* < .001 | 0.348 | 0.967 ***  *p* < .001 | 0.318 | 0.963 ***  *p* < .001 | 0.387 |
| **Fourth-order** | 0.745 *  *p* = .021 | 0.224 | 0.852 **  *p* = .004 | 0.268 | 0.851 **  *p* = .004 | 0.289 |
| **Fifth-order** | 0.842 **  *p* = .009 | 0.098 | 0.293  *p* = .444 | 0.063 | 0.479  *p* = .192 | 0.084 |

Trend-surface model coefficients were used to compute Pearson correlation coefficients (using the left side as an example), calculated separately for gradients of the same order (e.g., zeroth-order gradients from HBS and MIND-Set) and for gradients of different orders (e.g., the average correlation between zeroth-order gradients from HBS and all other orders from MIND-Set). **p*<0.05, ***p*<0.01, ****p*<0.001.

**Table S8**. Correlations coefficients (Fisher r-to-z transformed, absolute values, with FDR correction) between gradient and neurotransmitter layouts

|  |  |  | **HBS**  **Resting-State1** | **HBS**  **Resting-State2** | **HBS**  **Resting-State3** | **MIND-Set**  **Resting-State1** | **MIND-Set**  **Resting-State2** | **MIND-Set**  **Resting-State3** |
| --- | --- | --- | --- | --- | --- | --- | --- | --- |
| **Zeroth-order gradient_Left** | 5HT1A | *z* | 1.049 | 1.033 | 1.041 | 0.903 | 0.896 | 0.901 |
|  |  | *p* | 0.926 | 0.929 | 0.937 | 0.902 | 0.903 | 0.909 |
|  | 5HT1B | *z* | 0.311 | 0.318 | 0.317 | 0.369 | 0.374 | 0.372 |
|  |  | *p* | 0.926 | 0.929 | 0.937 | 0.902 | 0.906 | 0.909 |
|  | 5HT2A | *z* | 0.057 | 0.055 | 0.061 | 0.018 | 0.022 | 0.026 |
|  |  | *p* | 0.980 | 0.981 | 0.971 | 0.973 | 0.978 | 0.970 |
|  | 5HT4 | *z* | 0.877 | 0.869 | 0.880 | 0.799 | 0.802 | 0.806 |
|  |  | *p* | 0.926 | 0.929 | 0.937 | 0.902 | 0.903 | 0.909 |
|  | CB1 | *z* | 0.727 | 0.727 | 0.732 | 0.696 | 0.699 | 0.703 |
|  |  | *p* | 0.926 | 0.929 | 0.937 | 0.902 | 0.903 | 0.909 |
|  | D1 | *z* | 0.211 | 0.215 | 0.218 | 0.243 | 0.251 | 0.252 |
|  |  | *p* | 0.978 | 0.973 | 0.965 | 0.902 | 0.906 | 0.909 |
|  | D2 | *z* | 0.813 | 0.798 | 0.804 | 0.699 | 0.692 | 0.694 |
|  |  | *p* | 0.926 | 0.929 | 0.937 | 0.902 | 0.903 | 0.909 |
|  | DAT | *z* | 0.100 | 0.112 | 0.104 | 0.179 | 0.180 | 0.180 |
|  |  | *p* | 0.978 | 0.976 | 0.971 | 0.902 | 0.906 | 0.909 |
|  | FDOPA | *z* | 0.459 | 0.466 | 0.468 | 0.504 | 0.512 | 0.514 |
|  |  | *p* | 0.926 | 0.929 | 0.937 | 0.902 | 0.903 | 0.909 |
|  | GABAa | *z* | 0.568 | 0.558 | 0.567 | 0.475 | 0.475 | 0.478 |
|  |  | *p* | 0.926 | 0.929 | 0.937 | 0.902 | 0.903 | 0.909 |
|  | KappaOp | *z* | 0.728 | 0.733 | 0.733 | 0.809 | 0.817 | 0.810 |
|  |  | *p* | 0.926 | 0.929 | 0.937 | 0.902 | 0.903 | 0.909 |
|  | mGluR5 | *z* | 0.622 | 0.611 | 0.622 | 0.532 | 0.532 | 0.535 |
|  |  | *p* | 0.926 | 0.929 | 0.937 | 0.902 | 0.903 | 0.909 |
|  | MU | *z* | 0.383 | 0.387 | 0.387 | 0.454 | 0.461 | 0.455 |
|  |  | *p* | 0.926 | 0.929 | 0.937 | 0.902 | 0.903 | 0.909 |
|  | NA | *z* | 0.009 | 0.013 | 0.008 | 0.043 | 0.040 | 0.039 |
|  |  | *p* | 0.988 | 0.983 | 0.989 | 0.973 | 0.978 | 0.970 |
|  | NMDA | *z* | 0.152 | 0.157 | 0.149 | 0.178 | 0.173 | 0.172 |
|  |  | *p* | 0.978 | 0.973 | 0.971 | 0.902 | 0.906 | 0.909 |
|  | SERT_DAS | *z* | 0.688 | 0.695 | 0.687 | 0.745 | 0.742 | 0.737 |
|  |  | *p* | 0.926 | 0.929 | 0.937 | 0.902 | 0.903 | 0.909 |
|  | SERT_MADAM | *z* | 0.506 | 0.514 | 0.510 | 0.588 | 0.591 | 0.586 |
|  |  | *p* | 0.926 | 0.929 | 0.937 | 0.902 | 0.903 | 0.909 |
|  | VACh | *z* | 0.350 | 0.339 | 0.344 | 0.260 | 0.255 | 0.258 |
|  |  | *p* | 0.926 | 0.929 | 0.937 | 0.902 | 0.906 | 0.909 |
| **Zeroth-order gradient_Right** | 5HT1A | *z* | 1.053 | 1.044 | 1.040 | 0.792 | 0.716 | 0.760 |
|  |  | *p* | 0.777 | 0.766 | 0.767 | 0.897 | 0.892 | 0.900 |
|  | 5HT1B | *z* | 0.118 | 0.120 | 0.119 | 0.227 | 0.255 | 0.244 |
|  |  | *p* | 1.000 | 1.000 | 0.995 | 0.897 | 0.892 | 0.900 |
|  | 5HT2A | *z* | 0.050 | 0.052 | 0.053 | 0.098 | 0.121 | 0.110 |
|  |  | *p* | 1.000 | 1.000 | 0.995 | 0.897 | 0.892 | 0.900 |
|  | 5HT4 | *z* | 0.619 | 0.612 | 0.613 | 0.476 | 0.420 | 0.449 |
|  |  | *p* | 0.777 | 0.766 | 0.767 | 0.897 | 0.892 | 0.900 |
|  | CB1 | *z* | 0.478 | 0.475 | 0.473 | 0.415 | 0.382 | 0.403 |
|  |  | *p* | 0.777 | 0.766 | 0.767 | 0.897 | 0.892 | 0.900 |
|  | D1 | *z* | 0.037 | 0.036 | 0.039 | 0.088 | 0.094 | 0.087 |
|  |  | *p* | 1.000 | 1.000 | 0.995 | 0.897 | 0.892 | 0.900 |
|  | D2 | *z* | 0.498 | 0.498 | 0.494 | 0.358 | 0.322 | 0.342 |
|  |  | *p* | 0.777 | 0.766 | 0.767 | 0.897 | 0.892 | 0.900 |
|  | DAT | *z* | 0.000 | 0.000 | 0.005 | 0.134 | 0.171 | 0.147 |
|  |  | *p* | 1.000 | 1.000 | 0.995 | 0.897 | 0.892 | 0.900 |
|  | FDOPA | *z* | 0.652 | 0.655 | 0.653 | 0.730 | 0.739 | 0.736 |
|  |  | *p* | 0.777 | 0.766 | 0.767 | 0.897 | 0.892 | 0.900 |
|  | GABAa | *z* | 0.349 | 0.347 | 0.341 | 0.190 | 0.139 | 0.172 |
|  |  | *p* | 0.886 | 0.897 | 0.896 | 0.897 | 0.892 | 0.900 |
|  | KappaOp | *z* | 1.015 | 1.025 | 1.019 | 1.179 | 1.224 | 1.215 |
|  |  | *p* | 0.777 | 0.766 | 0.767 | 0.897 | 0.892 | 0.886 |
|  | mGluR5 | *z* | 0.511 | 0.509 | 0.502 | 0.343 | 0.290 | 0.324 |
|  |  | *p* | 0.777 | 0.766 | 0.767 | 0.897 | 0.892 | 0.900 |
|  | MU | *z* | 0.470 | 0.479 | 0.473 | 0.597 | 0.643 | 0.628 |
|  |  | *p* | 0.777 | 0.766 | 0.767 | 0.897 | 0.892 | 0.900 |
|  | NA | *z* | 0.259 | 0.252 | 0.263 | 0.275 | 0.273 | 0.265 |
|  |  | *p* | 0.936 | 0.937 | 0.930 | 0.897 | 0.892 | 0.900 |
|  | NMDA | *z* | 0.501 | 0.505 | 0.505 | 0.601 | 0.641 | 0.619 |
|  |  | *p* | 0.777 | 0.766 | 0.767 | 0.897 | 0.892 | 0.900 |
|  | SERT_DAS | *z* | 0.721 | 0.730 | 0.724 | 0.866 | 0.917 | 0.905 |
|  |  | *p* | 0.777 | 0.766 | 0.767 | 0.897 | 0.892 | 0.900 |
|  | SERT_MADAM | *z* | 0.908 | 0.919 | 0.909 | 1.050 | 1.097 | 1.095 |
|  |  | *p* | 0.777 | 0.766 | 0.767 | 0.897 | 0.892 | 0.886 |
|  | VACh | *z* | 0.522 | 0.519 | 0.516 | 0.354 | 0.307 | 0.333 |
|  |  | *p* | 0.777 | 0.766 | 0.767 | 0.897 | 0.892 | 0.900 |
| **First-order gradient_Left** | 5HT1A | *z* | 0.617 | 0.570 | 0.574 | 0.038 | 0.043 | 0.066 |
|  |  | *p* | 0.870 | 0.899 | 0.906 | 0.972 | 0.973 | 0.985 |
|  | 5HT1B | *z* | 0.416 | 0.436 | 0.427 | 0.709 | 0.713 | 0.737 |
|  |  | *p* | 0.870 | 0.899 | 0.906 | 0.972 | 0.973 | 0.964 |
|  | 5HT2A | *z* | 0.172 | 0.218 | 0.202 | 0.389 | 0.381 | 0.367 |
|  |  | *p* | 0.960 | 0.963 | 0.964 | 0.972 | 0.973 | 0.985 |
|  | 5HT4 | *z* | 0.541 | 0.483 | 0.497 | 0.066 | 0.070 | 0.064 |
|  |  | *p* | 0.870 | 0.899 | 0.906 | 0.972 | 0.973 | 0.985 |
|  | CB1 | *z* | 0.386 | 0.356 | 0.357 | 0.095 | 0.096 | 0.098 |
|  |  | *p* | 0.870 | 0.899 | 0.906 | 0.972 | 0.973 | 0.985 |
|  | D1 | *z* | 0.107 | 0.084 | 0.094 | 0.226 | 0.240 | 0.261 |
|  |  | *p* | 0.960 | 0.963 | 0.964 | 0.972 | 0.973 | 0.985 |
|  | D2 | *z* | 0.613 | 0.565 | 0.575 | 0.040 | 0.045 | 0.074 |
|  |  | *p* | 0.870 | 0.899 | 0.906 | 0.972 | 0.973 | 0.985 |
|  | DAT | *z* | 0.074 | 0.029 | 0.057 | 0.309 | 0.306 | 0.319 |
|  |  | *p* | 0.960 | 0.963 | 0.964 | 0.972 | 0.973 | 0.985 |
|  | FDOPA | *z* | 0.199 | 0.185 | 0.186 | 0.281 | 0.290 | 0.311 |
|  |  | *p* | 0.960 | 0.963 | 0.964 | 0.972 | 0.973 | 0.985 |
|  | GABAa | *z* | 0.426 | 0.368 | 0.388 | 0.123 | 0.122 | 0.133 |
|  |  | *p* | 0.870 | 0.899 | 0.906 | 0.972 | 0.973 | 0.985 |
|  | KappaOp | *z* | 0.992 | 1.008 | 1.002 | 1.210 | 1.213 | 1.234 |
|  |  | *p* | 0.870 | 0.772 | 0.801 | 0.245 | 0.194 | 0.202 |
|  | mGluR5 | *z* | 0.485 | 0.423 | 0.445 | 0.055 | 0.054 | 0.063 |
|  |  | *p* | 0.870 | 0.899 | 0.906 | 0.972 | 0.973 | 0.985 |
|  | MU | *z* | 0.740 | 0.759 | 0.761 | 1.291 | 1.306 | 1.355 |
|  |  | *p* | 0.870 | 0.807 | 0.801 | 0.203 | 0.194 | 0.178 |
|  | NA | *z* | 0.099 | 0.074 | 0.093 | 0.027 | 0.026 | 0.014 |
|  |  | *p* | 0.960 | 0.963 | 0.964 | 0.972 | 0.973 | 0.985 |
|  | NMDA | *z* | 0.016 | 0.055 | 0.028 | 0.074 | 0.062 | 0.050 |
|  |  | *p* | 0.980 | 0.963 | 0.964 | 0.972 | 0.973 | 0.985 |
|  | SERT_DAS | *z* | 0.801 | 0.863 | 0.825 | 0.816 | 0.797 | 0.783 |
|  |  | *p* | 0.870 | 0.772 | 0.801 | 0.885 | 0.932 | 0.964 |
|  | SERT_MADAM | *z* | 0.804 | 0.860 | 0.835 | 1.338 | 1.326 | 1.352 |
|  |  | *p* | 0.870 | 0.772 | 0.801 | 0.203 | 0.194 | 0.178 |
|  | VACh | *z* | 0.209 | 0.168 | 0.181 | 0.370 | 0.376 | 0.404 |
|  |  | *p* | 0.960 | 0.963 | 0.964 | 0.972 | 0.973 | 0.985 |
| **First-order gradient_Right** | 5HT1A | *z* | 0.446 | 0.381 | 0.408 | 0.738 | 0.875 | 0.849 |
|  |  | *p* | 0.942 | 0.917 | 0.944 | 0.838 | 0.773 | 0.726 |
|  | 5HT1B | *z* | 0.338 | 0.338 | 0.355 | 0.716 | 0.689 | 0.707 |
|  |  | *p* | 0.942 | 0.917 | 0.944 | 0.838 | 0.773 | 0.726 |
|  | 5HT2A | *z* | 0.145 | 0.172 | 0.154 | 0.255 | 0.249 | 0.245 |
|  |  | *p* | 0.942 | 0.917 | 0.944 | 0.842 | 0.874 | 0.863 |
|  | 5HT4 | *z* | 0.192 | 0.122 | 0.160 | 0.669 | 0.754 | 0.730 |
|  |  | *p* | 0.942 | 0.917 | 0.944 | 0.838 | 0.773 | 0.726 |
|  | CB1 | *z* | 0.199 | 0.163 | 0.186 | 0.282 | 0.322 | 0.308 |
|  |  | *p* | 0.942 | 0.917 | 0.944 | 0.842 | 0.874 | 0.863 |
|  | D1 | *z* | 0.047 | 0.001 | 0.044 | 0.240 | 0.231 | 0.236 |
|  |  | *p* | 0.942 | 0.998 | 0.944 | 0.842 | 0.874 | 0.863 |
|  | D2 | *z* | 0.278 | 0.257 | 0.254 | 0.644 | 0.715 | 0.721 |
|  |  | *p* | 0.942 | 0.917 | 0.944 | 0.838 | 0.773 | 0.726 |
|  | DAT | *z* | 0.094 | 0.104 | 0.079 | 0.587 | 0.601 | 0.601 |
|  |  | *p* | 0.942 | 0.917 | 0.944 | 0.838 | 0.814 | 0.822 |
|  | FDOPA | *z* | 0.504 | 0.457 | 0.501 | 0.254 | 0.193 | 0.211 |
|  |  | *p* | 0.942 | 0.917 | 0.944 | 0.842 | 0.874 | 0.863 |
|  | GABAa | *z* | 0.182 | 0.167 | 0.161 | 0.790 | 0.850 | 0.832 |
|  |  | *p* | 0.942 | 0.917 | 0.944 | 0.838 | 0.773 | 0.726 |
|  | KappaOp | *z* | 1.371 | 1.235 | 1.353 | 0.447 | 0.338 | 0.360 |
|  |  | *p* | 0.230 | 0.283 | 0.225 | 0.842 | 0.874 | 0.863 |
|  | mGluR5 | *z* | 0.311 | 0.284 | 0.285 | 0.728 | 0.811 | 0.787 |
|  |  | *p* | 0.942 | 0.917 | 0.944 | 0.838 | 0.773 | 0.726 |
|  | MU | *z* | 1.178 | 1.191 | 1.213 | 0.864 | 0.743 | 0.772 |
|  |  | *p* | 0.364 | 0.283 | 0.305 | 0.838 | 0.773 | 0.726 |
|  | NA | *z* | 0.059 | 0.131 | 0.080 | 0.115 | 0.140 | 0.143 |
|  |  | *p* | 0.942 | 0.917 | 0.944 | 0.913 | 0.874 | 0.872 |
|  | NMDA | *z* | 0.441 | 0.441 | 0.449 | 0.352 | 0.303 | 0.298 |
|  |  | *p* | 0.942 | 0.917 | 0.944 | 0.842 | 0.874 | 0.863 |
|  | SERT_DAS | *z* | 0.940 | 0.967 | 0.966 | 0.565 | 0.478 | 0.497 |
|  |  | *p* | 0.700 | 0.555 | 0.676 | 0.838 | 0.874 | 0.863 |
|  | SERT_MADAM | *z* | 1.356 | 1.343 | 1.378 | 0.503 | 0.401 | 0.425 |
|  |  | *p* | 0.230 | 0.283 | 0.225 | 0.842 | 0.874 | 0.863 |
|  | VACh | *z* | 0.256 | 0.221 | 0.226 | 0.829 | 0.930 | 0.923 |
|  |  | *p* | 0.942 | 0.917 | 0.944 | 0.838 | 0.773 | 0.726 |
| **Second-order gradient_Left** | 5HT1A | *z* | 0.187 | 0.330 | 0.319 | 0.223 | 0.265 | 0.205 |
|  |  | *p* | 0.871 | 0.840 | 0.831 | 0.746 | 0.743 | 0.718 |
|  | 5HT1B | *z* | 0.446 | 0.506 | 0.479 | 0.389 | 0.405 | 0.318 |
|  |  | *p* | 0.871 | 0.840 | 0.831 | 0.737 | 0.741 | 0.714 |
|  | 5HT2A | *z* | 0.612 | 0.511 | 0.545 | 0.821 | 0.791 | 0.876 |
|  |  | *p* | 0.871 | 0.840 | 0.831 | 0.495 | 0.594 | 0.416 |
|  | 5HT4 | *z* | 0.465 | 0.320 | 0.335 | 0.447 | 0.406 | 0.451 |
|  |  | *p* | 0.871 | 0.840 | 0.831 | 0.737 | 0.741 | 0.714 |
|  | CB1 | *z* | 0.403 | 0.326 | 0.327 | 0.380 | 0.356 | 0.359 |
|  |  | *p* | 0.871 | 0.840 | 0.831 | 0.737 | 0.741 | 0.714 |
|  | D1 | *z* | 1.948 ** | 1.799 | 1.813 * | 1.586 * | 1.568 | 1.417 |
|  |  | *p* | 0.007 | 0.059 | 0.049 | 0.049 | 0.081 | 0.063 |
|  | D2 | *z* | 0.282 | 0.441 | 0.426 | 0.320 | 0.367 | 0.290 |
|  |  | *p* | 0.871 | 0.840 | 0.831 | 0.737 | 0.741 | 0.714 |
|  | DAT | *z* | 0.054 | 0.184 | 0.142 | 0.153 | 0.128 | 0.227 |
|  |  | *p* | 0.936 | 0.935 | 0.922 | 0.820 | 0.859 | 0.714 |
|  | FDOPA | *z* | 1.061 | 1.053 | 1.033 | 0.875 | 0.872 | 0.794 |
|  |  | *p* | 0.604 | 0.690 | 0.716 | 0.495 | 0.529 | 0.484 |
|  | GABAa | *z* | 0.147 | 0.002 | 0.026 | 0.256 | 0.219 | 0.294 |
|  |  | *p* | 0.871 | 0.998 | 0.970 | 0.737 | 0.757 | 0.714 |
|  | KappaOp | *z* | 0.404 | 0.401 | 0.384 | 0.293 | 0.302 | 0.230 |
|  |  | *p* | 0.871 | 0.840 | 0.831 | 0.737 | 0.741 | 0.714 |
|  | mGluR5 | *z* | 0.231 | 0.078 | 0.105 | 0.328 | 0.290 | 0.362 |
|  |  | *p* | 0.871 | 0.968 | 0.922 | 0.737 | 0.741 | 0.714 |
|  | MU | *z* | 0.518 | 0.546 | 0.530 | 0.440 | 0.462 | 0.373 |
|  |  | *p* | 0.871 | 0.840 | 0.831 | 0.737 | 0.741 | 0.714 |
|  | NA | *z* | 0.174 | 0.112 | 0.141 | 0.455 | 0.457 | 0.491 |
|  |  | *p* | 0.871 | 0.968 | 0.922 | 0.737 | 0.741 | 0.714 |
|  | NMDA | *z* | 0.806 | 0.692 | 0.738 | 1.211 | 1.177 | 1.296 |
|  |  | *p* | 0.871 | 0.840 | 0.831 | 0.204 | 0.202 | 0.082 |
|  | SERT_DAS | *z* | 0.378 | 0.342 | 0.374 | 0.537 | 0.523 | 0.613 |
|  |  | *p* | 0.871 | 0.840 | 0.831 | 0.737 | 0.741 | 0.714 |
|  | SERT_MADAM | *z* | 0.138 | 0.190 | 0.162 | 0.027 | 0.047 | 0.045 |
|  |  | *p* | 0.871 | 0.935 | 0.922 | 0.960 | 0.927 | 0.928 |
|  | VACh | *z* | 0.368 | 0.538 | 0.503 | 0.303 | 0.344 | 0.240 |
|  |  | *p* | 0.871 | 0.840 | 0.831 | 0.737 | 0.741 | 0.714 |
| **Second-order gradient_Right** | 5HT1A | *z* | 0.468 | 0.462 | 0.383 | 1.146 | 0.917 | 1.469 |
|  |  | *p* | 0.769 | 0.790 | 0.772 | 0.563 | 0.624 | 0.464 |
|  | 5HT1B | *z* | 0.388 | 0.407 | 0.398 | 0.145 | 0.846 | 0.008 |
|  |  | *p* | 0.769 | 0.790 | 0.772 | 0.906 | 0.624 | 0.993 |
|  | 5HT2A | *z* | 0.508 | 0.452 | 0.504 | 0.531 | 0.193 | 0.357 |
|  |  | *p* | 0.769 | 0.790 | 0.772 | 0.760 | 0.941 | 0.884 |
|  | 5HT4 | *z* | 0.024 | 0.009 | 0.079 | 1.354 | 0.366 | 1.205 |
|  |  | *p* | 0.983 | 0.995 | 0.926 | 0.563 | 0.829 | 0.464 |
|  | CB1 | *z* | 0.011 | 0.004 | 0.044 | 0.815 | 0.022 | 0.685 |
|  |  | *p* | 0.983 | 0.995 | 0.926 | 0.760 | 0.973 | 0.805 |
|  | D1 | *z* | 1.165 | 1.125 | 1.196 | 0.428 | 0.531 | 0.218 |
|  |  | *p* | 0.356 | 0.383 | 0.320 | 0.760 | 0.806 | 0.949 |
|  | D2 | *z* | 0.542 | 0.543 | 0.491 | 0.437 | 1.579 | 0.643 |
|  |  | *p* | 0.769 | 0.790 | 0.772 | 0.760 | 0.134 | 0.805 |
|  | DAT | *z* | 0.262 | 0.298 | 0.253 | 0.445 | 0.594 | 0.510 |
|  |  | *p* | 0.770 | 0.790 | 0.772 | 0.760 | 0.806 | 0.805 |
|  | FDOPA | *z* | 0.320 | 0.333 | 0.370 | 0.655 | 0.278 | 0.521 |
|  |  | *p* | 0.769 | 0.790 | 0.772 | 0.760 | 0.829 | 0.805 |
|  | GABAa | *z* | 0.310 | 0.348 | 0.279 | 0.973 | 0.485 | 0.986 |
|  |  | *p* | 0.769 | 0.790 | 0.772 | 0.760 | 0.806 | 0.724 |
|  | KappaOp | *z* | 0.161 | 0.197 | 0.236 | 0.812 | 0.018 | 0.777 |
|  |  | *p* | 0.884 | 0.880 | 0.772 | 0.760 | 0.973 | 0.805 |
|  | mGluR5 | *z* | 0.317 | 0.345 | 0.270 | 1.195 | 0.532 | 1.245 |
|  |  | *p* | 0.769 | 0.790 | 0.772 | 0.563 | 0.806 | 0.464 |
|  | MU | *z* | 0.260 | 0.291 | 0.307 | 0.430 | 0.314 | 0.376 |
|  |  | *p* | 0.770 | 0.790 | 0.772 | 0.760 | 0.829 | 0.884 |
|  | NA | *z* | 0.031 | 0.075 | 0.069 | 0.097 | 0.379 | 0.020 |
|  |  | *p* | 0.983 | 0.990 | 0.926 | 0.906 | 0.829 | 0.993 |
|  | NMDA | *z* | 0.356 | 0.299 | 0.321 | 0.217 | 0.315 | 0.065 |
|  |  | *p* | 0.769 | 0.790 | 0.772 | 0.906 | 0.829 | 0.993 |
|  | SERT_DAS | *z* | 0.315 | 0.265 | 0.266 | 0.180 | 0.086 | 0.236 |
|  |  | *p* | 0.769 | 0.793 | 0.772 | 0.906 | 0.973 | 0.949 |
|  | SERT_MADAM | *z* | 0.155 | 0.115 | 0.094 | 0.532 | 0.076 | 0.551 |
|  |  | *p* | 0.884 | 0.983 | 0.926 | 0.760 | 0.973 | 0.805 |
|  | VACh | *z* | 0.489 | 0.495 | 0.430 | 0.629 | 1.435 | 0.843 |
|  |  | *p* | 0.769 | 0.790 | 0.772 | 0.760 | 0.134 | 0.805 |
| **Third-order gradient_Left** | 5HT1A | *z* | 1.444 | 1.545 | 1.543 | 1.955 * | 1.955 ** | 1.948 * |
|  |  | *p* | 0.148 | 0.124 | 0.130 | 0.018 | 0.009 | 0.020 |
|  | 5HT1B | *z* | 0.064 | 0.045 | 0.058 | 0.039 | 0.037 | 0.044 |
|  |  | *p* | 0.929 | 0.999 | 0.996 | 0.965 | 0.963 | 0.970 |
|  | 5HT2A | *z* | 0.138 | 0.223 | 0.217 | 0.224 | 0.228 | 0.217 |
|  |  | *p* | 0.929 | 0.999 | 0.996 | 0.965 | 0.963 | 0.970 |
|  | 5HT4 | *z* | 1.015 | 1.168 | 1.161 | 1.121 | 1.127 | 1.107 |
|  |  | *p* | 0.542 | 0.364 | 0.392 | 0.324 | 0.332 | 0.340 |
|  | CB1 | *z* | 0.691 | 0.727 | 0.737 | 0.646 | 0.649 | 0.634 |
|  |  | *p* | 0.824 | 0.780 | 0.816 | 0.869 | 0.852 | 0.874 |
|  | D1 | *z* | 0.114 | 0.161 | 0.159 | 0.035 | 0.037 | 0.028 |
|  |  | *p* | 0.929 | 0.999 | 0.996 | 0.965 | 0.963 | 0.970 |
|  | D2 | *z* | 1.206 | 1.290 | 1.270 | 1.708 * | 1.703 * | 1.732 * |
|  |  | *p* | 0.335 | 0.326 | 0.363 | 0.036 | 0.039 | 0.050 |
|  | DAT | *z* | 0.123 | 0.231 | 0.213 | 0.434 | 0.436 | 0.439 |
|  |  | *p* | 0.929 | 0.999 | 0.996 | 0.965 | 0.963 | 0.970 |
|  | FDOPA | *z* | 0.251 | 0.268 | 0.273 | 0.121 | 0.123 | 0.113 |
|  |  | *p* | 0.929 | 0.999 | 0.996 | 0.965 | 0.963 | 0.970 |
|  | GABAa | *z* | 0.763 | 0.914 | 0.901 | 1.135 | 1.143 | 1.129 |
|  |  | *p* | 0.790 | 0.551 | 0.577 | 0.324 | 0.332 | 0.340 |
|  | KappaOp | *z* | 0.376 | 0.369 | 0.380 | 0.301 | 0.301 | 0.299 |
|  |  | *p* | 0.929 | 0.999 | 0.996 | 0.965 | 0.963 | 0.970 |
|  | mGluR5 | *z* | 0.793 | 0.957 | 0.942 | 1.162 | 1.170 | 1.156 |
|  |  | *p* | 0.790 | 0.551 | 0.577 | 0.324 | 0.332 | 0.340 |
|  | MU | *z* | 0.086 | 0.092 | 0.099 | 0.040 | 0.040 | 0.039 |
|  |  | *p* | 0.929 | 0.999 | 0.996 | 0.965 | 0.963 | 0.970 |
|  | NA | *z* | 0.093 | 0.001 | 0.005 | 0.137 | 0.141 | 0.136 |
|  |  | *p* | 0.929 | 0.999 | 0.996 | 0.965 | 0.963 | 0.970 |
|  | NMDA | *z* | 0.078 | 0.030 | 0.016 | 0.082 | 0.086 | 0.079 |
|  |  | *p* | 0.929 | 0.999 | 0.996 | 0.965 | 0.963 | 0.970 |
|  | SERT_DAS | *z* | 0.386 | 0.301 | 0.321 | 0.275 | 0.272 | 0.276 |
|  |  | *p* | 0.929 | 0.999 | 0.996 | 0.965 | 0.963 | 0.970 |
|  | SERT_MADAM | *z* | 0.187 | 0.139 | 0.156 | 0.071 | 0.070 | 0.069 |
|  |  | *p* | 0.929 | 0.999 | 0.996 | 0.965 | 0.963 | 0.970 |
|  | VACh | *z* | 0.621 | 0.692 | 0.676 | 0.917 | 0.916 | 0.925 |
|  |  | *p* | 0.831 | 0.780 | 0.816 | 0.527 | 0.538 | 0.525 |
| **Third-order gradient_Right** | 5HT1A | *z* | 1.514 | 1.558 | 1.615 * | 1.838 ** | 1.703 | 1.507 |
|  |  | *p* | 0.113 | 0.088 | 0.032 | 0.004 | 0.175 | 0.079 |
|  | 5HT1B | *z* | 0.043 | 0.060 | 0.072 | 0.247 | 0.080 | 0.403 |
|  |  | *p* | 0.959 | 0.941 | 0.946 | 0.882 | 0.996 | 0.866 |
|  | 5HT2A | *z* | 0.154 | 0.153 | 0.123 | 0.034 | 0.258 | 0.112 |
|  |  | *p* | 0.944 | 0.941 | 0.946 | 0.967 | 0.973 | 0.930 |
|  | 5HT4 | *z* | 1.044 | 1.040 | 1.012 | 0.823 | 1.071 | 0.607 |
|  |  | *p* | 0.760 | 0.769 | 0.805 | 0.581 | 0.572 | 0.866 |
|  | CB1 | *z* | 0.571 | 0.559 | 0.548 | 0.416 | 0.623 | 0.253 |
|  |  | *p* | 0.885 | 0.881 | 0.932 | 0.882 | 0.817 | 0.866 |
|  | D1 | *z* | 0.114 | 0.100 | 0.061 | 0.165 | 0.057 | 0.367 |
|  |  | *p* | 0.944 | 0.941 | 0.946 | 0.882 | 0.996 | 0.866 |
|  | D2 | *z* | 0.625 | 0.657 | 0.675 | 1.020 | 0.784 | 1.250 |
|  |  | *p* | 0.885 | 0.842 | 0.807 | 0.403 | 0.817 | 0.134 |
|  | DAT | *z* | 0.257 | 0.288 | 0.289 | 0.542 | 0.582 | 0.574 |
|  |  | *p* | 0.944 | 0.941 | 0.946 | 0.882 | 0.817 | 0.866 |
|  | FDOPA | *z* | 0.445 | 0.435 | 0.413 | 0.228 | 0.409 | 0.055 |
|  |  | *p* | 0.935 | 0.941 | 0.946 | 0.882 | 0.949 | 0.930 |
|  | GABAa | *z* | 0.628 | 0.652 | 0.652 | 0.828 | 1.063 | 0.716 |
|  |  | *p* | 0.885 | 0.842 | 0.807 | 0.581 | 0.572 | 0.861 |
|  | KappaOp | *z* | 0.670 | 0.676 | 0.658 | 0.539 | 0.674 | 0.380 |
|  |  | *p* | 0.885 | 0.842 | 0.807 | 0.882 | 0.817 | 0.866 |
|  | mGluR5 | *z* | 0.808 | 0.838 | 0.839 | 1.026 | 1.341 | 0.861 |
|  |  | *p* | 0.885 | 0.842 | 0.805 | 0.403 | 0.410 | 0.576 |
|  | MU | *z* | 0.206 | 0.216 | 0.202 | 0.174 | 0.305 | 0.063 |
|  |  | *p* | 0.944 | 0.941 | 0.946 | 0.882 | 0.973 | 0.930 |
|  | NA | *z* | 0.334 | 0.313 | 0.319 | 0.156 | 0.039 | 0.204 |
|  |  | *p* | 0.944 | 0.941 | 0.946 | 0.882 | 0.996 | 0.893 |
|  | NMDA | *z* | 0.147 | 0.146 | 0.164 | 0.188 | 0.005 | 0.293 |
|  |  | *p* | 0.944 | 0.941 | 0.946 | 0.882 | 0.996 | 0.866 |
|  | SERT_DAS | *z* | 0.273 | 0.274 | 0.289 | 0.279 | 0.262 | 0.251 |
|  |  | *p* | 0.944 | 0.941 | 0.946 | 0.882 | 0.973 | 0.866 |
|  | SERT_MADAM | *z* | 0.480 | 0.485 | 0.490 | 0.460 | 0.539 | 0.358 |
|  |  | *p* | 0.935 | 0.941 | 0.932 | 0.882 | 0.817 | 0.866 |
|  | VACh | *z* | 0.797 | 0.834 | 0.852 | 1.213 | 0.987 | 1.370 |
|  |  | *p* | 0.885 | 0.842 | 0.805 | 0.395 | 0.593 | 0.101 |
| **Fourth-order gradient_Left** | 5HT1A | *z* | 0.613 | 0.361 | 0.314 | 0.071 | 0.065 | 0.091 |
|  |  | *p* | 0.541 | 0.733 | 0.785 | 0.959 | 0.966 | 0.839 |
|  | 5HT1B | *z* | 0.278 | 0.271 | 0.310 | 0.436 | 0.428 | 0.466 |
|  |  | *p* | 0.833 | 0.847 | 0.785 | 0.827 | 0.843 | 0.839 |
|  | 5HT2A | *z* | 0.609 | 0.633 | 0.581 | 0.300 | 0.309 | 0.214 |
|  |  | *p* | 0.541 | 0.512 | 0.583 | 0.827 | 0.843 | 0.784 |
|  | 5HT4 | *z* | 0.598 | 0.439 | 0.359 | 0.113 | 0.101 | 0.183 |
|  |  | *p* | 0.541 | 0.699 | 0.785 | 0.959 | 0.966 | 0.951 |
|  | CB1 | *z* | 0.290 | 0.179 | 0.117 | 0.243 | 0.233 | 0.309 |
|  |  | *p* | 0.833 | 0.887 | 0.903 | 0.827 | 0.843 | 0.951 |
|  | D1 | *z* | 0.018 | 0.037 | 0.021 | 0.270 | 0.259 | 0.354 |
|  |  | *p* | 0.978 | 0.953 | 0.974 | 0.827 | 0.843 | 0.951 |
|  | D2 | *z* | 0.640 | 0.400 | 0.363 | 0.018 | 0.023 | 0.012 |
|  |  | *p* | 0.541 | 0.699 | 0.785 | 0.976 | 0.971 | 0.956 |
|  | DAT | *z* | 1.459 | 1.459 * | 1.456 * | 0.953 | 0.962 | 0.901 |
|  |  | *p* | 0.122 | 0.034 | 0.018 | 0.560 | 0.475 | 0.677 |
|  | FDOPA | *z* | 0.118 | 0.117 | 0.175 | 0.452 | 0.442 | 0.533 |
|  |  | *p* | 0.906 | 0.887 | 0.863 | 0.827 | 0.843 | 0.839 |
|  | GABAa | *z* | 1.191 | 0.908 | 0.818 | 0.286 | 0.297 | 0.229 |
|  |  | *p* | 0.253 | 0.314 | 0.416 | 0.827 | 0.843 | 0.839 |
|  | KappaOp | *z* | 0.114 | 0.165 | 0.209 | 0.493 | 0.491 | 0.508 |
|  |  | *p* | 0.906 | 0.887 | 0.863 | 0.827 | 0.843 | 0.677 |
|  | mGluR5 | *z* | 1.128 | 0.875 | 0.781 | 0.240 | 0.251 | 0.181 |
|  |  | *p* | 0.253 | 0.314 | 0.416 | 0.827 | 0.843 | 0.951 |
|  | MU | *z* | 0.169 | 0.149 | 0.180 | 0.352 | 0.351 | 0.363 |
|  |  | *p* | 0.906 | 0.887 | 0.863 | 0.827 | 0.843 | 0.839 |
|  | NA | *z* | 0.851 | 0.945 | 0.929 | 0.686 | 0.691 | 0.633 |
|  |  | *p* | 0.505 | 0.314 | 0.401 | 0.827 | 0.843 | 0.562 |
|  | NMDA | *z* | 0.605 | 0.752 | 0.698 | 0.444 | 0.458 | 0.361 |
|  |  | *p* | 0.541 | 0.422 | 0.491 | 0.827 | 0.843 | 0.562 |
|  | SERT_DAS | *z* | 0.317 | 0.463 | 0.472 | 0.558 | 0.564 | 0.499 |
|  |  | *p* | 0.833 | 0.699 | 0.740 | 0.827 | 0.843 | 0.562 |
|  | SERT_MADAM | *z* | 0.378 | 0.430 | 0.459 | 0.591 | 0.591 | 0.573 |
|  |  | *p* | 0.833 | 0.699 | 0.740 | 0.827 | 0.843 | 0.599 |
|  | VACh | *z* | 0.881 | 0.661 | 0.651 | 0.369 | 0.370 | 0.366 |
|  |  | *p* | 0.505 | 0.509 | 0.528 | 0.827 | 0.843 | 0.956 |
| **Fourth-order gradient_Right** | 5HT1A | *z* | 0.420 | 0.281 | 0.391 | 0.196 | 0.274 | 0.333 |
|  |  | *p* | 0.709 | 0.764 | 0.694 | 0.939 | 0.978 | 0.839 |
|  | 5HT1B | *z* | 0.322 | 0.328 | 0.297 | 0.384 | 0.227 | 0.335 |
|  |  | *p* | 0.751 | 0.745 | 0.771 | 0.860 | 0.978 | 0.839 |
|  | 5HT2A | *z* | 0.819 | 0.841 | 0.886 | 0.532 | 0.448 | 0.485 |
|  |  | *p* | 0.551 | 0.356 | 0.468 | 0.692 | 0.978 | 0.784 |
|  | 5HT4 | *z* | 0.623 | 0.508 | 0.630 | 0.015 | 0.053 | 0.096 |
|  |  | *p* | 0.645 | 0.730 | 0.647 | 0.979 | 0.978 | 0.951 |
|  | CB1 | *z* | 0.384 | 0.316 | 0.403 | 0.072 | 0.053 | 0.148 |
|  |  | *p* | 0.710 | 0.745 | 0.694 | 0.939 | 0.978 | 0.951 |
|  | D1 | *z* | 0.198 | 0.212 | 0.242 | 0.085 | 0.010 | 0.098 |
|  |  | *p* | 0.852 | 0.786 | 0.771 | 0.939 | 0.984 | 0.951 |
|  | D2 | *z* | 0.483 | 0.389 | 0.439 | 0.137 | 0.075 | 0.039 |
|  |  | *p* | 0.670 | 0.745 | 0.694 | 0.939 | 0.978 | 0.956 |
|  | DAT | *z* | 1.176 | 1.086 | 1.111 | 0.740 | 0.354 | 0.622 |
|  |  | *p* | 0.243 | 0.356 | 0.371 | 0.497 | 0.978 | 0.677 |
|  | FDOPA | *z* | 0.005 | 0.051 | 0.023 | 0.352 | 0.332 | 0.368 |
|  |  | *p* | 0.992 | 0.924 | 0.964 | 0.860 | 0.978 | 0.839 |
|  | GABAa | *z* | 1.271 | 1.055 | 1.236 | 0.421 | 0.303 | 0.288 |
|  |  | *p* | 0.243 | 0.356 | 0.356 | 0.860 | 0.978 | 0.839 |
|  | KappaOp | *z* | 0.095 | 0.191 | 0.103 | 0.549 | 0.797 | 0.570 |
|  |  | *p* | 0.911 | 0.786 | 0.888 | 0.692 | 0.700 | 0.677 |
|  | mGluR5 | *z* | 1.047 | 0.853 | 1.012 | 0.271 | 0.137 | 0.146 |
|  |  | *p* | 0.339 | 0.356 | 0.371 | 0.901 | 0.978 | 0.951 |
|  | MU | *z* | 0.120 | 0.172 | 0.127 | 0.315 | 0.563 | 0.280 |
|  |  | *p* | 0.911 | 0.786 | 0.888 | 0.864 | 0.906 | 0.839 |
|  | NA | *z* | 0.719 | 0.772 | 0.737 | 0.745 | 0.724 | 0.757 |
|  |  | *p* | 0.566 | 0.400 | 0.550 | 0.497 | 0.700 | 0.562 |
|  | NMDA | *z* | 0.781 | 0.862 | 0.824 | 0.802 | 0.687 | 0.765 |
|  |  | *p* | 0.551 | 0.356 | 0.498 | 0.497 | 0.700 | 0.562 |
|  | SERT_DAS | *z* | 0.532 | 0.635 | 0.558 | 0.874 | 0.702 | 0.851 |
|  |  | *p* | 0.667 | 0.552 | 0.659 | 0.497 | 0.700 | 0.562 |
|  | SERT_MADAM | *z* | 0.238 | 0.340 | 0.256 | 0.674 | 0.700 | 0.689 |
|  |  | *p* | 0.847 | 0.745 | 0.771 | 0.528 | 0.700 | 0.599 |
|  | VACh | *z* | 0.585 | 0.470 | 0.539 | 0.139 | 0.090 | 0.028 |
|  |  | *p* | 0.645 | 0.732 | 0.659 | 0.939 | 0.978 | 0.956 |
| **Fifth-order gradient_Left** | 5HT1A | *z* | 0.159 | 0.179 | 0.151 | 0.228 | 0.123 | 0.606 |
|  |  | *p* | 0.934 | 0.916 | 0.944 | 0.851 | 0.993 | 0.796 |
|  | 5HT1B | *z* | 0.846 | 0.771 | 0.826 | 0.820 | 0.507 | 0.187 |
|  |  | *p* | 0.130 | 0.205 | 0.151 | 0.430 | 0.551 | 0.859 |
|  | 5HT2A | *z* | 0.422 | 0.513 | 0.586 | 0.116 | 0.266 | 0.340 |
|  |  | *p* | 0.724 | 0.723 | 0.511 | 0.972 | 0.745 | 0.796 |
|  | 5HT4 | *z* | 0.370 | 0.404 | 0.440 | 0.254 | 0.069 | 0.465 |
|  |  | *p* | 0.748 | 0.893 | 0.742 | 0.851 | 0.993 | 0.796 |
|  | CB1 | *z* | 0.842 | 0.840 | 0.864 | 0.573 | 0.053 | 0.790 |
|  |  | *p* | 0.130 | 0.205 | 0.151 | 0.560 | 0.993 | 0.580 |
|  | D1 | *z* | 0.298 | 0.294 | 0.379 | 0.025 | 0.020 | 0.030 |
|  |  | *p* | 0.934 | 0.893 | 0.797 | 0.973 | 0.993 | 0.993 |
|  | D2 | *z* | 0.166 | 0.136 | 0.159 | 0.017 | 0.072 | 0.262 |
|  |  | *p* | 0.934 | 0.916 | 0.944 | 0.973 | 0.993 | 0.796 |
|  | DAT | *z* | 0.415 | 0.263 | 0.192 | 0.250 | 0.044 | 0.324 |
|  |  | *p* | 0.724 | 0.908 | 0.944 | 0.851 | 0.993 | 0.796 |
|  | FDOPA | *z* | 0.512 | 0.475 | 0.525 | 0.243 | 0.004 | 0.237 |
|  |  | *p* | 0.724 | 0.723 | 0.587 | 0.851 | 0.993 | 0.796 |
|  | GABAa | *z* | 0.208 | 0.299 | 0.349 | 0.222 | 0.027 | 0.343 |
|  |  | *p* | 0.934 | 0.893 | 0.797 | 0.851 | 0.993 | 0.796 |
|  | KappaOp | *z* | 0.180 | 0.139 | 0.182 | 0.399 | 0.386 | 0.003 |
|  |  | *p* | 0.934 | 0.916 | 0.944 | 0.851 | 0.745 | 0.993 |
|  | mGluR5 | *z* | 0.166 | 0.252 | 0.310 | 0.186 | 0.003 | 0.272 |
|  |  | *p* | 0.934 | 0.908 | 0.797 | 0.879 | 0.993 | 0.796 |
|  | MU | *z* | 0.040 | 0.015 | 0.082 | 0.264 | 0.499 | 0.295 |
|  |  | *p* | 0.974 | 0.991 | 0.959 | 0.851 | 0.551 | 0.796 |
|  | NA | *z* | 0.239 | 0.375 | 0.500 | 0.367 | 0.321 | 0.077 |
|  |  | *p* | 0.934 | 0.893 | 0.587 | 0.851 | 0.745 | 0.945 |
|  | NMDA | *z* | 0.069 | 0.174 | 0.312 | 0.055 | 0.260 | 0.380 |
|  |  | *p* | 0.974 | 0.916 | 0.797 | 0.973 | 0.745 | 0.796 |
|  | SERT_DAS | *z* | 0.293 | 0.209 | 0.156 | 0.665 | 0.308 | 0.358 |
|  |  | *p* | 0.934 | 0.916 | 0.944 | 0.430 | 0.745 | 0.796 |
|  | SERT_MADAM | *z* | 0.407 | 0.327 | 0.334 | 0.696 | 0.489 | 0.126 |
|  |  | *p* | 0.724 | 0.893 | 0.797 | 0.430 | 0.551 | 0.906 |
|  | VACh | *z* | 0.399 | 0.320 | 0.332 | 0.259 | 0.258 | 0.120 |
|  |  | *p* | 0.724 | 0.893 | 0.797 | 0.851 | 0.745 | 0.906 |
| **Fifth-order gradient_Right** | 5HT1A | *z* | 0.107 | 0.053 | 0.044 | 0.577 | 0.592 | 0.542 |
|  |  | *p* | 0.957 | 0.951 | 0.953 | 0.916 | 0.593 | 0.946 |
|  | 5HT1B | *z* | 1.134 | 0.984 | 1.079 | 0.066 | 0.223 | 0.032 |
|  |  | *p* | 0.207 | 0.198 | 0.238 | 0.916 | 0.872 | 0.946 |
|  | 5HT2A | *z* | 0.351 | 0.442 | 0.299 | 0.225 | 0.291 | 0.171 |
|  |  | *p* | 0.886 | 0.726 | 0.901 | 0.916 | 0.767 | 0.946 |
|  | 5HT4 | *z* | 0.223 | 0.403 | 0.249 | 0.602 | 0.333 | 0.522 |
|  |  | *p* | 0.886 | 0.761 | 0.901 | 0.916 | 0.767 | 0.946 |
|  | CB1 | *z* | 0.798 | 0.993 | 0.832 | 0.692 | 0.438 | 0.691 |
|  |  | *p* | 0.257 | 0.198 | 0.248 | 0.916 | 0.652 | 0.946 |
|  | D1 | *z* | 0.626 | 0.681 | 0.537 | 0.049 | 0.170 | 0.126 |
|  |  | *p* | 0.385 | 0.349 | 0.482 | 0.916 | 0.935 | 0.946 |
|  | D2 | *z* | 0.868 | 0.674 | 0.805 | 0.156 | 0.071 | 0.122 |
|  |  | *p* | 0.257 | 0.349 | 0.248 | 0.916 | 0.936 | 0.946 |
|  | DAT | *z* | 0.800 | 0.674 | 0.806 | 0.064 | 0.477 | 0.086 |
|  |  | *p* | 0.257 | 0.349 | 0.248 | 0.916 | 0.619 | 0.946 |
|  | FDOPA | *z* | 0.679 | 0.747 | 0.661 | 0.075 | 0.325 | 0.066 |
|  |  | *p* | 0.343 | 0.349 | 0.329 | 0.916 | 0.767 | 0.946 |
|  | GABAa | *z* | 0.047 | 0.075 | 0.025 | 0.453 | 0.011 | 0.441 |
|  |  | *p* | 0.957 | 0.951 | 0.953 | 0.916 | 0.979 | 0.946 |
|  | KappaOp | *z* | 0.091 | 0.136 | 0.088 | 0.281 | 0.310 | 0.283 |
|  |  | *p* | 0.957 | 0.951 | 0.953 | 0.916 | 0.767 | 0.946 |
|  | mGluR5 | *z* | 0.072 | 0.060 | 0.043 | 0.407 | 0.103 | 0.393 |
|  |  | *p* | 0.957 | 0.951 | 0.953 | 0.916 | 0.936 | 0.946 |
|  | MU | *z* | 0.004 | 0.037 | 0.035 | 0.755 | 0.059 | 0.712 |
|  |  | *p* | 0.992 | 0.951 | 0.953 | 0.916 | 0.936 | 0.946 |
|  | NA | *z* | 0.164 | 0.106 | 0.120 | 0.161 | 0.589 | 0.086 |
|  |  | *p* | 0.954 | 0.951 | 0.953 | 0.916 | 0.593 | 0.946 |
|  | NMDA | *z* | 0.242 | 0.263 | 0.206 | 0.090 | 0.556 | 0.052 |
|  |  | *p* | 0.886 | 0.951 | 0.936 | 0.916 | 0.593 | 0.946 |
|  | SERT_DAS | *z* | 0.233 | 0.195 | 0.271 | 0.115 | 0.634 | 0.029 |
|  |  | *p* | 0.886 | 0.951 | 0.901 | 0.916 | 0.593 | 0.946 |
|  | SERT_MADAM | *z* | 0.227 | 0.226 | 0.251 | 0.171 | 0.485 | 0.110 |
|  |  | *p* | 0.886 | 0.951 | 0.901 | 0.916 | 0.619 | 0.946 |
|  | VACh | *z* | 0.787 | 0.576 | 0.718 | 0.215 | 0.102 | 0.172 |
|  |  | *p* | 0.257 | 0.482 | 0.292 | 0.916 | 0.936 | 0.946 |

The trend-surface regression model with nine coefficients was applied to group-level gradient maps and the neurotransmitter PET/SPECT scans. Fisher’s r-to-z–transformed correlation coefficients were computed between the TSM coefficients representing gradient maps and those representing neurotransmitter maps. The p-value was estimated using a permutation test as the proportion of permuted z-values that were lower than the observed Fisher r-to-z–transformed correlation coefficients, with FDR correction applied. The correction was applied across 18 neurotransmitters within each gradient, and separately for each hemisphere, resting-state and dataset.**p*<0.05, ***p*<0.01.

**Table S9**. Correlations coefficients (Pearson r, absolute values, with parametric testing and FDR correction) between gradient and neurotransmitter layouts

|  |  |  | **HBS**  **Resting-State1** | **HBS**  **Resting-State2** | **HBS**  **Resting-State3** | **MIND-Set**  **Resting-State1** | **MIND-Set**  **Resting-State2** | **MIND-Set**  **Resting-State3** |
| --- | --- | --- | --- | --- | --- | --- | --- | --- |
| **Zeroth-order gradient_Left** | 5HT1A | *r* | 0.781 | 0.775 | 0.778 | 0.718 | 0.714 | 0.717 |
|  |  | *p* | 0.233 | 0.254 | 0.243 | 0.259 | 0.264 | 0.262 |
|  | 5HT1B | *r* | 0.301 | 0.307 | 0.306 | 0.353 | 0.357 | 0.356 |
|  |  | *p* | 0.597 | 0.583 | 0.585 | 0.527 | 0.518 | 0.522 |
|  | 5HT2A | *r* | 0.057 | 0.055 | 0.061 | 0.018 | 0.022 | 0.026 |
|  |  | *p* | 0.936 | 0.941 | 0.927 | 0.963 | 0.956 | 0.948 |
|  | 5HT4 | *r* | 0.705 | 0.701 | 0.706 | 0.664 | 0.665 | 0.667 |
|  |  | *p* | 0.267 | 0.261 | 0.260 | 0.259 | 0.264 | 0.262 |
|  | CB1 | *r* | 0.621 | 0.621 | 0.624 | 0.602 | 0.603 | 0.606 |
|  |  | *p* | 0.267 | 0.261 | 0.260 | 0.259 | 0.264 | 0.262 |
|  | D1 | *r* | 0.208 | 0.211 | 0.215 | 0.239 | 0.246 | 0.246 |
|  |  | *p* | 0.760 | 0.752 | 0.744 | 0.690 | 0.673 | 0.672 |
|  | D2 | *r* | 0.671 | 0.663 | 0.666 | 0.604 | 0.599 | 0.600 |
|  |  | *p* | 0.267 | 0.261 | 0.260 | 0.259 | 0.264 | 0.262 |
|  | DAT | *r* | 0.100 | 0.111 | 0.103 | 0.177 | 0.178 | 0.178 |
|  |  | *p* | 0.898 | 0.873 | 0.890 | 0.732 | 0.743 | 0.744 |
|  | FDOPA | *r* | 0.429 | 0.435 | 0.436 | 0.465 | 0.472 | 0.473 |
|  |  | *p* | 0.448 | 0.436 | 0.433 | 0.414 | 0.399 | 0.397 |
|  | GABAa | *r* | 0.514 | 0.506 | 0.513 | 0.443 | 0.442 | 0.444 |
|  |  | *p* | 0.353 | 0.370 | 0.355 | 0.416 | 0.405 | 0.414 |
|  | KappaOp | *r* | 0.622 | 0.625 | 0.625 | 0.669 | 0.673 | 0.670 |
|  |  | *p* | 0.267 | 0.261 | 0.260 | 0.259 | 0.264 | 0.262 |
|  | mGluR5 | *r* | 0.553 | 0.545 | 0.552 | 0.487 | 0.487 | 0.489 |
|  |  | *p* | 0.316 | 0.332 | 0.317 | 0.413 | 0.399 | 0.397 |
|  | MU | *r* | 0.365 | 0.368 | 0.369 | 0.425 | 0.431 | 0.426 |
|  |  | *p* | 0.547 | 0.539 | 0.538 | 0.416 | 0.405 | 0.414 |
|  | NA | *r* | 0.009 | 0.013 | 0.008 | 0.043 | 0.040 | 0.039 |
|  |  | *p* | 0.981 | 0.973 | 0.984 | 0.963 | 0.956 | 0.948 |
|  | NMDA | *r* | 0.151 | 0.155 | 0.148 | 0.176 | 0.171 | 0.170 |
|  |  | *p* | 0.839 | 0.828 | 0.845 | 0.732 | 0.743 | 0.744 |
|  | SERT_DAS | *r* | 0.597 | 0.601 | 0.596 | 0.632 | 0.630 | 0.627 |
|  |  | *p* | 0.269 | 0.261 | 0.271 | 0.259 | 0.264 | 0.262 |
|  | SERT_MADAM | *r* | 0.467 | 0.473 | 0.470 | 0.529 | 0.531 | 0.527 |
|  |  | *p* | 0.410 | 0.397 | 0.404 | 0.369 | 0.363 | 0.372 |
|  | VACh | *r* | 0.337 | 0.327 | 0.331 | 0.254 | 0.250 | 0.252 |
|  |  | *p* | 0.563 | 0.583 | 0.576 | 0.690 | 0.673 | 0.672 |
| **Zeroth-order gradient_Right** | 5HT1A | *r* | 0.783 | 0.780 | 0.778 | 0.660 | 0.614 | 0.641 |
|  |  | *p* | 0.141 | 0.133 | 0.138 | 0.239 | 0.282 | 0.255 |
|  | 5HT1B | *r* | 0.117 | 0.119 | 0.119 | 0.223 | 0.250 | 0.239 |
|  |  | *p* | 0.917 | 0.912 | 0.913 | 0.725 | 0.665 | 0.688 |
|  | 5HT2A | *r* | 0.050 | 0.052 | 0.053 | 0.098 | 0.120 | 0.110 |
|  |  | *p* | 0.978 | 0.981 | 0.976 | 0.822 | 0.802 | 0.825 |
|  | 5HT4 | *r* | 0.551 | 0.546 | 0.546 | 0.443 | 0.397 | 0.421 |
|  |  | *p* | 0.358 | 0.350 | 0.353 | 0.523 | 0.653 | 0.582 |
|  | CB1 | *r* | 0.445 | 0.442 | 0.440 | 0.393 | 0.365 | 0.383 |
|  |  | *p* | 0.358 | 0.350 | 0.353 | 0.578 | 0.665 | 0.617 |
|  | D1 | *r* | 0.037 | 0.036 | 0.039 | 0.088 | 0.093 | 0.086 |
|  |  | *p* | 0.978 | 0.981 | 0.976 | 0.822 | 0.811 | 0.825 |
|  | D2 | *r* | 0.460 | 0.460 | 0.457 | 0.343 | 0.311 | 0.329 |
|  |  | *p* | 0.358 | 0.350 | 0.353 | 0.578 | 0.665 | 0.617 |
|  | DAT | *r* | 0.000 | 0.000 | 0.005 | 0.134 | 0.170 | 0.146 |
|  |  | *p* | 0.999 | 0.999 | 0.990 | 0.822 | 0.795 | 0.797 |
|  | FDOPA | *r* | 0.573 | 0.575 | 0.574 | 0.623 | 0.629 | 0.627 |
|  |  | *p* | 0.358 | 0.350 | 0.353 | 0.264 | 0.282 | 0.255 |
|  | GABAa | *r* | 0.336 | 0.334 | 0.328 | 0.188 | 0.139 | 0.171 |
|  |  | *p* | 0.522 | 0.526 | 0.538 | 0.754 | 0.802 | 0.793 |
|  | KappaOp | *r* | 0.768 | 0.772 | 0.769 | 0.827 | 0.841 | 0.838 |
|  |  | *p* | 0.141 | 0.133 | 0.138 | 0.107 | 0.081 | 0.086 |
|  | mGluR5 | *r* | 0.471 | 0.469 | 0.464 | 0.330 | 0.282 | 0.313 |
|  |  | *p* | 0.358 | 0.350 | 0.353 | 0.578 | 0.665 | 0.617 |
|  | MU | *r* | 0.438 | 0.445 | 0.441 | 0.535 | 0.567 | 0.557 |
|  |  | *p* | 0.358 | 0.350 | 0.353 | 0.354 | 0.290 | 0.321 |
|  | NA | *r* | 0.254 | 0.247 | 0.257 | 0.269 | 0.267 | 0.259 |
|  |  | *p* | 0.656 | 0.670 | 0.648 | 0.671 | 0.665 | 0.688 |
|  | NMDA | *r* | 0.463 | 0.466 | 0.466 | 0.538 | 0.565 | 0.550 |
|  |  | *p* | 0.358 | 0.350 | 0.353 | 0.354 | 0.290 | 0.321 |
|  | SERT_DAS | *r* | 0.618 | 0.623 | 0.619 | 0.699 | 0.725 | 0.719 |
|  |  | *p* | 0.343 | 0.329 | 0.339 | 0.217 | 0.163 | 0.175 |
|  | SERT_MADAM | *r* | 0.720 | 0.725 | 0.720 | 0.782 | 0.799 | 0.799 |
|  |  | *p* | 0.172 | 0.162 | 0.171 | 0.115 | 0.088 | 0.088 |
|  | VACh | *r* | 0.479 | 0.477 | 0.475 | 0.340 | 0.297 | 0.321 |
|  |  | *p* | 0.358 | 0.350 | 0.353 | 0.578 | 0.665 | 0.617 |
| **First-order gradient_Left** | 5HT1A | *r* | 0.549 | 0.515 | 0.518 | 0.038 | 0.043 | 0.066 |
|  |  | *p* | 0.385 | 0.477 | 0.459 | 0.945 | 0.946 | 0.951 |
|  | 5HT1B | *r* | 0.393 | 0.410 | 0.403 | 0.610 | 0.613 | 0.627 |
|  |  | *p* | 0.531 | 0.573 | 0.565 | 0.292 | 0.286 | 0.254 |
|  | 5HT2A | *r* | 0.171 | 0.215 | 0.199 | 0.371 | 0.364 | 0.351 |
|  |  | *p* | 0.849 | 0.860 | 0.829 | 0.898 | 0.881 | 0.862 |
|  | 5HT4 | *r* | 0.494 | 0.448 | 0.460 | 0.066 | 0.070 | 0.064 |
|  |  | *p* | 0.454 | 0.573 | 0.549 | 0.945 | 0.946 | 0.951 |
|  | CB1 | *r* | 0.368 | 0.342 | 0.342 | 0.094 | 0.096 | 0.098 |
|  |  | *p* | 0.539 | 0.603 | 0.601 | 0.945 | 0.946 | 0.951 |
|  | D1 | *r* | 0.107 | 0.084 | 0.093 | 0.222 | 0.235 | 0.255 |
|  |  | *p* | 0.901 | 0.941 | 0.914 | 0.945 | 0.946 | 0.913 |
|  | D2 | *r* | 0.546 | 0.512 | 0.519 | 0.040 | 0.045 | 0.074 |
|  |  | *p* | 0.385 | 0.477 | 0.459 | 0.945 | 0.946 | 0.951 |
|  | DAT | *r* | 0.074 | 0.029 | 0.057 | 0.299 | 0.297 | 0.309 |
|  |  | *p* | 0.901 | 0.941 | 0.937 | 0.945 | 0.923 | 0.862 |
|  | FDOPA | *r* | 0.197 | 0.183 | 0.184 | 0.274 | 0.283 | 0.301 |
|  |  | *p* | 0.847 | 0.860 | 0.829 | 0.945 | 0.923 | 0.862 |
|  | GABAa | *r* | 0.402 | 0.352 | 0.370 | 0.122 | 0.122 | 0.132 |
|  |  | *p* | 0.531 | 0.603 | 0.589 | 0.945 | 0.946 | 0.951 |
|  | KappaOp | *r* | 0.758 | 0.765 | 0.763 | 0.837 * | 0.838 * | 0.844 * |
|  |  | *p* | 0.305 | 0.224 | 0.269 | 0.030 | 0.029 | 0.025 |
|  | mGluR5 | *r* | 0.450 | 0.400 | 0.418 | 0.055 | 0.054 | 0.063 |
|  |  | *p* | 0.504 | 0.573 | 0.565 | 0.945 | 0.946 | 0.951 |
|  | MU | *r* | 0.629 | 0.641 | 0.642 | 0.859 * | 0.863 * | 0.875 * |
|  |  | *p* | 0.313 | 0.284 | 0.281 | 0.027 | 0.024 | 0.018 |
|  | NA | *r* | 0.099 | 0.074 | 0.093 | 0.027 | 0.026 | 0.014 |
|  |  | *p* | 0.901 | 0.941 | 0.914 | 0.945 | 0.946 | 0.971 |
|  | NMDA | *r* | 0.016 | 0.055 | 0.028 | 0.074 | 0.062 | 0.050 |
|  |  | *p* | 0.968 | 0.941 | 0.942 | 0.945 | 0.946 | 0.951 |
|  | SERT_DAS | *r* | 0.665 | 0.698 | 0.678 | 0.673 | 0.662 | 0.655 |
|  |  | *p* | 0.305 | 0.224 | 0.269 | 0.212 | 0.234 | 0.251 |
|  | SERT_MADAM | *r* | 0.666 | 0.696 | 0.683 | 0.871 * | 0.868 * | 0.875 * |
|  |  | *p* | 0.305 | 0.224 | 0.269 | 0.027 | 0.024 | 0.018 |
|  | VACh | *r* | 0.206 | 0.166 | 0.179 | 0.354 | 0.359 | 0.383 |
|  |  | *p* | 0.847 | 0.860 | 0.829 | 0.898 | 0.881 | 0.862 |
| **First-order gradient_Right** | 5HT1A | *r* | 0.418 | 0.363 | 0.387 | 0.628 | 0.704 | 0.691 |
|  |  | *p* | 0.689 | 0.838 | 0.780 | 0.235 | 0.201 | 0.185 |
|  | 5HT1B | *r* | 0.325 | 0.326 | 0.341 | 0.614 | 0.598 | 0.609 |
|  |  | *p* | 0.826 | 0.838 | 0.832 | 0.235 | 0.201 | 0.185 |
|  | 5HT2A | *r* | 0.144 | 0.170 | 0.153 | 0.250 | 0.244 | 0.240 |
|  |  | *p* | 0.854 | 0.838 | 0.834 | 0.574 | 0.627 | 0.618 |
|  | 5HT4 | *r* | 0.190 | 0.121 | 0.159 | 0.585 | 0.637 | 0.623 |
|  |  | *p* | 0.826 | 0.838 | 0.834 | 0.249 | 0.201 | 0.185 |
|  | CB1 | *r* | 0.197 | 0.161 | 0.184 | 0.275 | 0.312 | 0.298 |
|  |  | *p* | 0.826 | 0.838 | 0.834 | 0.574 | 0.570 | 0.578 |
|  | D1 | *r* | 0.047 | 0.001 | 0.044 | 0.236 | 0.227 | 0.231 |
|  |  | *p* | 0.904 | 0.998 | 0.910 | 0.574 | 0.627 | 0.618 |
|  | D2 | *r* | 0.271 | 0.251 | 0.249 | 0.568 | 0.614 | 0.618 |
|  |  | *p* | 0.826 | 0.838 | 0.834 | 0.249 | 0.201 | 0.185 |
|  | DAT | *r* | 0.094 | 0.103 | 0.079 | 0.528 | 0.538 | 0.538 |
|  |  | *p* | 0.904 | 0.838 | 0.890 | 0.287 | 0.271 | 0.271 |
|  | FDOPA | *r* | 0.465 | 0.428 | 0.463 | 0.248 | 0.190 | 0.208 |
|  |  | *p* | 0.689 | 0.804 | 0.756 | 0.574 | 0.660 | 0.627 |
|  | GABAa | *r* | 0.180 | 0.166 | 0.159 | 0.658 | 0.691 | 0.681 |
|  |  | *p* | 0.826 | 0.838 | 0.834 | 0.235 | 0.201 | 0.185 |
|  | KappaOp | *r* | 0.879 * | 0.844 * | 0.875 * | 0.420 | 0.326 | 0.346 |
|  |  | *p* | 0.018 | 0.033 | 0.018 | 0.391 | 0.570 | 0.544 |
|  | mGluR5 | *r* | 0.301 | 0.277 | 0.278 | 0.622 | 0.670 | 0.657 |
|  |  | *p* | 0.826 | 0.838 | 0.834 | 0.235 | 0.201 | 0.185 |
|  | MU | *r* | 0.827 * | 0.831 * | 0.838 * | 0.698 | 0.631 | 0.648 |
|  |  | *p* | 0.036 | 0.033 | 0.029 | 0.235 | 0.201 | 0.185 |
|  | NA | *r* | 0.058 | 0.130 | 0.080 | 0.115 | 0.139 | 0.142 |
|  |  | *p* | 0.904 | 0.838 | 0.890 | 0.769 | 0.721 | 0.716 |
|  | NMDA | *r* | 0.414 | 0.414 | 0.421 | 0.338 | 0.294 | 0.289 |
|  |  | *p* | 0.689 | 0.804 | 0.778 | 0.518 | 0.570 | 0.578 |
|  | SERT_DAS | *r* | 0.735 | 0.747 | 0.747 | 0.511 | 0.444 | 0.460 |
|  |  | *p* | 0.108 | 0.093 | 0.093 | 0.287 | 0.415 | 0.384 |
|  | SERT_MADAM | *r* | 0.875 * | 0.872 * | 0.881 * | 0.464 | 0.381 | 0.401 |
|  |  | *p* | 0.018 | 0.033 | 0.018 | 0.340 | 0.511 | 0.466 |
|  | VACh | *r* | 0.251 | 0.217 | 0.222 | 0.680 | 0.730 | 0.727 |
|  |  | *p* | 0.826 | 0.838 | 0.834 | 0.235 | 0.201 | 0.185 |
| **Second-order gradient_Left** | 5HT1A | *r* | 0.185 | 0.319 | 0.309 | 0.219 | 0.259 | 0.202 |
|  |  | *p* | 0.768 | 0.578 | 0.580 | 0.642 | 0.601 | 0.637 |
|  | 5HT1B | *r* | 0.419 | 0.467 | 0.446 | 0.371 | 0.385 | 0.308 |
|  |  | *p* | 0.577 | 0.528 | 0.580 | 0.588 | 0.577 | 0.634 |
|  | 5HT2A | *r* | 0.546 | 0.471 | 0.497 | 0.676 | 0.659 | 0.704 |
|  |  | *p* | 0.577 | 0.528 | 0.580 | 0.206 | 0.241 | 0.205 |
|  | 5HT4 | *r* | 0.435 | 0.310 | 0.323 | 0.420 | 0.385 | 0.422 |
|  |  | *p* | 0.577 | 0.578 | 0.580 | 0.588 | 0.577 | 0.634 |
|  | CB1 | *r* | 0.383 | 0.315 | 0.316 | 0.363 | 0.342 | 0.345 |
|  |  | *p* | 0.577 | 0.578 | 0.580 | 0.588 | 0.577 | 0.634 |
|  | D1 | *r* | 0.960 ** | 0.947 ** | 0.948 ** | 0.920 ** | 0.917 ** | 0.889 * |
|  |  | *p* | 0.001 | 0.002 | 0.002 | 0.008 | 0.009 | 0.024 |
|  | D2 | *r* | 0.274 | 0.415 | 0.402 | 0.310 | 0.351 | 0.282 |
|  |  | *p* | 0.712 | 0.578 | 0.580 | 0.588 | 0.577 | 0.634 |
|  | DAT | *r* | 0.054 | 0.182 | 0.141 | 0.152 | 0.127 | 0.223 |
|  |  | *p* | 0.891 | 0.768 | 0.809 | 0.738 | 0.788 | 0.634 |
|  | FDOPA | *r* | 0.786 | 0.783 | 0.775 | 0.704 | 0.702 | 0.661 |
|  |  | *p* | 0.108 | 0.113 | 0.127 | 0.205 | 0.210 | 0.237 |
|  | GABAa | *r* | 0.146 | 0.002 | 0.026 | 0.251 | 0.216 | 0.285 |
|  |  | *p* | 0.768 | 0.996 | 0.948 | 0.618 | 0.650 | 0.634 |
|  | KappaOp | *r* | 0.384 | 0.381 | 0.366 | 0.285 | 0.293 | 0.226 |
|  |  | *p* | 0.577 | 0.578 | 0.580 | 0.588 | 0.594 | 0.634 |
|  | mGluR5 | *r* | 0.227 | 0.078 | 0.105 | 0.317 | 0.282 | 0.347 |
|  |  | *p* | 0.768 | 0.892 | 0.835 | 0.588 | 0.594 | 0.634 |
|  | MU | *r* | 0.476 | 0.497 | 0.485 | 0.414 | 0.432 | 0.357 |
|  |  | *p* | 0.577 | 0.528 | 0.580 | 0.588 | 0.577 | 0.634 |
|  | NA | *r* | 0.173 | 0.111 | 0.140 | 0.426 | 0.428 | 0.455 |
|  |  | *p* | 0.768 | 0.873 | 0.809 | 0.588 | 0.577 | 0.634 |
|  | NMDA | *r* | 0.667 | 0.600 | 0.628 | 0.837 * | 0.827 | 0.861 * |
|  |  | *p* | 0.297 | 0.528 | 0.421 | 0.044 | 0.054 | 0.026 |
|  | SERT_DAS | *r* | 0.361 | 0.329 | 0.358 | 0.491 | 0.480 | 0.546 |
|  |  | *p* | 0.577 | 0.578 | 0.580 | 0.588 | 0.577 | 0.461 |
|  | SERT_MADAM | *r* | 0.137 | 0.188 | 0.160 | 0.027 | 0.047 | 0.045 |
|  |  | *p* | 0.768 | 0.768 | 0.809 | 0.945 | 0.905 | 0.909 |
|  | VACh | *r* | 0.352 | 0.491 | 0.464 | 0.295 | 0.331 | 0.236 |
|  |  | *p* | 0.577 | 0.528 | 0.580 | 0.588 | 0.577 | 0.634 |
| **Second-order gradient_Right** | 5HT1A | *r* | 0.437 | 0.431 | 0.366 | 0.816 * | 0.936 ** | 0.899 * |
|  |  | *p* | 0.705 | 0.690 | 0.706 | 0.044 | 0.004 | 0.017 |
|  | 5HT1B | *r* | 0.370 | 0.386 | 0.379 | 0.144 | 0.080 | 0.008 |
|  |  | *p* | 0.705 | 0.690 | 0.706 | 0.754 | 0.975 | 0.983 |
|  | 5HT2A | *r* | 0.468 | 0.424 | 0.465 | 0.486 | 0.252 | 0.342 |
|  |  | *p* | 0.705 | 0.690 | 0.706 | 0.332 | 0.659 | 0.508 |
|  | 5HT4 | *r* | 0.024 | 0.009 | 0.079 | 0.875 * | 0.790 | 0.835 * |
|  |  | *p* | 0.978 | 0.993 | 0.910 | 0.036 | 0.053 | 0.030 |
|  | CB1 | *r* | 0.011 | 0.004 | 0.044 | 0.672 | 0.553 | 0.595 |
|  |  | *p* | 0.978 | 0.993 | 0.910 | 0.144 | 0.275 | 0.235 |
|  | D1 | *r* | 0.823 | 0.809 | 0.833 | 0.404 | 0.057 | 0.214 |
|  |  | *p* | 0.116 | 0.148 | 0.096 | 0.362 | 0.975 | 0.696 |
|  | D2 | *r* | 0.495 | 0.496 | 0.455 | 0.411 | 0.655 | 0.567 |
|  |  | *p* | 0.705 | 0.690 | 0.706 | 0.362 | 0.167 | 0.251 |
|  | DAT | *r* | 0.256 | 0.290 | 0.248 | 0.418 | 0.524 | 0.470 |
|  |  | *p* | 0.705 | 0.690 | 0.706 | 0.362 | 0.295 | 0.330 |
|  | FDOPA | *r* | 0.309 | 0.321 | 0.354 | 0.575 | 0.388 | 0.478 |
|  |  | *p* | 0.705 | 0.690 | 0.706 | 0.267 | 0.495 | 0.330 |
|  | GABAa | *r* | 0.301 | 0.335 | 0.272 | 0.750 | 0.787 | 0.756 |
|  |  | *p* | 0.705 | 0.690 | 0.706 | 0.090 | 0.053 | 0.083 |
|  | KappaOp | *r* | 0.159 | 0.195 | 0.232 | 0.671 | 0.587 | 0.651 |
|  |  | *p* | 0.831 | 0.792 | 0.706 | 0.144 | 0.247 | 0.172 |
|  | mGluR5 | *r* | 0.307 | 0.332 | 0.264 | 0.832 * | 0.872 * | 0.847 * |
|  |  | *p* | 0.705 | 0.690 | 0.706 | 0.044 | 0.020 | 0.030 |
|  | MU | *r* | 0.254 | 0.283 | 0.298 | 0.406 | 0.296 | 0.359 |
|  |  | *p* | 0.705 | 0.690 | 0.706 | 0.362 | 0.658 | 0.508 |
|  | NA | *r* | 0.031 | 0.075 | 0.068 | 0.096 | 0.039 | 0.020 |
|  |  | *p* | 0.978 | 0.955 | 0.910 | 0.805 | 0.975 | 0.983 |
|  | NMDA | *r* | 0.342 | 0.291 | 0.310 | 0.214 | 0.005 | 0.065 |
|  |  | *p* | 0.705 | 0.690 | 0.706 | 0.697 | 0.990 | 0.977 |
|  | SERT_DAS | *r* | 0.305 | 0.259 | 0.260 | 0.178 | 0.256 | 0.232 |
|  |  | *p* | 0.705 | 0.694 | 0.706 | 0.728 | 0.659 | 0.696 |
|  | SERT_MADAM | *r* | 0.154 | 0.115 | 0.094 | 0.487 | 0.492 | 0.501 |
|  |  | *p* | 0.831 | 0.922 | 0.910 | 0.332 | 0.321 | 0.330 |
|  | VACh | *r* | 0.454 | 0.458 | 0.405 | 0.558 | 0.756 | 0.687 |
|  |  | *p* | 0.705 | 0.690 | 0.706 | 0.267 | 0.066 | 0.147 |
| **Third-order gradient_Left** | 5HT1A | *r* | 0.894 * | 0.913 * | 0.913 * | 0.961 *** | 0.961 *** | 0.960 *** |
|  |  | *p* | 0.020 | 0.011 | 0.011 | 0.001 | 0.001 | 0.001 |
|  | 5HT1B | *r* | 0.064 | 0.045 | 0.058 | 0.039 | 0.037 | 0.044 |
|  |  | *p* | 0.870 | 0.995 | 0.989 | 0.929 | 0.924 | 0.944 |
|  | 5HT2A | *r* | 0.137 | 0.220 | 0.214 | 0.221 | 0.225 | 0.214 |
|  |  | *p* | 0.870 | 0.855 | 0.881 | 0.929 | 0.919 | 0.944 |
|  | 5HT4 | *r* | 0.768 | 0.823 * | 0.821 * | 0.808 * | 0.810 * | 0.803 * |
|  |  | *p* | 0.094 | 0.038 | 0.040 | 0.030 | 0.029 | 0.033 |
|  | CB1 | *r* | 0.598 | 0.621 | 0.627 | 0.569 | 0.571 | 0.561 |
|  |  | *p* | 0.266 | 0.222 | 0.212 | 0.283 | 0.279 | 0.298 |
|  | D1 | *r* | 0.113 | 0.159 | 0.158 | 0.035 | 0.037 | 0.028 |
|  |  | *p* | 0.870 | 0.930 | 0.889 | 0.929 | 0.924 | 0.944 |
|  | D2 | *r* | 0.835 * | 0.859 * | 0.854 * | 0.936 ** | 0.936 ** | 0.939 ** |
|  |  | *p* | 0.045 | 0.027 | 0.031 | 0.002 | 0.002 | 0.002 |
|  | DAT | *r* | 0.122 | 0.227 | 0.210 | 0.409 | 0.411 | 0.413 |
|  |  | *p* | 0.870 | 0.855 | 0.881 | 0.619 | 0.613 | 0.606 |
|  | FDOPA | *r* | 0.246 | 0.262 | 0.266 | 0.121 | 0.123 | 0.113 |
|  |  | *p* | 0.870 | 0.855 | 0.879 | 0.929 | 0.924 | 0.944 |
|  | GABAa | *r* | 0.643 | 0.723 | 0.717 | 0.813 * | 0.815 * | 0.811 * |
|  |  | *p* | 0.222 | 0.100 | 0.107 | 0.030 | 0.029 | 0.033 |
|  | KappaOp | *r* | 0.359 | 0.353 | 0.363 | 0.292 | 0.292 | 0.291 |
|  |  | *p* | 0.685 | 0.790 | 0.758 | 0.874 | 0.882 | 0.872 |
|  | mGluR5 | *r* | 0.660 | 0.743 | 0.736 | 0.822 * | 0.824 * | 0.820 * |
|  |  | *p* | 0.222 | 0.098 | 0.107 | 0.030 | 0.029 | 0.033 |
|  | MU | *r* | 0.086 | 0.091 | 0.099 | 0.040 | 0.040 | 0.039 |
|  |  | *p* | 0.870 | 0.978 | 0.961 | 0.929 | 0.924 | 0.944 |
|  | NA | *r* | 0.092 | 0.001 | 0.005 | 0.136 | 0.141 | 0.135 |
|  |  | *p* | 0.870 | 0.998 | 0.989 | 0.929 | 0.924 | 0.944 |
|  | NMDA | *r* | 0.078 | 0.030 | 0.016 | 0.081 | 0.086 | 0.079 |
|  |  | *p* | 0.870 | 0.995 | 0.989 | 0.929 | 0.924 | 0.944 |
|  | SERT_DAS | *r* | 0.368 | 0.292 | 0.310 | 0.268 | 0.265 | 0.269 |
|  |  | *p* | 0.685 | 0.855 | 0.832 | 0.874 | 0.882 | 0.872 |
|  | SERT_MADAM | *r* | 0.184 | 0.138 | 0.155 | 0.071 | 0.070 | 0.069 |
|  |  | *p* | 0.870 | 0.930 | 0.889 | 0.929 | 0.924 | 0.944 |
|  | VACh | *r* | 0.552 | 0.600 | 0.589 | 0.724 | 0.724 | 0.728 |
|  |  | *p* | 0.317 | 0.226 | 0.245 | 0.082 | 0.082 | 0.078 |
| **Third-order gradient_Right** | 5HT1A | *r* | 0.908 * | 0.915 * | 0.924 ** | 0.951 ** | 0.725 | 0.906 * |
|  |  | *p* | 0.013 | 0.010 | 0.007 | 0.002 | 0.163 | 0.014 |
|  | 5HT1B | *r* | 0.043 | 0.060 | 0.072 | 0.242 | 0.689 | 0.382 |
|  |  | *p* | 0.912 | 0.879 | 0.876 | 0.732 | 0.181 | 0.599 |
|  | 5HT2A | *r* | 0.153 | 0.152 | 0.122 | 0.034 | 0.190 | 0.112 |
|  |  | *p* | 0.797 | 0.798 | 0.848 | 0.931 | 0.802 | 0.871 |
|  | 5HT4 | *r* | 0.780 | 0.778 | 0.767 | 0.676 | 0.351 | 0.542 |
|  |  | *p* | 0.119 | 0.122 | 0.144 | 0.136 | 0.639 | 0.393 |
|  | CB1 | *r* | 0.516 | 0.508 | 0.499 | 0.393 | 0.022 | 0.247 |
|  |  | *p* | 0.349 | 0.367 | 0.385 | 0.531 | 0.963 | 0.674 |
|  | D1 | *r* | 0.114 | 0.100 | 0.061 | 0.164 | 0.486 | 0.352 |
|  |  | *p* | 0.816 | 0.845 | 0.876 | 0.732 | 0.475 | 0.599 |
|  | D2 | *r* | 0.554 | 0.577 | 0.588 | 0.770 | 0.919 ** | 0.848 * |
|  |  | *p* | 0.312 | 0.274 | 0.274 | 0.069 | 0.008 | 0.023 |
|  | DAT | *r* | 0.252 | 0.281 | 0.281 | 0.494 | 0.533 | 0.519 |
|  |  | *p* | 0.711 | 0.675 | 0.642 | 0.401 | 0.475 | 0.393 |
|  | FDOPA | *r* | 0.418 | 0.409 | 0.391 | 0.224 | 0.271 | 0.055 |
|  |  | *p* | 0.473 | 0.493 | 0.536 | 0.732 | 0.664 | 0.888 |
|  | GABAa | *r* | 0.557 | 0.573 | 0.573 | 0.679 | 0.450 | 0.615 |
|  |  | *p* | 0.312 | 0.274 | 0.274 | 0.136 | 0.504 | 0.282 |
|  | KappaOp | *r* | 0.585 | 0.589 | 0.577 | 0.492 | 0.018 | 0.363 |
|  |  | *p* | 0.312 | 0.274 | 0.274 | 0.401 | 0.963 | 0.599 |
|  | mGluR5 | *r* | 0.669 | 0.685 | 0.685 | 0.772 | 0.487 | 0.697 |
|  |  | *p* | 0.234 | 0.193 | 0.188 | 0.069 | 0.475 | 0.166 |
|  | MU | *r* | 0.203 | 0.213 | 0.199 | 0.172 | 0.304 | 0.063 |
|  |  | *p* | 0.772 | 0.748 | 0.780 | 0.732 | 0.639 | 0.888 |
|  | NA | *r* | 0.322 | 0.303 | 0.309 | 0.155 | 0.361 | 0.201 |
|  |  | *p* | 0.652 | 0.675 | 0.642 | 0.732 | 0.639 | 0.724 |
|  | NMDA | *r* | 0.146 | 0.145 | 0.162 | 0.186 | 0.305 | 0.285 |
|  |  | *p* | 0.797 | 0.798 | 0.811 | 0.732 | 0.639 | 0.674 |
|  | SERT_DAS | *r* | 0.267 | 0.267 | 0.281 | 0.272 | 0.086 | 0.246 |
|  |  | *p* | 0.711 | 0.675 | 0.642 | 0.732 | 0.952 | 0.674 |
|  | SERT_MADAM | *r* | 0.446 | 0.450 | 0.454 | 0.430 | 0.076 | 0.343 |
|  |  | *p* | 0.458 | 0.448 | 0.439 | 0.497 | 0.952 | 0.599 |
|  | VACh | *r* | 0.662 | 0.682 | 0.692 | 0.837 * | 0.893 * | 0.879 * |
|  |  | *p* | 0.234 | 0.193 | 0.188 | 0.044 | 0.011 | 0.016 |
| **Fourth-order gradient_Left** | 5HT1A | *r* | 0.546 | 0.346 | 0.304 | 0.071 | 0.065 | 0.091 |
|  |  | *p* | 0.247 | 0.543 | 0.598 | 0.906 | 0.920 | 0.864 |
|  | 5HT1B | *r* | 0.271 | 0.264 | 0.301 | 0.410 | 0.404 | 0.435 |
|  |  | *p* | 0.618 | 0.681 | 0.598 | 0.614 | 0.633 | 0.607 |
|  | 5HT2A | *r* | 0.543 | 0.560 | 0.523 | 0.291 | 0.299 | 0.211 |
|  |  | *p* | 0.247 | 0.300 | 0.381 | 0.649 | 0.665 | 0.725 |
|  | 5HT4 | *r* | 0.535 | 0.413 | 0.344 | 0.113 | 0.100 | 0.181 |
|  |  | *p* | 0.247 | 0.503 | 0.596 | 0.869 | 0.897 | 0.725 |
|  | CB1 | *r* | 0.282 | 0.177 | 0.117 | 0.239 | 0.229 | 0.299 |
|  |  | *p* | 0.618 | 0.793 | 0.810 | 0.649 | 0.665 | 0.651 |
|  | D1 | *r* | 0.018 | 0.037 | 0.021 | 0.263 | 0.253 | 0.340 |
|  |  | *p* | 0.964 | 0.925 | 0.958 | 0.649 | 0.665 | 0.607 |
|  | D2 | *r* | 0.565 | 0.380 | 0.348 | 0.018 | 0.023 | 0.012 |
|  |  | *p* | 0.247 | 0.513 | 0.596 | 0.964 | 0.952 | 0.975 |
|  | DAT | *r* | 0.897 * | 0.897 * | 0.897 * | 0.741 | 0.745 | 0.717 |
|  |  | *p* | 0.019 | 0.019 | 0.019 | 0.402 | 0.381 | 0.535 |
|  | FDOPA | *r* | 0.117 | 0.116 | 0.173 | 0.424 | 0.416 | 0.487 |
|  |  | *p* | 0.817 | 0.811 | 0.738 | 0.614 | 0.633 | 0.607 |
|  | GABAa | *r* | 0.831 * | 0.720 | 0.674 | 0.279 | 0.289 | 0.225 |
|  |  | *p* | 0.048 | 0.154 | 0.254 | 0.649 | 0.665 | 0.725 |
|  | KappaOp | *r* | 0.113 | 0.164 | 0.206 | 0.457 | 0.455 | 0.468 |
|  |  | *p* | 0.817 | 0.793 | 0.738 | 0.614 | 0.633 | 0.607 |
|  | mGluR5 | *r* | 0.810 * | 0.704 | 0.653 | 0.236 | 0.246 | 0.179 |
|  |  | *p* | 0.048 | 0.154 | 0.254 | 0.649 | 0.665 | 0.725 |
|  | MU | *r* | 0.167 | 0.147 | 0.178 | 0.338 | 0.337 | 0.348 |
|  |  | *p* | 0.801 | 0.793 | 0.738 | 0.649 | 0.665 | 0.607 |
|  | NA | *r* | 0.691 | 0.738 | 0.730 | 0.596 | 0.599 | 0.560 |
|  |  | *p* | 0.141 | 0.154 | 0.230 | 0.614 | 0.633 | 0.607 |
|  | NMDA | *r* | 0.541 | 0.637 | 0.603 | 0.417 | 0.429 | 0.346 |
|  |  | *p* | 0.247 | 0.235 | 0.308 | 0.614 | 0.633 | 0.607 |
|  | SERT_DAS | *r* | 0.307 | 0.433 | 0.440 | 0.507 | 0.511 | 0.462 |
|  |  | *p* | 0.618 | 0.503 | 0.498 | 0.614 | 0.633 | 0.607 |
|  | SERT_MADAM | *r* | 0.361 | 0.405 | 0.429 | 0.530 | 0.531 | 0.518 |
|  |  | *p* | 0.555 | 0.503 | 0.498 | 0.614 | 0.633 | 0.607 |
|  | VACh | *r* | 0.707 | 0.579 | 0.572 | 0.353 | 0.354 | 0.350 |
|  |  | *p* | 0.141 | 0.300 | 0.322 | 0.649 | 0.665 | 0.607 |
| **Fourth-order gradient_Right** | 5HT1A | *r* | 0.397 | 0.273 | 0.372 | 0.193 | 0.268 | 0.322 |
|  |  | *p* | 0.475 | 0.613 | 0.486 | 0.856 | 0.796 | 0.717 |
|  | 5HT1B | *r* | 0.312 | 0.316 | 0.289 | 0.366 | 0.223 | 0.323 |
|  |  | *p* | 0.573 | 0.587 | 0.625 | 0.666 | 0.846 | 0.717 |
|  | 5HT2A | *r* | 0.675 | 0.687 | 0.709 | 0.487 | 0.420 | 0.450 |
|  |  | *p* | 0.203 | 0.148 | 0.145 | 0.472 | 0.670 | 0.576 |
|  | 5HT4 | *r* | 0.553 | 0.469 | 0.558 | 0.015 | 0.053 | 0.095 |
|  |  | *p* | 0.314 | 0.457 | 0.305 | 0.969 | 0.945 | 0.908 |
|  | CB1 | *r* | 0.366 | 0.305 | 0.383 | 0.072 | 0.053 | 0.147 |
|  |  | *p* | 0.498 | 0.587 | 0.486 | 0.905 | 0.945 | 0.908 |
|  | D1 | *r* | 0.195 | 0.209 | 0.237 | 0.085 | 0.010 | 0.097 |
|  |  | *p* | 0.738 | 0.700 | 0.646 | 0.905 | 0.980 | 0.908 |
|  | D2 | *r* | 0.448 | 0.370 | 0.413 | 0.136 | 0.075 | 0.039 |
|  |  | *p* | 0.407 | 0.587 | 0.485 | 0.873 | 0.945 | 0.943 |
|  | DAT | *r* | 0.826 | 0.795 | 0.805 | 0.629 | 0.340 | 0.552 |
|  |  | *p* | 0.054 | 0.112 | 0.080 | 0.312 | 0.796 | 0.443 |
|  | FDOPA | *r* | 0.005 | 0.051 | 0.023 | 0.338 | 0.320 | 0.352 |
|  |  | *p* | 0.989 | 0.897 | 0.954 | 0.673 | 0.796 | 0.717 |
|  | GABAa | *r* | 0.854 | 0.784 | 0.844 | 0.397 | 0.294 | 0.281 |
|  |  | *p* | 0.054 | 0.112 | 0.076 | 0.652 | 0.796 | 0.717 |
|  | KappaOp | *r* | 0.094 | 0.189 | 0.103 | 0.499 | 0.662 | 0.516 |
|  |  | *p* | 0.857 | 0.700 | 0.839 | 0.472 | 0.325 | 0.466 |
|  | mGluR5 | *r* | 0.781 | 0.692 | 0.767 | 0.264 | 0.136 | 0.144 |
|  |  | *p* | 0.078 | 0.148 | 0.096 | 0.738 | 0.945 | 0.908 |
|  | MU | *r* | 0.119 | 0.170 | 0.126 | 0.305 | 0.510 | 0.273 |
|  |  | *p* | 0.856 | 0.700 | 0.839 | 0.694 | 0.482 | 0.717 |
|  | NA | *r* | 0.616 | 0.648 | 0.627 | 0.632 | 0.619 | 0.639 |
|  |  | *p* | 0.232 | 0.178 | 0.211 | 0.312 | 0.325 | 0.383 |
|  | NMDA | *r* | 0.653 | 0.697 | 0.677 | 0.665 | 0.596 | 0.644 |
|  |  | *p* | 0.203 | 0.148 | 0.162 | 0.312 | 0.325 | 0.383 |
|  | SERT_DAS | *r* | 0.487 | 0.562 | 0.506 | 0.703 | 0.606 | 0.691 |
|  |  | *p* | 0.368 | 0.297 | 0.357 | 0.312 | 0.325 | 0.383 |
|  | SERT_MADAM | *r* | 0.234 | 0.327 | 0.251 | 0.587 | 0.604 | 0.597 |
|  |  | *p* | 0.701 | 0.587 | 0.646 | 0.347 | 0.325 | 0.403 |
|  | VACh | *r* | 0.526 | 0.438 | 0.492 | 0.138 | 0.090 | 0.028 |
|  |  | *p* | 0.328 | 0.476 | 0.357 | 0.873 | 0.945 | 0.943 |
| **Fifth-order gradient_Left** | 5HT1A | *r* | 0.158 | 0.177 | 0.149 | 0.225 | 0.123 | 0.541 |
|  |  | *p* | 0.771 | 0.771 | 0.742 | 0.793 | 0.994 | 0.820 |
|  | 5HT1B | *r* | 0.689 | 0.647 | 0.678 | 0.675 | 0.468 | 0.185 |
|  |  | *p* | 0.369 | 0.534 | 0.401 | 0.602 | 0.994 | 0.877 |
|  | 5HT2A | *r* | 0.398 | 0.472 | 0.527 | 0.116 | 0.260 | 0.328 |
|  |  | *p* | 0.771 | 0.771 | 0.649 | 0.920 | 0.994 | 0.820 |
|  | 5HT4 | *r* | 0.354 | 0.384 | 0.414 | 0.248 | 0.069 | 0.434 |
|  |  | *p* | 0.771 | 0.771 | 0.649 | 0.793 | 0.994 | 0.820 |
|  | CB1 | *r* | 0.687 | 0.686 | 0.699 | 0.518 | 0.053 | 0.658 |
|  |  | *p* | 0.369 | 0.534 | 0.401 | 0.690 | 0.994 | 0.820 |
|  | D1 | *r* | 0.290 | 0.286 | 0.361 | 0.025 | 0.020 | 0.030 |
|  |  | *p* | 0.771 | 0.771 | 0.649 | 0.965 | 0.994 | 0.993 |
|  | D2 | *r* | 0.165 | 0.135 | 0.158 | 0.017 | 0.072 | 0.256 |
|  |  | *p* | 0.771 | 0.771 | 0.742 | 0.965 | 0.994 | 0.820 |
|  | DAT | *r* | 0.393 | 0.257 | 0.190 | 0.245 | 0.044 | 0.313 |
|  |  | *p* | 0.771 | 0.771 | 0.742 | 0.793 | 0.994 | 0.820 |
|  | FDOPA | *r* | 0.472 | 0.442 | 0.481 | 0.239 | 0.004 | 0.233 |
|  |  | *p* | 0.771 | 0.771 | 0.649 | 0.793 | 0.994 | 0.820 |
|  | GABAa | *r* | 0.205 | 0.290 | 0.336 | 0.218 | 0.027 | 0.330 |
|  |  | *p* | 0.771 | 0.771 | 0.649 | 0.793 | 0.994 | 0.820 |
|  | KappaOp | *r* | 0.178 | 0.138 | 0.180 | 0.379 | 0.368 | 0.003 |
|  |  | *p* | 0.771 | 0.771 | 0.742 | 0.793 | 0.994 | 0.994 |
|  | mGluR5 | *r* | 0.164 | 0.247 | 0.300 | 0.183 | 0.003 | 0.265 |
|  |  | *p* | 0.771 | 0.771 | 0.649 | 0.819 | 0.994 | 0.820 |
|  | MU | *r* | 0.040 | 0.015 | 0.082 | 0.258 | 0.461 | 0.287 |
|  |  | *p* | 0.918 | 0.970 | 0.835 | 0.793 | 0.994 | 0.820 |
|  | NA | *r* | 0.235 | 0.358 | 0.462 | 0.351 | 0.311 | 0.076 |
|  |  | *p* | 0.771 | 0.771 | 0.649 | 0.793 | 0.994 | 0.951 |
|  | NMDA | *r* | 0.068 | 0.172 | 0.302 | 0.055 | 0.255 | 0.362 |
|  |  | *p* | 0.912 | 0.771 | 0.649 | 0.965 | 0.994 | 0.820 |
|  | SERT_DAS | *r* | 0.285 | 0.206 | 0.155 | 0.582 | 0.299 | 0.343 |
|  |  | *p* | 0.771 | 0.771 | 0.742 | 0.602 | 0.994 | 0.820 |
|  | SERT_MADAM | *r* | 0.386 | 0.316 | 0.322 | 0.602 | 0.453 | 0.125 |
|  |  | *p* | 0.771 | 0.771 | 0.649 | 0.602 | 0.994 | 0.911 |
|  | VACh | *r* | 0.379 | 0.310 | 0.320 | 0.253 | 0.252 | 0.119 |
|  |  | *p* | 0.771 | 0.771 | 0.649 | 0.793 | 0.994 | 0.911 |
| **Fifth-order gradient_Right** | 5HT1A | *r* | 0.106 | 0.053 | 0.044 | 0.520 | 0.531 | 0.495 |
|  |  | *p* | 0.957 | 0.925 | 0.949 | 0.679 | 0.695 | 0.863 |
|  | 5HT1B | *r* | 0.812 | 0.755 | 0.793 | 0.066 | 0.219 | 0.032 |
|  |  | *p* | 0.140 | 0.169 | 0.195 | 0.900 | 0.856 | 0.941 |
|  | 5HT2A | *r* | 0.337 | 0.415 | 0.290 | 0.222 | 0.283 | 0.169 |
|  |  | *p* | 0.844 | 0.600 | 0.863 | 0.900 | 0.753 | 0.941 |
|  | 5HT4 | *r* | 0.220 | 0.383 | 0.244 | 0.539 | 0.321 | 0.479 |
|  |  | *p* | 0.855 | 0.619 | 0.863 | 0.679 | 0.753 | 0.863 |
|  | CB1 | *r* | 0.663 | 0.759 | 0.681 | 0.599 | 0.412 | 0.599 |
|  |  | *p* | 0.197 | 0.169 | 0.224 | 0.679 | 0.697 | 0.797 |
|  | D1 | *r* | 0.555 | 0.592 | 0.491 | 0.049 | 0.168 | 0.126 |
|  |  | *p* | 0.310 | 0.289 | 0.462 | 0.900 | 0.922 | 0.941 |
|  | D2 | *r* | 0.700 | 0.587 | 0.667 | 0.155 | 0.071 | 0.121 |
|  |  | *p* | 0.197 | 0.289 | 0.224 | 0.900 | 0.932 | 0.941 |
|  | DAT | *r* | 0.664 | 0.588 | 0.668 | 0.064 | 0.444 | 0.086 |
|  |  | *p* | 0.197 | 0.289 | 0.224 | 0.900 | 0.695 | 0.941 |
|  | FDOPA | *r* | 0.591 | 0.633 | 0.579 | 0.074 | 0.314 | 0.066 |
|  |  | *p* | 0.281 | 0.289 | 0.307 | 0.900 | 0.753 | 0.941 |
|  | GABAa | *r* | 0.047 | 0.075 | 0.025 | 0.424 | 0.011 | 0.415 |
|  |  | *p* | 0.957 | 0.925 | 0.949 | 0.900 | 0.978 | 0.941 |
|  | KappaOp | *r* | 0.091 | 0.135 | 0.088 | 0.274 | 0.300 | 0.276 |
|  |  | *p* | 0.957 | 0.925 | 0.949 | 0.900 | 0.753 | 0.941 |
|  | mGluR5 | *r* | 0.072 | 0.060 | 0.043 | 0.386 | 0.102 | 0.374 |
|  |  | *p* | 0.957 | 0.925 | 0.949 | 0.900 | 0.932 | 0.941 |
|  | MU | *r* | 0.004 | 0.037 | 0.035 | 0.638 | 0.059 | 0.612 |
|  |  | *p* | 0.992 | 0.925 | 0.949 | 0.679 | 0.932 | 0.797 |
|  | NA | *r* | 0.163 | 0.106 | 0.119 | 0.160 | 0.529 | 0.086 |
|  |  | *p* | 0.936 | 0.925 | 0.949 | 0.900 | 0.695 | 0.941 |
|  | NMDA | *r* | 0.238 | 0.257 | 0.203 | 0.090 | 0.505 | 0.052 |
|  |  | *p* | 0.855 | 0.909 | 0.901 | 0.900 | 0.695 | 0.941 |
|  | SERT_DAS | *r* | 0.228 | 0.193 | 0.265 | 0.114 | 0.561 | 0.029 |
|  |  | *p* | 0.855 | 0.925 | 0.863 | 0.900 | 0.695 | 0.941 |
|  | SERT_MADAM | *r* | 0.223 | 0.222 | 0.246 | 0.169 | 0.450 | 0.110 |
|  |  | *p* | 0.855 | 0.925 | 0.863 | 0.900 | 0.695 | 0.941 |
|  | VACh | *r* | 0.657 | 0.520 | 0.616 | 0.212 | 0.102 | 0.170 |
|  |  | *p* | 0.197 | 0.389 | 0.279 | 0.900 | 0.932 | 0.941 |

The correction was applied across 18 neurotransmitters within each gradient, and separately for each hemisphere, resting-state and dataset. **p*<0.05, ***p*<0.01, ****p*<0.001

**Table S10.** Voxel-wise Pearson correction coefficients between gradients and neurotransmitter templates

|  | **D1 & Second-order gradient_L** | **D1 & Second-order gradient_R** | **5HT 1A & Third-order gradient_L** | **5HT 1A & Third-order gradient_R** | **D2 & Third-order gradient_L** | **D2 & Third-order gradient_R** | **DAT & Fourth-order gradient_L** | **DAT & Fourth-order gradient_R** |
| --- | --- | --- | --- | --- | --- | --- | --- | --- |
| **HBS** | 0.595 | 0.523 | 0.470 | 0.500 | 0.301 | 0.112 | 0.557 | 0.393 |
| **MIND-Set** | 0.616 | 0.470 | 0.586 | 0.434 | 0.460 | 0.515 | 0.467 | 0.382 |

Resting-state 1 for each datasets is used as an example. L, Left; R, Right.

**Table S11**. Correlations coefficients (Fisher r-to-z transformed, absolute values, with FDR correction) between gradient and neurotransmitter layouts, shown for TSM orders 2 and 4.

|  |  |  | **HBS**  **TSM order 2** | **HBS**  **TSM order 4** | **MIND-Set**  **TSM order 2** | **MIND-Set**  **TSM order 4** |
| --- | --- | --- | --- | --- | --- | --- |
| **Zeroth-order gradient_Left** | 5HT1A | *z* | 0.761 | 1.097 | 0.662 | 0.950 |
|  |  | *p* | 0.932 | 0.747 | 0.897 | 0.859 |
|  | 5HT1B | *z* | 0.133 | 0.319 | 0.182 | 0.377 |
|  |  | *p* | 0.957 | 0.895 | 0.986 | 0.859 |
|  | 5HT2A | *z* | 0.459 | 0.022 | 0.388 | 0.007 |
|  |  | *p* | 0.932 | 0.974 | 0.897 | 0.988 |
|  | 5HT4 | *z* | 0.591 | 0.716 | 0.522 | 0.644 |
|  |  | *p* | 0.932 | 0.895 | 0.897 | 0.859 |
|  | CB1 | *z* | 0.510 | 0.694 | 0.496 | 0.663 |
|  |  | *p* | 0.932 | 0.895 | 0.897 | 0.859 |
|  | D1 | *z* | 0.330 | 0.289 | 0.364 | 0.300 |
|  |  | *p* | 0.936 | 0.895 | 0.897 | 0.859 |
|  | D2 | *z* | 0.497 | 0.775 | 0.430 | 0.673 |
|  |  | *p* | 0.932 | 0.895 | 0.897 | 0.859 |
|  | DAT | *z* | 0.077 | 0.189 | 0.011 | 0.266 |
|  |  | *p* | 0.957 | 0.920 | 0.994 | 0.859 |
|  | FDOPA | *z* | 0.924 | 0.532 | 0.975 | 0.554 |
|  |  | *p* | 0.932 | 0.895 | 0.897 | 0.859 |
|  | GABAa | *z* | 0.547 | 0.550 | 0.473 | 0.471 |
|  |  | *p* | 0.932 | 0.895 | 0.897 | 0.859 |
|  | KappaOp | *z* | 1.069 | 0.534 | 1.104 | 0.570 |
|  |  | *p* | 0.932 | 0.895 | 0.897 | 0.859 |
|  | mGluR5 | *z* | 0.643 | 0.582 | 0.571 | 0.510 |
|  |  | *p* | 0.932 | 0.895 | 0.897 | 0.859 |
|  | MU | *z* | 0.778 | 0.310 | 0.812 | 0.332 |
|  |  | *p* | 0.932 | 0.895 | 0.897 | 0.859 |
|  | NA | *z* | 0.878 | 0.014 | 0.849 | 0.041 |
|  |  | *p* | 0.932 | 0.974 | 0.897 | 0.987 |
|  | NMDA | *z* | 0.144 | 0.089 | 0.129 | 0.047 |
|  |  | *p* | 0.957 | 0.943 | 0.986 | 0.987 |
|  | SERT_DAS | *z* | 0.853 | 0.696 | 0.883 | 0.726 |
|  |  | *p* | 0.932 | 0.895 | 0.897 | 0.859 |
|  | SERT_MADAM | *z* | 0.389 | 0.424 | 0.432 | 0.440 |
|  |  | *p* | 0.936 | 0.895 | 0.897 | 0.859 |
|  | VACh | *z* | 0.435 | 0.089 | 0.360 | 0.043 |
|  |  | *p* | 0.932 | 0.943 | 0.897 | 0.987 |
| **Zeroth-order gradient_Right** | 5HT1A | *z* | 0.800 | 1.094 | 0.616 | 0.848 |
|  |  | *p* | 0.743 | 0.809 | 0.996 | 0.772 |
|  | 5HT1B | *z* | 0.243 | 0.140 | 0.137 | 0.260 |
|  |  | *p* | 0.932 | 0.869 | 0.996 | 0.772 |
|  | 5HT2A | *z* | 0.416 | 0.121 | 0.287 | 0.130 |
|  |  | *p* | 0.932 | 0.869 | 0.996 | 0.847 |
|  | 5HT4 | *z* | 0.310 | 0.619 | 0.184 | 0.510 |
|  |  | *p* | 0.932 | 0.809 | 0.996 | 0.772 |
|  | CB1 | *z* | 0.358 | 0.574 | 0.312 | 0.549 |
|  |  | *p* | 0.932 | 0.809 | 0.996 | 0.772 |
|  | D1 | *z* | 0.248 | 0.218 | 0.277 | 0.259 |
|  |  | *p* | 0.932 | 0.869 | 0.996 | 0.772 |
|  | D2 | *z* | 0.528 | 0.409 | 0.442 | 0.280 |
|  |  | *p* | 0.932 | 0.809 | 0.996 | 0.772 |
|  | DAT | *z* | 0.125 | 0.198 | 0.013 | 0.326 |
|  |  | *p* | 0.955 | 0.869 | 0.996 | 0.772 |
|  | FDOPA | *z* | 0.897 | 0.652 | 0.901 | 0.715 |
|  |  | *p* | 0.743 | 0.809 | 0.847 | 0.772 |
|  | GABAa | *z* | 0.501 | 0.351 | 0.358 | 0.213 |
|  |  | *p* | 0.932 | 0.809 | 0.996 | 0.781 |
|  | KappaOp | *z* | 1.186 | 0.650 | 1.210 | 0.728 |
|  |  | *p* | 0.743 | 0.809 | 0.847 | 0.772 |
|  | mGluR5 | *z* | 0.556 | 0.532 | 0.413 | 0.412 |
|  |  | *p* | 0.932 | 0.809 | 0.996 | 0.772 |
|  | MU | *z* | 0.796 | 0.339 | 0.839 | 0.403 |
|  |  | *p* | 0.743 | 0.809 | 0.847 | 0.772 |
|  | NA | *z* | 0.798 | 0.351 | 0.670 | 0.338 |
|  |  | *p* | 0.743 | 0.809 | 0.996 | 0.772 |
|  | NMDA | *z* | 0.404 | 0.321 | 0.452 | 0.409 |
|  |  | *p* | 0.932 | 0.809 | 0.996 | 0.772 |
|  | SERT_DAS | *z* | 0.813 | 0.738 | 0.963 | 0.848 |
|  |  | *p* | 0.743 | 0.809 | 0.847 | 0.772 |
|  | SERT_MADAM | *z* | 0.916 | 0.639 | 1.013 | 0.690 |
|  |  | *p* | 0.743 | 0.809 | 0.847 | 0.772 |
|  | VACh | *z* | 0.511 | 0.068 | 0.385 | 0.029 |
|  |  | *p* | 0.932 | 0.901 | 0.996 | 0.956 |
| **First-order gradient_Left** | 5HT1A | *z* | 0.085 | 0.518 | 0.586 | 0.003 |
|  |  | *p* | 0.992 | 0.925 | 0.859 | 0.996 |
|  | 5HT1B | *z* | 0.271 | 0.511 | 0.512 | 0.771 |
|  |  | *p* | 0.992 | 0.925 | 0.859 | 0.996 |
|  | 5HT2A | *z* | 0.308 | 0.008 | 0.718 | 0.080 |
|  |  | *p* | 0.992 | 0.995 | 0.859 | 0.996 |
|  | 5HT4 | *z* | 0.073 | 0.429 | 0.445 | 0.070 |
|  |  | *p* | 0.992 | 0.946 | 0.859 | 0.996 |
|  | CB1 | *z* | 0.154 | 0.723 | 0.240 | 0.442 |
|  |  | *p* | 0.992 | 0.925 | 0.859 | 0.996 |
|  | D1 | *z* | 0.030 | 0.617 | 0.088 | 0.648 |
|  |  | *p* | 0.992 | 0.925 | 0.871 | 0.996 |
|  | D2 | *z* | 0.148 | 0.386 | 0.297 | 0.109 |
|  |  | *p* | 0.992 | 0.946 | 0.859 | 0.996 |
|  | DAT | *z* | 0.196 | 0.137 | 0.214 | 0.109 |
|  |  | *p* | 0.992 | 0.970 | 0.859 | 0.996 |
|  | FDOPA | *z* | 0.073 | 0.512 | 0.078 | 0.497 |
|  |  | *p* | 0.992 | 0.925 | 0.871 | 0.996 |
|  | GABAa | *z* | 0.036 | 0.635 | 0.443 | 0.205 |
|  |  | *p* | 0.992 | 0.925 | 0.859 | 0.996 |
|  | KappaOp | *z* | 0.419 | 1.342 | 0.232 | 1.459 |
|  |  | *p* | 0.794 | 0.274 | 0.859 | 0.109 |
|  | mGluR5 | *z* | 0.003 | 0.742 | 0.388 | 0.308 |
|  |  | *p* | 0.992 | 0.925 | 0.859 | 0.996 |
|  | MU | *z* | 0.536 | 1.193 | 0.379 | 1.403 |
|  |  | *p* | 0.794 | 0.274 | 0.859 | 0.109 |
|  | NA | *z* | 0.099 | 0.285 | 0.123 | 0.224 |
|  |  | *p* | 0.992 | 0.946 | 0.871 | 0.996 |
|  | NMDA | *z* | 0.020 | 0.121 | 0.118 | 0.040 |
|  |  | *p* | 0.992 | 0.970 | 0.871 | 0.996 |
|  | SERT_DAS | *z* | 0.413 | 1.248 | 0.265 | 1.068 |
|  |  | *p* | 0.794 | 0.274 | 0.859 | 0.402 |
|  | SERT_MADAM | *z* | 0.637 | 1.263 | 0.689 | 1.260 |
|  |  | *p* | 0.794 | 0.274 | 0.859 | 0.206 |
|  | VACh | *z* | 0.079 | 0.289 | 0.558 | 0.630 |
|  |  | *p* | 0.992 | 0.946 | 0.859 | 0.996 |
| **First-order gradient_Right** | 5HT1A | *z* | 0.118 | 0.419 | 0.893 | 0.540 |
|  |  | *p* | 0.949 | 0.994 | 0.773 | 0.746 |
|  | 5HT1B | *z* | 0.294 | 0.350 | 0.809 | 0.734 |
|  |  | *p* | 0.949 | 0.994 | 0.773 | 0.746 |
|  | 5HT2A | *z* | 0.241 | 0.019 | 0.804 | 0.019 |
|  |  | *p* | 0.949 | 0.994 | 0.773 | 0.976 |
|  | 5HT4 | *z* | 0.427 | 0.260 | 0.963 | 0.372 |
|  |  | *p* | 0.949 | 0.994 | 0.773 | 0.746 |
|  | CB1 | *z* | 0.204 | 0.646 | 0.332 | 0.102 |
|  |  | *p* | 0.949 | 0.994 | 0.856 | 0.935 |
|  | D1 | *z* | 0.039 | 0.701 | 0.105 | 0.486 |
|  |  | *p* | 0.949 | 0.994 | 0.905 | 0.746 |
|  | D2 | *z* | 0.323 | 0.009 | 0.287 | 0.701 |
|  |  | *p* | 0.949 | 0.994 | 0.856 | 0.746 |
|  | DAT | *z* | 0.136 | 0.065 | 0.457 | 0.362 |
|  |  | *p* | 0.949 | 0.994 | 0.856 | 0.746 |
|  | FDOPA | *z* | 0.029 | 0.944 | 0.185 | 0.426 |
|  |  | *p* | 0.949 | 0.852 | 0.859 | 0.746 |
|  | GABAa | *z* | 0.097 | 0.251 | 0.762 | 0.505 |
|  |  | *p* | 0.949 | 0.994 | 0.773 | 0.746 |
|  | KappaOp | *z* | 0.534 | 1.548 | 0.063 | 0.602 |
|  |  | *p* | 0.845 | 0.130 | 0.910 | 0.746 |
|  | mGluR5 | *z* | 0.128 | 0.567 | 0.771 | 0.249 |
|  |  | *p* | 0.949 | 0.994 | 0.773 | 0.798 |
|  | MU | *z* | 0.668 | 1.415 | 0.231 | 0.770 |
|  |  | *p* | 0.798 | 0.174 | 0.859 | 0.746 |
|  | NA | *z* | 0.180 | 0.166 | 0.404 | 0.299 |
|  |  | *p* | 0.949 | 0.994 | 0.856 | 0.792 |
|  | NMDA | *z* | 0.512 | 0.303 | 0.269 | 0.366 |
|  |  | *p* | 0.845 | 0.994 | 0.856 | 0.746 |
|  | SERT_DAS | *z* | 0.697 | 1.379 | 0.468 | 0.622 |
|  |  | *p* | 0.798 | 0.174 | 0.856 | 0.746 |
|  | SERT_MADAM | *z* | 0.753 | 1.629 | 0.337 | 0.539 |
|  |  | *p* | 0.798 | 0.130 | 0.856 | 0.746 |
|  | VACh | *z* | 0.047 | 0.422 | 0.623 | 0.826 |
|  |  | *p* | 0.949 | 0.994 | 0.856 | 0.746 |
| **Second-order gradient_Left** | 5HT1A | *z* | 0.340 | 0.321 | 0.163 | 0.330 |
|  |  | *p* | 0.825 | 0.911 | 0.895 | 0.758 |
|  | 5HT1B | *z* | 0.565 | 0.585 | 0.628 | 0.563 |
|  |  | *p* | 0.825 | 0.757 | 0.815 | 0.557 |
|  | 5HT2A | *z* | 0.139 | 0.505 | 0.344 | 0.936 |
|  |  | *p* | 0.829 | 0.757 | 0.815 | 0.400 |
|  | 5HT4 | *z* | 0.270 | 0.147 | 0.503 | 0.251 |
|  |  | *p* | 0.825 | 0.911 | 0.815 | 0.761 |
|  | CB1 | *z* | 0.354 | 0.230 | 0.555 | 0.336 |
|  |  | *p* | 0.825 | 0.911 | 0.815 | 0.758 |
|  | D1 | *z* | 1.629 | 0.833 | 1.793 | 0.806 |
|  |  | *p* | 0.076 | 0.757 | 0.066 | 0.462 |
|  | D2 | *z* | 0.531 | 0.513 | 0.425 | 0.568 |
|  |  | *p* | 0.825 | 0.757 | 0.815 | 0.557 |
|  | DAT | *z* | 0.223 | 0.153 | 0.083 | 0.054 |
|  |  | *p* | 0.825 | 0.911 | 0.918 | 0.908 |
|  | FDOPA | *z* | 0.702 | 0.687 | 0.775 | 0.738 |
|  |  | *p* | 0.825 | 0.757 | 0.815 | 0.463 |
|  | GABAa | *z* | 0.157 | 0.018 | 0.385 | 0.172 |
|  |  | *p* | 0.829 | 0.972 | 0.815 | 0.807 |
|  | KappaOp | *z* | 0.521 | 0.293 | 0.658 | 0.249 |
|  |  | *p* | 0.825 | 0.911 | 0.815 | 0.761 |
|  | mGluR5 | *z* | 0.267 | 0.088 | 0.494 | 0.199 |
|  |  | *p* | 0.825 | 0.912 | 0.815 | 0.803 |
|  | MU | *z* | 0.702 | 0.331 | 0.840 | 0.308 |
|  |  | *p* | 0.825 | 0.911 | 0.815 | 0.758 |
|  | NA | *z* | 0.514 | 0.124 | 0.758 | 0.506 |
|  |  | *p* | 0.825 | 0.911 | 0.815 | 0.559 |
|  | NMDA | *z* | 1.568 | 0.483 | 1.861 | 0.581 |
|  |  | *p* | 0.076 | 0.757 | 0.066 | 0.557 |
|  | SERT_DAS | *z* | 0.241 | 0.263 | 0.158 | 0.311 |
|  |  | *p* | 0.825 | 0.911 | 0.895 | 0.758 |
|  | SERT_MADAM | *z* | 0.294 | 0.177 | 0.403 | 0.145 |
|  |  | *p* | 0.825 | 0.911 | 0.815 | 0.808 |
|  | VACh | *z* | 0.435 | 0.526 | 0.333 | 0.535 |
|  |  | *p* | 0.825 | 0.757 | 0.815 | 0.557 |
| **Second-order gradient_Right** | 5HT1A | *z* | 0.445 | 0.503 | 0.555 | 1.159 |
|  |  | *p* | 0.800 | 0.821 | 0.852 | 0.841 |
|  | 5HT1B | *z* | 0.532 | 0.365 | 0.105 | 0.057 |
|  |  | *p* | 0.800 | 0.821 | 0.987 | 0.928 |
|  | 5HT2A | *z* | 0.075 | 0.596 | 1.278 | 0.440 |
|  |  | *p* | 0.903 | 0.821 | 0.416 | 0.846 |
|  | 5HT4 | *z* | 0.148 | 0.144 | 1.145 | 1.046 |
|  |  | *p* | 0.903 | 0.826 | 0.416 | 0.841 |
|  | CB1 | *z* | 0.190 | 0.068 | 0.775 | 0.923 |
|  |  | *p* | 0.903 | 0.903 | 0.625 | 0.841 |
|  | D1 | *z* | 0.884 | 0.675 | 0.921 | 0.561 |
|  |  | *p* | 0.408 | 0.821 | 0.600 | 0.846 |
|  | D2 | *z* | 1.029 | 0.700 | 0.046 | 0.433 |
|  |  | *p* | 0.309 | 0.821 | 0.987 | 0.846 |
|  | DAT | *z* | 0.445 | 0.269 | 0.358 | 0.279 |
|  |  | *p* | 0.800 | 0.821 | 0.955 | 0.888 |
|  | FDOPA | *z* | 0.360 | 0.317 | 0.726 | 0.778 |
|  |  | *p* | 0.898 | 0.821 | 0.625 | 0.846 |
|  | GABAa | *z* | 0.161 | 0.293 | 1.019 | 0.867 |
|  |  | *p* | 0.903 | 0.821 | 0.454 | 0.841 |
|  | KappaOp | *z* | 0.087 | 0.269 | 0.737 | 0.668 |
|  |  | *p* | 0.903 | 0.821 | 0.625 | 0.846 |
|  | mGluR5 | *z* | 0.046 | 0.191 | 1.162 | 0.995 |
|  |  | *p* | 0.903 | 0.826 | 0.416 | 0.841 |
|  | MU | *z* | 0.270 | 0.329 | 0.809 | 0.435 |
|  |  | *p* | 0.903 | 0.821 | 0.625 | 0.846 |
|  | NA | *z* | 0.159 | 0.198 | 1.023 | 0.055 |
|  |  | *p* | 0.903 | 0.826 | 0.454 | 0.928 |
|  | NMDA | *z* | 1.055 | 0.198 | 0.776 | 0.096 |
|  |  | *p* | 0.309 | 0.826 | 0.625 | 0.928 |
|  | SERT_DAS | *z* | 0.280 | 0.144 | 0.022 | 0.347 |
|  |  | *p* | 0.903 | 0.826 | 0.987 | 0.874 |
|  | SERT_MADAM | *z* | 0.047 | 0.051 | 0.452 | 0.563 |
|  |  | *p* | 0.903 | 0.903 | 0.923 | 0.846 |
|  | VACh | *z* | 0.627 | 0.452 | 0.181 | 0.060 |
|  |  | *p* | 0.780 | 0.821 | 0.987 | 0.928 |
| **Third-order gradient_Left** | 5HT1A | *z* | 1.447 | 1.023 | 1.558 | 1.866 ** |
|  |  | *p* | 0.235 | 0.243 | 0.110 | 0.002 |
|  | 5HT1B | *z* | 0.803 | 0.035 | 0.458 | 0.045 |
|  |  | *p* | 0.653 | 0.938 | 0.785 | 0.965 |
|  | 5HT2A | *z* | 0.713 | 0.103 | 0.841 | 0.102 |
|  |  | *p* | 0.653 | 0.938 | 0.541 | 0.965 |
|  | 5HT4 | *z* | 0.698 | 0.458 | 1.003 | 0.820 |
|  |  | *p* | 0.653 | 0.938 | 0.411 | 0.612 |
|  | CB1 | *z* | 0.025 | 0.285 | 0.185 | 0.604 |
|  |  | *p* | 0.990 | 0.938 | 0.877 | 0.921 |
|  | D1 | *z* | 0.293 | 0.089 | 0.143 | 0.183 |
|  |  | *p* | 0.863 | 0.938 | 0.877 | 0.965 |
|  | D2 | *z* | 1.579 | 1.042 | 1.484 | 1.533 * |
|  |  | *p* | 0.235 | 0.243 | 0.110 | 0.028 |
|  | DAT | *z* | 0.945 | 0.150 | 1.237 | 0.301 |
|  |  | *p* | 0.653 | 0.938 | 0.286 | 0.965 |
|  | FDOPA | *z* | 0.007 | 0.107 | 0.089 | 0.245 |
|  |  | *p* | 0.990 | 0.938 | 0.877 | 0.965 |
|  | GABAa | *z* | 0.790 | 0.285 | 1.165 | 0.873 |
|  |  | *p* | 0.653 | 0.938 | 0.305 | 0.612 |
|  | KappaOp | *z* | 0.174 | 0.036 | 0.439 | 0.294 |
|  |  | *p* | 0.963 | 0.938 | 0.785 | 0.965 |
|  | mGluR5 | *z* | 0.762 | 0.271 | 1.133 | 0.860 |
|  |  | *p* | 0.653 | 0.938 | 0.305 | 0.612 |
|  | MU | *z* | 0.069 | 0.149 | 0.359 | 0.187 |
|  |  | *p* | 0.990 | 0.938 | 0.831 | 0.965 |
|  | NA | *z* | 0.306 | 0.323 | 0.571 | 0.061 |
|  |  | *p* | 0.863 | 0.938 | 0.782 | 0.965 |
|  | NMDA | *z* | 0.397 | 0.070 | 0.201 | 0.260 |
|  |  | *p* | 0.821 | 0.938 | 0.877 | 0.965 |
|  | SERT_DAS | *z* | 0.090 | 0.237 | 0.236 | 0.412 |
|  |  | *p* | 0.990 | 0.938 | 0.877 | 0.965 |
|  | SERT_MADAM | *z* | 0.436 | 0.055 | 0.090 | 0.279 |
|  |  | *p* | 0.821 | 0.938 | 0.877 | 0.965 |
|  | VACh | *z* | 2.086 | 0.499 | 1.469 | 0.378 |
|  |  | *p* | 0.110 | 0.938 | 0.110 | 0.965 |
| **Third-order gradient_Right** | 5HT1A | *z* | 1.566 | 1.104 | 0.997 | 1.644 * |
|  |  | *p* | 0.280 | 0.146 | 0.345 | 0.029 |
|  | 5HT1B | *z* | 0.829 | 0.116 | 0.727 | 0.255 |
|  |  | *p* | 0.590 | 0.975 | 0.479 | 0.905 |
|  | 5HT2A | *z* | 1.172 | 0.061 | 0.431 | 0.106 |
|  |  | *p* | 0.428 | 0.975 | 0.609 | 0.947 |
|  | 5HT4 | *z* | 1.047 | 0.598 | 0.278 | 0.674 |
|  |  | *p* | 0.428 | 0.975 | 0.681 | 0.879 |
|  | CB1 | *z* | 0.300 | 0.348 | 0.042 | 0.524 |
|  |  | *p* | 0.854 | 0.975 | 0.935 | 0.879 |
|  | D1 | *z* | 0.045 | 0.038 | 0.314 | 0.208 |
|  |  | *p* | 0.943 | 0.975 | 0.681 | 0.905 |
|  | D2 | *z* | 0.749 | 0.611 | 1.845 * | 0.712 |
|  |  | *p* | 0.603 | 0.975 | 0.029 | 0.879 |
|  | DAT | *z* | 1.019 | 0.039 | 1.107 | 0.337 |
|  |  | *p* | 0.428 | 0.975 | 0.323 | 0.898 |
|  | FDOPA | *z* | 0.363 | 0.278 | 0.051 | 0.432 |
|  |  | *p* | 0.836 | 0.975 | 0.935 | 0.879 |
|  | GABAa | *z* | 1.398 | 0.385 | 0.718 | 0.784 |
|  |  | *p* | 0.280 | 0.975 | 0.479 | 0.879 |
|  | KappaOp | *z* | 0.541 | 0.278 | 0.572 | 0.545 |
|  |  | *p* | 0.764 | 0.975 | 0.522 | 0.879 |
|  | mGluR5 | *z* | 1.444 | 0.440 | 0.639 | 0.982 |
|  |  | *p* | 0.280 | 0.975 | 0.522 | 0.775 |
|  | MU | *z* | 0.412 | 0.020 | 0.444 | 0.352 |
|  |  | *p* | 0.836 | 0.975 | 0.609 | 0.898 |
|  | NA | *z* | 1.030 | 0.512 | 0.778 | 0.184 |
|  |  | *p* | 0.428 | 0.975 | 0.479 | 0.905 |
|  | NMDA | *z* | 0.085 | 0.193 | 0.579 | 0.058 |
|  |  | *p* | 0.943 | 0.975 | 0.522 | 0.947 |
|  | SERT_DAS | *z* | 0.120 | 0.228 | 0.278 | 0.481 |
|  |  | *p* | 0.943 | 0.975 | 0.681 | 0.879 |
|  | SERT_MADAM | *z* | 0.175 | 0.229 | 0.411 | 0.602 |
|  |  | *p* | 0.943 | 0.975 | 0.609 | 0.879 |
|  | VACh | *z* | 1.244 | 0.406 | 1.450 | 0.272 |
|  |  | *p* | 0.428 | 0.975 | 0.124 | 0.905 |
| **Fourth-order gradient_Left** | 5HT1A | *z* | 1.273 | 0.600 | 1.154 | 0.116 |
|  |  | *p* | 0.355 | 0.679 | 0.288 | 0.938 |
|  | 5HT1B | *z* | 0.212 | 0.073 | 0.205 | 0.207 |
|  |  | *p* | 0.914 | 0.974 | 0.891 | 0.930 |
|  | 5HT2A | *z* | 1.113 | 0.584 | 0.906 | 0.425 |
|  |  | *p* | 0.386 | 0.679 | 0.378 | 0.926 |
|  | 5HT4 | *z* | 1.410 | 0.752 | 1.087 | 0.286 |
|  |  | *p* | 0.304 | 0.679 | 0.288 | 0.926 |
|  | CB1 | *z* | 0.417 | 0.650 | 0.355 | 0.251 |
|  |  | *p* | 0.746 | 0.679 | 0.773 | 0.926 |
|  | D1 | *z* | 0.051 | 0.513 | 0.080 | 0.315 |
|  |  | *p* | 0.936 | 0.679 | 0.929 | 0.926 |
|  | D2 | *z* | 0.902 | 0.446 | 0.892 | 0.066 |
|  |  | *p* | 0.461 | 0.703 | 0.378 | 0.938 |
|  | DAT | *z* | 1.122 | 1.167 | 1.107 | 1.182 |
|  |  | *p* | 0.386 | 0.361 | 0.288 | 0.292 |
|  | FDOPA | *z* | 0.189 | 0.316 | 0.067 | 0.060 |
|  |  | *p* | 0.914 | 0.766 | 0.929 | 0.938 |
|  | GABAa | *z* | 1.673 | 1.427 | 1.285 | 0.673 |
|  |  | *p* | 0.211 | 0.189 | 0.288 | 0.629 |
|  | KappaOp | *z* | 0.527 | 0.372 | 0.432 | 0.161 |
|  |  | *p* | 0.728 | 0.740 | 0.773 | 0.938 |
|  | mGluR5 | *z* | 1.565 | 1.368 | 1.168 | 0.650 |
|  |  | *p* | 0.211 | 0.189 | 0.288 | 0.629 |
|  | MU | *z* | 0.462 | 0.501 | 0.369 | 0.363 |
|  |  | *p* | 0.746 | 0.679 | 0.773 | 0.926 |
|  | NA | *z* | 0.805 | 0.807 | 0.652 | 0.778 |
|  |  | *p* | 0.490 | 0.679 | 0.572 | 0.629 |
|  | NMDA | *z* | 0.056 | 0.799 | 0.046 | 0.623 |
|  |  | *p* | 0.936 | 0.679 | 0.929 | 0.629 |
|  | SERT_DAS | *z* | 0.202 | 0.190 | 0.254 | 0.015 |
|  |  | *p* | 0.914 | 0.883 | 0.869 | 0.971 |
|  | SERT_MADAM | *z* | 0.071 | 0.506 | 0.112 | 0.329 |
|  |  | *p* | 0.936 | 0.679 | 0.929 | 0.926 |
|  | VACh | *z* | 0.951 | 0.019 | 0.876 | 0.104 |
|  |  | *p* | 0.461 | 0.978 | 0.378 | 0.938 |
| **Fourth-order gradient_Right** | 5HT1A | *z* | 1.220 | 0.512 | 1.068 | 0.011 |
|  |  | *p* | 0.425 | 0.721 | 0.353 | 0.996 |
|  | 5HT1B | *z* | 0.309 | 0.146 | 0.326 | 0.240 |
|  |  | *p* | 0.843 | 0.879 | 0.810 | 0.843 |
|  | 5HT2A | *z* | 1.434 | 0.681 | 1.183 | 0.612 |
|  |  | *p* | 0.256 | 0.721 | 0.309 | 0.537 |
|  | 5HT4 | *z* | 1.108 | 0.739 | 0.920 | 0.208 |
|  |  | *p* | 0.467 | 0.721 | 0.447 | 0.843 |
|  | CB1 | *z* | 0.561 | 0.754 | 0.427 | 0.271 |
|  |  | *p* | 0.699 | 0.721 | 0.766 | 0.843 |
|  | D1 | *z* | 0.287 | 0.620 | 0.231 | 0.444 |
|  |  | *p* | 0.843 | 0.721 | 0.868 | 0.717 |
|  | D2 | *z* | 0.559 | 0.254 | 0.621 | 0.002 |
|  |  | *p* | 0.699 | 0.848 | 0.629 | 0.996 |
|  | DAT | *z* | 0.928 | 1.019 | 1.046 | 0.966 |
|  |  | *p* | 0.620 | 0.592 | 0.353 | 0.527 |
|  | FDOPA | *z* | 0.428 | 0.499 | 0.336 | 0.163 |
|  |  | *p* | 0.755 | 0.721 | 0.810 | 0.843 |
|  | GABAa | *z* | 1.889 | 1.396 | 1.516 | 0.661 |
|  |  | *p* | 0.115 | 0.283 | 0.145 | 0.537 |
|  | KappaOp | *z* | 0.716 | 0.383 | 0.711 | 0.108 |
|  |  | *p* | 0.678 | 0.721 | 0.590 | 0.865 |
|  | mGluR5 | *z* | 1.752 | 1.243 | 1.424 | 0.551 |
|  |  | *p* | 0.119 | 0.331 | 0.145 | 0.584 |
|  | MU | *z* | 0.631 | 0.452 | 0.668 | 0.295 |
|  |  | *p* | 0.699 | 0.721 | 0.590 | 0.843 |
|  | NA | *z* | 1.650 | 0.536 | 1.413 | 0.801 |
|  |  | *p* | 0.133 | 0.721 | 0.145 | 0.527 |
|  | NMDA | *z* | 0.204 | 0.641 | 0.154 | 0.737 |
|  |  | *p* | 0.876 | 0.721 | 0.904 | 0.527 |
|  | SERT_DAS | *z* | 0.070 | 0.083 | 0.056 | 0.195 |
|  |  | *p* | 0.946 | 0.898 | 0.944 | 0.843 |
|  | SERT_MADAM | *z* | 0.462 | 0.424 | 0.452 | 0.126 |
|  |  | *p* | 0.755 | 0.721 | 0.766 | 0.865 |
|  | VACh | *z* | 0.752 | 0.161 | 0.796 | 0.180 |
|  |  | *p* | 0.678 | 0.879 | 0.560 | 0.843 |
| **Fifth-order gradient_Left** | 5HT1A | *z* | 0.219 | 0.438 | 0.023 | 0.729 |
|  |  | *p* | 0.838 | 0.698 | 0.941 | 0.867 |
|  | 5HT1B | *z* | 0.698 | 0.337 | 0.531 | 0.480 |
|  |  | *p* | 0.454 | 0.698 | 0.523 | 0.879 |
|  | 5HT2A | *z* | 0.080 | 0.413 | 0.054 | 0.351 |
|  |  | *p* | 0.985 | 0.698 | 0.941 | 0.892 |
|  | 5HT4 | *z* | 0.162 | 0.822 | 0.047 | 1.203 |
|  |  | *p* | 0.838 | 0.256 | 0.941 | 0.179 |
|  | CB1 | *z* | 0.459 | 0.607 | 0.333 | 1.253 |
|  |  | *p* | 0.478 | 0.698 | 0.774 | 0.179 |
|  | D1 | *z* | 0.005 | 0.093 | 0.134 | 0.494 |
|  |  | *p* | 0.988 | 0.946 | 0.941 | 0.879 |
|  | D2 | *z* | 0.566 | 0.117 | 0.192 | 0.289 |
|  |  | *p* | 0.454 | 0.941 | 0.941 | 0.892 |
|  | DAT | *z* | 0.528 | 0.334 | 0.170 | 0.013 |
|  |  | *p* | 0.454 | 0.698 | 0.941 | 0.994 |
|  | FDOPA | *z* | 0.060 | 0.457 | 0.030 | 0.677 |
|  |  | *p* | 0.985 | 0.698 | 0.941 | 0.867 |
|  | GABAa | *z* | 0.167 | 0.348 | 0.016 | 0.958 |
|  |  | *p* | 0.838 | 0.698 | 0.941 | 0.563 |
|  | KappaOp | *z* | 0.074 | 0.116 | 0.088 | 0.491 |
|  |  | *p* | 0.985 | 0.941 | 0.941 | 0.879 |
|  | mGluR5 | *z* | 0.236 | 0.272 | 0.077 | 0.927 |
|  |  | *p* | 0.838 | 0.754 | 0.941 | 0.563 |
|  | MU | *z* | 0.158 | 0.235 | 0.026 | 0.437 |
|  |  | *p* | 0.838 | 0.801 | 0.941 | 0.879 |
|  | NA | *z* | 0.021 | 0.013 | 0.096 | 0.273 |
|  |  | *p* | 0.988 | 0.965 | 0.941 | 0.892 |
|  | NMDA | *z* | 0.032 | 0.317 | 0.149 | 0.517 |
|  |  | *p* | 0.988 | 0.698 | 0.941 | 0.879 |
|  | SERT_DAS | *z* | 0.391 | 0.048 | 0.558 | 0.671 |
|  |  | *p* | 0.511 | 0.965 | 0.523 | 0.867 |
|  | SERT_MADAM | *z* | 0.604 | 0.018 | 0.715 | 0.741 |
|  |  | *p* | 0.454 | 0.965 | 0.523 | 0.867 |
|  | VACh | *z* | 0.623 | 0.193 | 0.318 | 0.445 |
|  |  | *p* | 0.454 | 0.872 | 0.774 | 0.879 |
| **Fifth-order gradient_Right** | 5HT1A | *z* | 0.504 | 0.059 | 0.061 | 0.166 |
|  |  | *p* | 0.643 | 0.881 | 0.936 | 0.973 |
|  | 5HT1B | *z* | 1.208 | 0.637 | 0.078 | 0.045 |
|  |  | *p* | 0.216 | 0.196 | 0.936 | 0.973 |
|  | 5HT2A | *z* | 0.213 | 0.152 | 0.044 | 0.018 |
|  |  | *p* | 0.651 | 0.803 | 0.936 | 0.973 |
|  | 5HT4 | *z* | 0.159 | 0.307 | 0.029 | 0.273 |
|  |  | *p* | 0.727 | 0.556 | 0.936 | 0.973 |
|  | CB1 | *z* | 0.473 | 0.159 | 0.312 | 0.058 |
|  |  | *p* | 0.643 | 0.803 | 0.936 | 0.973 |
|  | D1 | *z* | 0.391 | 0.275 | 0.270 | 0.754 |
|  |  | *p* | 0.643 | 0.607 | 0.936 | 0.677 |
|  | D2 | *z* | 0.922 | 0.036 | 0.112 | 0.514 |
|  |  | *p* | 0.319 | 0.896 | 0.936 | 0.973 |
|  | DAT | *z* | 0.946 | 0.457 | 0.272 | 0.062 |
|  |  | *p* | 0.319 | 0.422 | 0.936 | 0.973 |
|  | FDOPA | *z* | 0.114 | 0.071 | 0.153 | 0.428 |
|  |  | *p* | 0.790 | 0.881 | 0.936 | 0.973 |
|  | GABAa | *z* | 0.330 | 0.166 | 0.026 | 0.035 |
|  |  | *p* | 0.643 | 0.803 | 0.936 | 0.973 |
|  | KappaOp | *z* | 0.268 | 0.415 | 0.554 | 0.765 |
|  |  | *p* | 0.643 | 0.427 | 0.761 | 0.677 |
|  | mGluR5 | *z* | 0.348 | 0.326 | 0.105 | 0.249 |
|  |  | *p* | 0.643 | 0.535 | 0.936 | 0.973 |
|  | MU | *z* | 0.275 | 0.566 | 0.843 | 1.070 |
|  |  | *p* | 0.643 | 0.228 | 0.200 | 0.247 |
|  | NA | *z* | 0.226 | 0.397 | 0.112 | 0.385 |
|  |  | *p* | 0.651 | 0.427 | 0.936 | 0.973 |
|  | NMDA | *z* | 0.286 | 0.080 | 0.101 | 0.190 |
|  |  | *p* | 0.643 | 0.881 | 0.936 | 0.973 |
|  | SERT_DAS | *z* | 0.253 | 0.120 | 0.039 | 0.366 |
|  |  | *p* | 0.643 | 0.846 | 0.936 | 0.973 |
|  | SERT_MADAM | *z* | 0.091 | 0.362 | 0.245 | 0.679 |
|  |  | *p* | 0.796 | 0.469 | 0.936 | 0.701 |
|  | VACh | *z* | 1.273 | 0.170 | 0.215 | 0.436 |
|  |  | *p* | 0.216 | 0.803 | 0.936 | 0.973 |

Trend-surface regression models (order 2: six coefficients, order 4: 12 coefficients) were applied to group-level gradient maps and the neurotransmitter PET/SPECT scans. Fisher’s r-to-z–transformed correlation coefficients were computed between the TSM coefficients representing gradient maps and those representing neurotransmitter maps. The p-value was estimated using a permutation test as the proportion of permuted z-values that were lower than the observed Fisher r-to-z–transformed correlation coefficients, with FDR correction applied. The correction was applied across 18 neurotransmitters within each gradient, and separately for each hemisphere, TSM order and dataset. **p*<0.05, ***p*<0.01.

**Table S12**. Correlations coefficients (Fisher r-to-z transformed, absolute values, with FDR correction) between gradient and neurotransmitter layouts, separately for participants with high and low head motion in the HBS sample (data is from resting-state 1)

|  |  |  | **High motion _Left** | **High motion _Right** | **Low motion _Left** | **Low motion _Right** |
| --- | --- | --- | --- | --- | --- | --- |
| **Zeroth-order gradient** | 5HT1A | *z* | 1.044 | 1.047 | 1.056 | 1.055 |
|  |  | *p* | 0.943 | 0.763 | 0.953 | 0.776 |
|  | 5HT1B | *z* | 0.313 | 0.122 | 0.310 | 0.114 |
|  |  | *p* | 0.943 | 0.998 | 0.953 | 0.996 |
|  | 5HT2A | *z* | 0.057 | 0.047 | 0.066 | 0.051 |
|  |  | *p* | 0.978 | 0.998 | 0.970 | 0.996 |
|  | 5HT4 | *z* | 0.876 | 0.620 | 0.887 | 0.618 |
|  |  | *p* | 0.943 | 0.763 | 0.953 | 0.776 |
|  | CB1 | *z* | 0.727 | 0.479 | 0.736 | 0.477 |
|  |  | *p* | 0.943 | 0.763 | 0.953 | 0.776 |
|  | D1 | *z* | 0.213 | 0.043 | 0.215 | 0.033 |
|  |  | *p* | 0.971 | 0.998 | 0.970 | 0.996 |
|  | D2 | *z* | 0.809 | 0.494 | 0.814 | 0.502 |
|  |  | *p* | 0.943 | 0.763 | 0.953 | 0.776 |
|  | DAT | *z* | 0.102 | 0.001 | 0.098 | 0.003 |
|  |  | *p* | 0.978 | 0.998 | 0.970 | 0.996 |
|  | FDOPA | *z* | 0.461 | 0.657 | 0.463 | 0.650 |
|  |  | *p* | 0.943 | 0.763 | 0.953 | 0.776 |
|  | GABAa | *z* | 0.566 | 0.347 | 0.576 | 0.351 |
|  |  | *p* | 0.943 | 0.889 | 0.953 | 0.872 |
|  | KappaOp | *z* | 0.730 | 1.025 | 0.723 | 1.012 |
|  |  | *p* | 0.943 | 0.763 | 0.953 | 0.776 |
|  | mGluR5 | *z* | 0.620 | 0.509 | 0.630 | 0.513 |
|  |  | *p* | 0.943 | 0.763 | 0.953 | 0.776 |
|  | MU | *z* | 0.385 | 0.476 | 0.378 | 0.468 |
|  |  | *p* | 0.943 | 0.763 | 0.953 | 0.776 |
|  | NA | *z* | 0.010 | 0.259 | 0.005 | 0.257 |
|  |  | *p* | 0.985 | 0.942 | 0.993 | 0.929 |
|  | NMDA | *z* | 0.152 | 0.499 | 0.148 | 0.502 |
|  |  | *p* | 0.978 | 0.763 | 0.970 | 0.776 |
|  | SERT_DAS | *z* | 0.689 | 0.722 | 0.681 | 0.722 |
|  |  | *p* | 0.943 | 0.763 | 0.953 | 0.776 |
|  | SERT_MADAM | *z* | 0.509 | 0.912 | 0.501 | 0.907 |
|  |  | *p* | 0.943 | 0.763 | 0.953 | 0.776 |
|  | VACh | *z* | 0.348 | 0.518 | 0.353 | 0.525 |
|  |  | *p* | 0.943 | 0.763 | 0.953 | 0.776 |
| **First-order gradient** | 5HT1A | *z* | 0.604 | 0.407 | 0.652 | 0.445 |
|  |  | *p* | 0.875 | 0.917 | 0.845 | 0.933 |
|  | 5HT1B | *z* | 0.420 | 0.344 | 0.421 | 0.331 |
|  |  | *p* | 0.875 | 0.917 | 0.845 | 0.933 |
|  | 5HT2A | *z* | 0.180 | 0.147 | 0.141 | 0.138 |
|  |  | *p* | 0.970 | 0.917 | 0.980 | 0.933 |
|  | 5HT4 | *z* | 0.526 | 0.158 | 0.593 | 0.194 |
|  |  | *p* | 0.875 | 0.917 | 0.845 | 0.933 |
|  | CB1 | *z* | 0.378 | 0.183 | 0.427 | 0.196 |
|  |  | *p* | 0.875 | 0.917 | 0.845 | 0.933 |
|  | D1 | *z* | 0.101 | 0.036 | 0.143 | 0.052 |
|  |  | *p* | 0.970 | 0.954 | 0.980 | 0.933 |
|  | D2 | *z* | 0.599 | 0.264 | 0.635 | 0.284 |
|  |  | *p* | 0.875 | 0.917 | 0.845 | 0.933 |
|  | DAT | *z* | 0.070 | 0.106 | 0.067 | 0.103 |
|  |  | *p* | 0.970 | 0.917 | 0.980 | 0.933 |
|  | FDOPA | *z* | 0.194 | 0.482 | 0.237 | 0.503 |
|  |  | *p* | 0.970 | 0.917 | 0.980 | 0.933 |
|  | GABAa | *z* | 0.416 | 0.180 | 0.453 | 0.186 |
|  |  | *p* | 0.875 | 0.917 | 0.845 | 0.933 |
|  | KappaOp | *z* | 0.996 | 1.300 | 1.005 | 1.380 |
|  |  | *p* | 0.828 | 0.248 | 0.845 | 0.234 |
|  | mGluR5 | *z* | 0.474 | 0.301 | 0.516 | 0.314 |
|  |  | *p* | 0.875 | 0.917 | 0.845 | 0.933 |
|  | MU | *z* | 0.747 | 1.199 | 0.734 | 1.189 |
|  |  | *p* | 0.828 | 0.275 | 0.845 | 0.331 |
|  | NA | *z* | 0.102 | 0.105 | 0.090 | 0.063 |
|  |  | *p* | 0.970 | 0.917 | 0.980 | 0.933 |
|  | NMDA | *z* | 0.020 | 0.425 | 0.003 | 0.436 |
|  |  | *p* | 0.976 | 0.917 | 0.998 | 0.933 |
|  | SERT_DAS | *z* | 0.813 | 0.936 | 0.778 | 0.925 |
|  |  | *p* | 0.828 | 0.653 | 0.845 | 0.738 |
|  | SERT_MADAM | *z* | 0.816 | 1.337 | 0.789 | 1.340 |
|  |  | *p* | 0.828 | 0.248 | 0.845 | 0.234 |
|  | VACh | *z* | 0.200 | 0.235 | 0.218 | 0.262 |
|  |  | *p* | 0.970 | 0.917 | 0.980 | 0.933 |
| **Second-order gradient** | 5HT1A | *z* | 0.207 | 0.411 | 0.308 | 0.436 |
|  |  | *p* | 0.899 | 0.790 | 0.794 | 0.818 |
|  | 5HT1B | *z* | 0.458 | 0.413 | 0.410 | 0.358 |
|  |  | *p* | 0.895 | 0.790 | 0.794 | 0.820 |
|  | 5HT2A | *z* | 0.592 | 0.472 | 0.635 | 0.589 |
|  |  | *p* | 0.895 | 0.790 | 0.794 | 0.818 |
|  | 5HT4 | *z* | 0.444 | 0.050 | 0.355 | 0.066 |
|  |  | *p* | 0.895 | 0.951 | 0.794 | 0.935 |
|  | CB1 | *z* | 0.392 | 0.031 | 0.315 | 0.035 |
|  |  | *p* | 0.895 | 0.951 | 0.794 | 0.935 |
|  | D1 | *z* | 1.943 * | 1.160 | 1.739 * | 1.232 |
|  |  | *p* | 0.014 | 0.353 | 0.032 | 0.259 |
|  | D2 | *z* | 0.303 | 0.520 | 0.398 | 0.494 |
|  |  | *p* | 0.899 | 0.790 | 0.794 | 0.818 |
|  | DAT | *z* | 0.076 | 0.294 | 0.023 | 0.178 |
|  |  | *p* | 0.910 | 0.790 | 0.968 | 0.847 |
|  | FDOPA | *z* | 1.066 | 0.364 | 0.946 | 0.322 |
|  |  | *p* | 0.586 | 0.790 | 0.606 | 0.839 |
|  | GABAa | *z* | 0.123 | 0.319 | 0.091 | 0.232 |
|  |  | *p* | 0.899 | 0.790 | 0.958 | 0.847 |
|  | KappaOp | *z* | 0.410 | 0.227 | 0.310 | 0.159 |
|  |  | *p* | 0.895 | 0.808 | 0.794 | 0.847 |
|  | mGluR5 | *z* | 0.207 | 0.310 | 0.167 | 0.245 |
|  |  | *p* | 0.899 | 0.790 | 0.924 | 0.847 |
|  | MU | *z* | 0.528 | 0.301 | 0.463 | 0.265 |
|  |  | *p* | 0.895 | 0.790 | 0.794 | 0.847 |
|  | NA | *z* | 0.161 | 0.086 | 0.229 | 0.035 |
|  |  | *p* | 0.899 | 0.951 | 0.874 | 0.935 |
|  | NMDA | *z* | 0.783 | 0.292 | 0.899 | 0.428 |
|  |  | *p* | 0.895 | 0.790 | 0.606 | 0.818 |
|  | SERT_DAS | *z* | 0.367 | 0.248 | 0.486 | 0.362 |
|  |  | *p* | 0.895 | 0.808 | 0.794 | 0.820 |
|  | SERT_MADAM | *z* | 0.151 | 0.092 | 0.070 | 0.172 |
|  |  | *p* | 0.899 | 0.951 | 0.958 | 0.847 |
|  | VACh | *z* | 0.393 | 0.465 | 0.424 | 0.435 |
|  |  | *p* | 0.895 | 0.790 | 0.794 | 0.818 |
| **Third-order gradient** | 5HT1A | *z* | 1.524 | 1.613 | 1.371 | 1.497 |
|  |  | *p* | 0.115 | 0.065 | 0.254 | 0.119 |
|  | 5HT1B | *z* | 0.048 | 0.053 | 0.087 | 0.036 |
|  |  | *p* | 0.951 | 0.951 | 0.981 | 0.965 |
|  | 5HT2A | *z* | 0.165 | 0.176 | 0.194 | 0.134 |
|  |  | *p* | 0.951 | 0.951 | 0.981 | 0.946 |
|  | 5HT4 | *z* | 1.061 | 1.085 | 1.107 | 1.018 |
|  |  | *p* | 0.493 | 0.676 | 0.389 | 0.842 |
|  | CB1 | *z* | 0.700 | 0.593 | 0.736 | 0.565 |
|  |  | *p* | 0.800 | 0.869 | 0.765 | 0.889 |
|  | D1 | *z* | 0.114 | 0.111 | 0.190 | 0.103 |
|  |  | *p* | 0.951 | 0.951 | 0.981 | 0.946 |
|  | D2 | *z* | 1.267 | 0.654 | 1.139 | 0.613 |
|  |  | *p* | 0.276 | 0.834 | 0.389 | 0.889 |
|  | DAT | *z* | 0.168 | 0.296 | 0.133 | 0.235 |
|  |  | *p* | 0.951 | 0.951 | 0.981 | 0.946 |
|  | FDOPA | *z* | 0.243 | 0.446 | 0.312 | 0.445 |
|  |  | *p* | 0.951 | 0.951 | 0.981 | 0.939 |
|  | GABAa | *z* | 0.822 | 0.685 | 0.795 | 0.605 |
|  |  | *p* | 0.688 | 0.834 | 0.765 | 0.889 |
|  | KappaOp | *z* | 0.361 | 0.670 | 0.392 | 0.669 |
|  |  | *p* | 0.951 | 0.834 | 0.981 | 0.889 |
|  | mGluR5 | *z* | 0.853 | 0.874 | 0.837 | 0.783 |
|  |  | *p* | 0.688 | 0.834 | 0.765 | 0.889 |
|  | MU | *z* | 0.075 | 0.209 | 0.109 | 0.203 |
|  |  | *p* | 0.951 | 0.951 | 0.981 | 0.946 |
|  | NA | *z* | 0.059 | 0.289 | 0.070 | 0.351 |
|  |  | *p* | 0.951 | 0.951 | 0.981 | 0.946 |
|  | NMDA | *z* | 0.046 | 0.128 | 0.013 | 0.165 |
|  |  | *p* | 0.951 | 0.951 | 0.985 | 0.946 |
|  | SERT_DAS | *z* | 0.357 | 0.268 | 0.332 | 0.290 |
|  |  | *p* | 0.951 | 0.951 | 0.981 | 0.946 |
|  | SERT_MADAM | *z* | 0.161 | 0.483 | 0.178 | 0.489 |
|  |  | *p* | 0.951 | 0.951 | 0.981 | 0.939 |
|  | VACh | *z* | 0.663 | 0.833 | 0.592 | 0.779 |
|  |  | *p* | 0.800 | 0.834 | 0.930 | 0.889 |
| **Fourth-order gradient** | 5HT1A | *z* | 0.494 | 0.235 | 0.561 | 0.489 |
|  |  | *p* | 0.675 | 0.754 | 0.668 | 0.638 |
|  | 5HT1B | *z* | 0.297 | 0.274 | 0.332 | 0.377 |
|  |  | *p* | 0.871 | 0.754 | 0.814 | 0.710 |
|  | 5HT2A | *z* | 0.616 | 0.879 | 0.544 | 0.720 |
|  |  | *p* | 0.647 | 0.327 | 0.668 | 0.619 |
|  | 5HT4 | *z* | 0.502 | 0.485 | 0.508 | 0.628 |
|  |  | *p* | 0.675 | 0.725 | 0.672 | 0.619 |
|  | CB1 | *z* | 0.229 | 0.331 | 0.213 | 0.371 |
|  |  | *p* | 0.876 | 0.754 | 0.864 | 0.710 |
|  | D1 | *z* | 0.024 | 0.249 | 0.076 | 0.111 |
|  |  | *p* | 0.972 | 0.754 | 0.916 | 0.920 |
|  | D2 | *z* | 0.525 | 0.325 | 0.617 | 0.568 |
|  |  | *p* | 0.675 | 0.754 | 0.668 | 0.638 |
|  | DAT | *z* | 1.474 | 0.993 | 1.549 * | 1.270 |
|  |  | *p* | 0.076 | 0.327 | 0.038 | 0.204 |
|  | FDOPA | *z* | 0.144 | 0.026 | 0.185 | 0.025 |
|  |  | *p* | 0.876 | 0.958 | 0.864 | 0.965 |
|  | GABAa | *z* | 1.037 | 1.002 | 1.070 | 1.362 |
|  |  | *p* | 0.405 | 0.327 | 0.344 | 0.204 |
|  | KappaOp | *z* | 0.172 | 0.192 | 0.154 | 0.093 |
|  |  | *p* | 0.876 | 0.780 | 0.864 | 0.920 |
|  | mGluR5 | *z* | 0.980 | 0.805 | 1.012 | 1.126 |
|  |  | *p* | 0.405 | 0.327 | 0.344 | 0.253 |
|  | MU | *z* | 0.198 | 0.158 | 0.195 | 0.135 |
|  |  | *p* | 0.876 | 0.800 | 0.864 | 0.920 |
|  | NA | *z* | 0.885 | 0.833 | 0.830 | 0.677 |
|  |  | *p* | 0.405 | 0.327 | 0.451 | 0.619 |
|  | NMDA | *z* | 0.642 | 0.898 | 0.573 | 0.693 |
|  |  | *p* | 0.647 | 0.327 | 0.668 | 0.619 |
|  | SERT_DAS | *z* | 0.389 | 0.625 | 0.333 | 0.485 |
|  |  | *p* | 0.748 | 0.544 | 0.814 | 0.638 |
|  | SERT_MADAM | *z* | 0.429 | 0.333 | 0.415 | 0.214 |
|  |  | *p* | 0.748 | 0.754 | 0.793 | 0.908 |
|  | VACh | *z* | 0.792 | 0.400 | 0.896 | 0.676 |
|  |  | *p* | 0.486 | 0.754 | 0.429 | 0.619 |
| **Fifth-order gradient** | 5HT1A | *z* | 0.132 | 0.125 | 0.251 | 0.038 |
|  |  | *p* | 0.973 | 0.881 | 0.842 | 0.953 |
|  | 5HT1B | *z* | 0.855 | 1.141 | 0.738 | 1.156 |
|  |  | *p* | 0.159 | 0.223 | 0.251 | 0.191 |
|  | 5HT2A | *z* | 0.389 | 0.297 | 0.395 | 0.462 |
|  |  | *p* | 0.763 | 0.881 | 0.835 | 0.737 |
|  | 5HT4 | *z* | 0.337 | 0.205 | 0.373 | 0.309 |
|  |  | *p* | 0.889 | 0.881 | 0.835 | 0.883 |
|  | CB1 | *z* | 0.801 | 0.716 | 0.816 | 0.953 |
|  |  | *p* | 0.159 | 0.372 | 0.251 | 0.293 |
|  | D1 | *z* | 0.292 | 0.654 | 0.158 | 0.686 |
|  |  | *p* | 0.889 | 0.372 | 0.940 | 0.356 |
|  | D2 | *z* | 0.191 | 0.911 | 0.067 | 0.760 |
|  |  | *p* | 0.973 | 0.257 | 0.984 | 0.356 |
|  | DAT | *z* | 0.458 | 0.929 | 0.294 | 0.624 |
|  |  | *p* | 0.707 | 0.257 | 0.842 | 0.403 |
|  | FDOPA | *z* | 0.514 | 0.672 | 0.376 | 0.774 |
|  |  | *p* | 0.707 | 0.372 | 0.835 | 0.356 |
|  | GABAa | *z* | 0.164 | 0.133 | 0.293 | 0.083 |
|  |  | *p* | 0.973 | 0.881 | 0.842 | 0.953 |
|  | KappaOp | *z* | 0.182 | 0.105 | 0.174 | 0.162 |
|  |  | *p* | 0.973 | 0.881 | 0.940 | 0.953 |
|  | mGluR5 | *z* | 0.123 | 0.145 | 0.237 | 0.055 |
|  |  | *p* | 0.973 | 0.881 | 0.842 | 0.953 |
|  | MU | *z* | 0.047 | 0.006 | 0.017 | 0.070 |
|  |  | *p* | 0.973 | 0.987 | 0.984 | 0.953 |
|  | NA | *z* | 0.201 | 0.032 | 0.332 | 0.305 |
|  |  | *p* | 0.973 | 0.986 | 0.842 | 0.883 |
|  | NMDA | *z* | 0.032 | 0.197 | 0.027 | 0.302 |
|  |  | *p* | 0.973 | 0.881 | 0.984 | 0.883 |
|  | SERT_DAS | *z* | 0.307 | 0.227 | 0.366 | 0.247 |
|  |  | *p* | 0.889 | 0.881 | 0.835 | 0.889 |
|  | SERT_MADAM | *z* | 0.423 | 0.213 | 0.405 | 0.290 |
|  |  | *p* | 0.707 | 0.881 | 0.835 | 0.883 |
|  | VACh | *z* | 0.432 | 0.818 | 0.265 | 0.681 |
|  |  | *p* | 0.707 | 0.283 | 0.842 | 0.356 |

The trend-surface regression model with nine coefficients was applied to group-level gradient maps and the neurotransmitter PET/SPECT scans. Fisher’s r-to-z–transformed correlation coefficients were computed between the TSM coefficients representing gradient maps and those representing neurotransmitter maps. The p-value was estimated using a permutation test as the proportion of permuted z-values that were lower than the observed Fisher r-to-z–transformed correlation coefficients, with FDR correction applied. The correction was applied across 18 neurotransmitters within each gradient, and separately for each hemisphere and subgroup. **p*<0.05.

**Table S13**. Correlations coefficients (Fisher r-to-z transformed, absolute values, with FDR correction) between gradient and neurotransmitter layouts, separately for patients and healthy controls in MIND-Set (data is from resting-state 1)

|  |  |  | **Patient_Left** | **Patient_Right** | **Healthy Control_Left** | **Healthy Control_Right** |
| --- | --- | --- | --- | --- | --- | --- |
| **Zeroth-order gradient** | 5HT1A | *z* | 0.893 | 0.802 | 0.974 | 0.774 |
|  |  | *p* | 0.903 | 0.897 | 0.941 | 0.890 |
|  | 5HT1B | *z* | 0.373 | 0.224 | 0.340 | 0.236 |
|  |  | *p* | 0.903 | 0.897 | 0.941 | 0.890 |
|  | 5HT2A | *z* | 0.017 | 0.094 | 0.037 | 0.096 |
|  |  | *p* | 0.980 | 0.897 | 0.959 | 0.890 |
|  | 5HT4 | *z* | 0.794 | 0.482 | 0.843 | 0.468 |
|  |  | *p* | 0.903 | 0.897 | 0.941 | 0.890 |
|  | CB1 | *z* | 0.695 | 0.418 | 0.711 | 0.411 |
|  |  | *p* | 0.903 | 0.897 | 0.941 | 0.890 |
|  | D1 | *z* | 0.247 | 0.088 | 0.231 | 0.098 |
|  |  | *p* | 0.903 | 0.897 | 0.951 | 0.890 |
|  | D2 | *z* | 0.689 | 0.364 | 0.759 | 0.347 |
|  |  | *p* | 0.903 | 0.897 | 0.941 | 0.890 |
|  | DAT | *z* | 0.186 | 0.126 | 0.134 | 0.142 |
|  |  | *p* | 0.903 | 0.897 | 0.951 | 0.890 |
|  | FDOPA | *z* | 0.509 | 0.729 | 0.481 | 0.740 |
|  |  | *p* | 0.903 | 0.897 | 0.941 | 0.890 |
|  | GABAa | *z* | 0.469 | 0.198 | 0.524 | 0.181 |
|  |  | *p* | 0.903 | 0.897 | 0.941 | 0.890 |
|  | KappaOp | *z* | 0.814 | 1.181 | 0.772 | 1.198 |
|  |  | *p* | 0.903 | 0.897 | 0.941 | 0.890 |
|  | mGluR5 | *z* | 0.526 | 0.352 | 0.581 | 0.333 |
|  |  | *p* | 0.903 | 0.897 | 0.941 | 0.890 |
|  | MU | *z* | 0.458 | 0.598 | 0.423 | 0.611 |
|  |  | *p* | 0.903 | 0.897 | 0.941 | 0.890 |
|  | NA | *z* | 0.045 | 0.271 | 0.027 | 0.272 |
|  |  | *p* | 0.980 | 0.897 | 0.959 | 0.890 |
|  | NMDA | *z* | 0.179 | 0.596 | 0.158 | 0.604 |
|  |  | *p* | 0.903 | 0.897 | 0.951 | 0.890 |
|  | SERT_DAS | *z* | 0.747 | 0.862 | 0.713 | 0.872 |
|  |  | *p* | 0.903 | 0.897 | 0.941 | 0.890 |
|  | SERT_MADAM | *z* | 0.593 | 1.051 | 0.548 | 1.060 |
|  |  | *p* | 0.903 | 0.897 | 0.941 | 0.890 |
|  | VACh | *z* | 0.253 | 0.361 | 0.306 | 0.342 |
|  |  | *p* | 0.903 | 0.897 | 0.941 | 0.890 |
| **First-order gradient** | 5HT1A | *z* | 0.074 | 0.713 | 0.249 | 0.694 |
|  |  | *p* | 0.971 | 0.842 | 0.970 | 0.809 |
|  | 5HT1B | *z* | 0.729 | 0.691 | 0.601 | 0.747 |
|  |  | *p* | 0.971 | 0.842 | 0.970 | 0.809 |
|  | 5HT2A | *z* | 0.384 | 0.265 | 0.288 | 0.235 |
|  |  | *p* | 0.971 | 0.865 | 0.970 | 0.854 |
|  | 5HT4 | *z* | 0.049 | 0.664 | 0.296 | 0.626 |
|  |  | *p* | 0.971 | 0.842 | 0.970 | 0.809 |
|  | CB1 | *z* | 0.086 | 0.294 | 0.240 | 0.246 |
|  |  | *p* | 0.971 | 0.865 | 0.970 | 0.854 |
|  | D1 | *z* | 0.248 | 0.229 | 0.199 | 0.253 |
|  |  | *p* | 0.971 | 0.865 | 0.970 | 0.854 |
|  | D2 | *z* | 0.079 | 0.606 | 0.248 | 0.637 |
|  |  | *p* | 0.971 | 0.842 | 0.970 | 0.809 |
|  | DAT | *z* | 0.332 | 0.561 | 0.121 | 0.570 |
|  |  | *p* | 0.971 | 0.842 | 0.970 | 0.809 |
|  | FDOPA | *z* | 0.299 | 0.251 | 0.268 | 0.285 |
|  |  | *p* | 0.971 | 0.865 | 0.970 | 0.854 |
|  | GABAa | *z* | 0.149 | 0.778 | 0.137 | 0.735 |
|  |  | *p* | 0.971 | 0.842 | 0.970 | 0.809 |
|  | KappaOp | *z* | 1.210 | 0.469 | 1.242 | 0.484 |
|  |  | *p* | 0.241 | 0.843 | 0.348 | 0.814 |
|  | mGluR5 | *z* | 0.080 | 0.711 | 0.202 | 0.673 |
|  |  | *p* | 0.971 | 0.842 | 0.970 | 0.809 |
|  | MU | *z* | 1.327 | 0.896 | 1.099 | 0.914 |
|  |  | *p* | 0.207 | 0.842 | 0.348 | 0.809 |
|  | NA | *z* | 0.031 | 0.108 | 0.043 | 0.140 |
|  |  | *p* | 0.971 | 0.923 | 0.970 | 0.899 |
|  | NMDA | *z* | 0.067 | 0.369 | 0.025 | 0.341 |
|  |  | *p* | 0.971 | 0.865 | 0.970 | 0.854 |
|  | SERT_DAS | *z* | 0.791 | 0.575 | 0.843 | 0.594 |
|  |  | *p* | 0.971 | 0.842 | 0.745 | 0.809 |
|  | SERT_MADAM | *z* | 1.351 | 0.518 | 1.130 | 0.545 |
|  |  | *p* | 0.207 | 0.842 | 0.348 | 0.809 |
|  | VACh | *z* | 0.410 | 0.782 | 0.099 | 0.809 |
|  |  | *p* | 0.971 | 0.842 | 0.970 | 0.809 |
| **Second-order gradient** | 5HT1A | *z* | 0.195 | 0.712 | 0.283 | 1.654 |
|  |  | *p* | 0.749 | 0.682 | 0.710 | 0.189 |
|  | 5HT1B | *z* | 0.362 | 0.349 | 0.376 | 0.063 |
|  |  | *p* | 0.742 | 0.829 | 0.710 | 0.999 |
|  | 5HT2A | *z* | 0.859 | 0.745 | 0.793 | 0.240 |
|  |  | *p* | 0.443 | 0.682 | 0.531 | 0.992 |
|  | 5HT4 | *z* | 0.470 | 1.166 | 0.381 | 1.058 |
|  |  | *p* | 0.742 | 0.682 | 0.710 | 0.606 |
|  | CB1 | *z* | 0.386 | 0.872 | 0.326 | 0.605 |
|  |  | *p* | 0.742 | 0.682 | 0.710 | 0.811 |
|  | D1 | *z* | 1.502 | 0.736 | 1.467 | 0.070 |
|  |  | *p* | 0.067 | 0.682 | 0.070 | 0.999 |
|  | D2 | *z* | 0.287 | 0.163 | 0.376 | 0.753 |
|  |  | *p* | 0.742 | 0.869 | 0.710 | 0.811 |
|  | DAT | *z* | 0.202 | 0.331 | 0.155 | 0.545 |
|  |  | *p* | 0.749 | 0.829 | 0.798 | 0.811 |
|  | FDOPA | *z* | 0.839 | 0.826 | 0.821 | 0.426 |
|  |  | *p* | 0.443 | 0.682 | 0.531 | 0.908 |
|  | GABAa | *z* | 0.297 | 0.803 | 0.213 | 0.987 |
|  |  | *p* | 0.742 | 0.682 | 0.748 | 0.606 |
|  | KappaOp | *z* | 0.275 | 0.849 | 0.267 | 0.715 |
|  |  | *p* | 0.742 | 0.682 | 0.710 | 0.811 |
|  | mGluR5 | *z* | 0.369 | 0.934 | 0.282 | 1.253 |
|  |  | *p* | 0.742 | 0.682 | 0.710 | 0.535 |
|  | MU | *z* | 0.417 | 0.563 | 0.434 | 0.334 |
|  |  | *p* | 0.742 | 0.829 | 0.710 | 0.992 |
|  | NA | *z* | 0.493 | 0.243 | 0.470 | 0.002 |
|  |  | *p* | 0.742 | 0.869 | 0.710 | 0.999 |
|  | NMDA | *z* | 1.286 | 0.387 | 1.211 | 0.011 |
|  |  | *p* | 0.100 | 0.829 | 0.157 | 0.999 |
|  | SERT_DAS | *z* | 0.560 | 0.139 | 0.563 | 0.281 |
|  |  | *p* | 0.742 | 0.869 | 0.710 | 0.992 |
|  | SERT_MADAM | *z* | 0.001 | 0.522 | 0.015 | 0.563 |
|  |  | *p* | 0.999 | 0.829 | 0.978 | 0.811 |
|  | VACh | *z* | 0.259 | 0.333 | 0.334 | 0.958 |
|  |  | *p* | 0.742 | 0.829 | 0.710 | 0.606 |
| **Third-order gradient** | 5HT1A | *z* | 1.959 * | 1.925 ** | 1.874 * | 1.264 |
|  |  | *p* | 0.020 | 0.002 | 0.020 | 0.140 |
|  | 5HT1B | *z* | 0.040 | 0.181 | 0.005 | 0.587 |
|  |  | *p* | 0.974 | 0.923 | 0.994 | 0.779 |
|  | 5HT2A | *z* | 0.213 | 0.083 | 0.231 | 0.137 |
|  |  | *p* | 0.974 | 0.923 | 0.994 | 0.876 |
|  | 5HT4 | *z* | 1.104 | 0.911 | 1.163 | 0.537 |
|  |  | *p* | 0.361 | 0.567 | 0.395 | 0.779 |
|  | CB1 | *z* | 0.638 | 0.485 | 0.690 | 0.140 |
|  |  | *p* | 0.886 | 0.888 | 0.801 | 0.876 |
|  | D1 | *z* | 0.026 | 0.092 | 0.078 | 0.404 |
|  |  | *p* | 0.974 | 0.923 | 0.994 | 0.876 |
|  | D2 | *z* | 1.721 * | 0.913 | 1.560 | 1.457 |
|  |  | *p* | 0.034 | 0.567 | 0.108 | 0.113 |
|  | DAT | *z* | 0.427 | 0.501 | 0.363 | 0.562 |
|  |  | *p* | 0.974 | 0.888 | 0.994 | 0.779 |
|  | FDOPA | *z* | 0.115 | 0.295 | 0.174 | 0.077 |
|  |  | *p* | 0.974 | 0.923 | 0.994 | 0.876 |
|  | GABAa | *z* | 1.117 | 0.846 | 1.077 | 0.600 |
|  |  | *p* | 0.361 | 0.593 | 0.395 | 0.779 |
|  | KappaOp | *z* | 0.301 | 0.590 | 0.330 | 0.203 |
|  |  | *p* | 0.974 | 0.888 | 0.994 | 0.876 |
|  | mGluR5 | *z* | 1.143 | 1.060 | 1.110 | 0.698 |
|  |  | *p* | 0.361 | 0.502 | 0.395 | 0.779 |
|  | MU | *z* | 0.039 | 0.203 | 0.060 | 0.123 |
|  |  | *p* | 0.974 | 0.923 | 0.994 | 0.876 |
|  | NA | *z* | 0.129 | 0.152 | 0.098 | 0.347 |
|  |  | *p* | 0.974 | 0.923 | 0.994 | 0.876 |
|  | NMDA | *z* | 0.071 | 0.152 | 0.063 | 0.315 |
|  |  | *p* | 0.974 | 0.923 | 0.994 | 0.876 |
|  | SERT_DAS | *z* | 0.283 | 0.290 | 0.292 | 0.079 |
|  |  | *p* | 0.974 | 0.923 | 0.994 | 0.876 |
|  | SERT_MADAM | *z* | 0.075 | 0.493 | 0.100 | 0.142 |
|  |  | *p* | 0.974 | 0.888 | 0.994 | 0.876 |
|  | VACh | *z* | 0.916 | 1.111 | 0.840 | 1.494 |
|  |  | *p* | 0.540 | 0.502 | 0.683 | 0.113 |
| **Fourth-order gradient** | 5HT1A | *z* | 0.051 | 0.125 | 0.107 | 0.257 |
|  |  | *p* | 0.956 | 0.918 | 0.926 | 0.891 |
|  | 5HT1B | *z* | 0.446 | 0.403 | 0.441 | 0.346 |
|  |  | *p* | 0.846 | 0.808 | 0.787 | 0.837 |
|  | 5HT2A | *z* | 0.294 | 0.539 | 0.363 | 0.630 |
|  |  | *p* | 0.846 | 0.718 | 0.816 | 0.537 |
|  | 5HT4 | *z* | 0.110 | 0.060 | 0.041 | 0.034 |
|  |  | *p* | 0.956 | 0.918 | 0.952 | 0.947 |
|  | CB1 | *z* | 0.237 | 0.052 | 0.127 | 0.070 |
|  |  | *p* | 0.846 | 0.918 | 0.926 | 0.947 |
|  | D1 | *z* | 0.294 | 0.075 | 0.260 | 0.220 |
|  |  | *p* | 0.846 | 0.918 | 0.889 | 0.891 |
|  | D2 | *z* | 0.036 | 0.205 | 0.187 | 0.064 |
|  |  | *p* | 0.956 | 0.884 | 0.926 | 0.947 |
|  | DAT | *z* | 0.954 | 0.849 | 1.165 | 0.641 |
|  |  | *p* | 0.659 | 0.359 | 0.117 | 0.537 |
|  | FDOPA | *z* | 0.470 | 0.326 | 0.418 | 0.317 |
|  |  | *p* | 0.846 | 0.828 | 0.787 | 0.837 |
|  | GABAa | *z* | 0.296 | 0.494 | 0.468 | 0.349 |
|  |  | *p* | 0.846 | 0.718 | 0.787 | 0.837 |
|  | KappaOp | *z* | 0.506 | 0.469 | 0.416 | 0.567 |
|  |  | *p* | 0.846 | 0.718 | 0.787 | 0.595 |
|  | mGluR5 | *z* | 0.246 | 0.347 | 0.417 | 0.203 |
|  |  | *p* | 0.846 | 0.828 | 0.787 | 0.891 |
|  | MU | *z* | 0.373 | 0.259 | 0.334 | 0.342 |
|  |  | *p* | 0.846 | 0.884 | 0.816 | 0.837 |
|  | NA | *z* | 0.678 | 0.770 | 0.755 | 0.637 |
|  |  | *p* | 0.846 | 0.359 | 0.787 | 0.537 |
|  | NMDA | *z* | 0.421 | 0.782 | 0.459 | 0.897 |
|  |  | *p* | 0.846 | 0.359 | 0.787 | 0.293 |
|  | SERT_DAS | *z* | 0.541 | 0.813 | 0.487 | 1.071 |
|  |  | *p* | 0.846 | 0.359 | 0.787 | 0.139 |
|  | SERT_MADAM | *z* | 0.596 | 0.595 | 0.562 | 0.792 |
|  |  | *p* | 0.846 | 0.695 | 0.787 | 0.451 |
|  | VACh | *z* | 0.388 | 0.213 | 0.532 | 0.075 |
|  |  | *p* | 0.846 | 0.884 | 0.787 | 0.947 |
| **Fifth-order gradient** | 5HT1A | *z* | 0.537 | 0.505 | 0.288 | 0.318 |
|  |  | *p* | 0.854 | 0.948 | 0.897 | 0.693 |
|  | 5HT1B | *z* | 0.127 | 0.138 | 0.818 | 0.105 |
|  |  | *p* | 0.869 | 0.948 | 0.374 | 0.870 |
|  | 5HT2A | *z* | 0.354 | 0.361 | 0.242 | 0.494 |
|  |  | *p* | 0.854 | 0.948 | 0.897 | 0.684 |
|  | 5HT4 | *z* | 0.403 | 0.646 | 0.342 | 0.647 |
|  |  | *p* | 0.854 | 0.948 | 0.897 | 0.684 |
|  | CB1 | *z* | 0.712 | 0.633 | 0.765 | 0.306 |
|  |  | *p* | 0.854 | 0.948 | 0.374 | 0.693 |
|  | D1 | *z* | 0.051 | 0.073 | 0.086 | 0.567 |
|  |  | *p* | 0.935 | 0.989 | 0.943 | 0.684 |
|  | D2 | *z* | 0.217 | 0.163 | 0.025 | 0.133 |
|  |  | *p* | 0.854 | 0.948 | 0.967 | 0.850 |
|  | DAT | *z* | 0.318 | 0.004 | 0.347 | 0.049 |
|  |  | *p* | 0.854 | 0.995 | 0.897 | 0.920 |
|  | FDOPA | *z* | 0.197 | 0.043 | 0.346 | 0.154 |
|  |  | *p* | 0.854 | 0.989 | 0.897 | 0.850 |
|  | GABAa | *z* | 0.301 | 0.448 | 0.257 | 0.206 |
|  |  | *p* | 0.854 | 0.948 | 0.897 | 0.774 |
|  | KappaOp | *z* | 0.111 | 0.342 | 0.368 | 0.033 |
|  |  | *p* | 0.869 | 0.948 | 0.897 | 0.920 |
|  | mGluR5 | *z* | 0.225 | 0.388 | 0.210 | 0.212 |
|  |  | *p* | 0.854 | 0.948 | 0.897 | 0.774 |
|  | MU | *z* | 0.402 | 0.843 | 0.187 | 0.328 |
|  |  | *p* | 0.854 | 0.948 | 0.898 | 0.693 |
|  | NA | *z* | 0.099 | 0.181 | 0.306 | 0.470 |
|  |  | *p* | 0.869 | 0.948 | 0.897 | 0.684 |
|  | NMDA | *z* | 0.364 | 0.192 | 0.119 | 0.304 |
|  |  | *p* | 0.854 | 0.948 | 0.943 | 0.693 |
|  | SERT_DAS | *z* | 0.257 | 0.307 | 0.650 | 0.541 |
|  |  | *p* | 0.854 | 0.948 | 0.381 | 0.684 |
|  | SERT_MADAM | *z* | 0.027 | 0.330 | 0.658 | 0.363 |
|  |  | *p* | 0.937 | 0.948 | 0.381 | 0.693 |
|  | VACh | *z* | 0.113 | 0.230 | 0.262 | 0.235 |
|  |  | *p* | 0.869 | 0.948 | 0.897 | 0.774 |

The trend-surface regression model with nine coefficients was applied to group-level gradient maps and the neurotransmitter PET/SPECT scans. Fisher’s r-to-z–transformed correlation coefficients were computed between the TSM coefficients representing gradient maps and those representing neurotransmitter maps. The p-value was estimated using a permutation test as the proportion of permuted z-values that were lower than the observed Fisher r-to-z–transformed correlation coefficients, with FDR correction applied. The correction was applied across 18 neurotransmitters within each gradient, and separately for each hemisphere and subgroup. *p<0.05, **p<0.01.

**Table S14**. Correlations coefficients (Fisher r-to-z transformed, absolute values, with FDR correction) between gradient and neurotransmitter layouts, separately for medicated and non-medicated participants in the MIND-Set sample (data is from resting-state 1)

|  |  |  | **Medicated _Left** | **Medicated _Right** | **Non-medicated_Left** | **Non-medicated _Right** |
| --- | --- | --- | --- | --- | --- | --- |
| **Zeroth-order gradient** | 5HT1A | *z* | 0.918 | 0.796 | 0.950 | 0.770 |
|  |  | *p* | 0.911 | 0.908 | 0.927 | 0.890 |
|  | 5HT1B | *z* | 0.368 | 0.229 | 0.352 | 0.234 |
|  |  | *p* | 0.911 | 0.908 | 0.927 | 0.890 |
|  | 5HT2A | *z* | 0.024 | 0.100 | 0.034 | 0.100 |
|  |  | *p* | 0.971 | 0.908 | 0.959 | 0.890 |
|  | 5HT4 | *z* | 0.811 | 0.474 | 0.833 | 0.463 |
|  |  | *p* | 0.911 | 0.908 | 0.927 | 0.890 |
|  | CB1 | *z* | 0.704 | 0.417 | 0.711 | 0.405 |
|  |  | *p* | 0.911 | 0.908 | 0.927 | 0.890 |
|  | D1 | *z* | 0.242 | 0.082 | 0.239 | 0.095 |
|  |  | *p* | 0.911 | 0.908 | 0.927 | 0.890 |
|  | D2 | *z* | 0.709 | 0.359 | 0.737 | 0.347 |
|  |  | *p* | 0.911 | 0.908 | 0.927 | 0.890 |
|  | DAT | *z* | 0.170 | 0.130 | 0.149 | 0.143 |
|  |  | *p* | 0.911 | 0.908 | 0.927 | 0.890 |
|  | FDOPA | *z* | 0.501 | 0.729 | 0.493 | 0.735 |
|  |  | *p* | 0.911 | 0.908 | 0.927 | 0.890 |
|  | GABAa | *z* | 0.488 | 0.195 | 0.510 | 0.176 |
|  |  | *p* | 0.911 | 0.908 | 0.927 | 0.890 |
|  | KappaOp | *z* | 0.809 | 1.188 | 0.787 | 1.195 |
|  |  | *p* | 0.911 | 0.908 | 0.927 | 0.890 |
|  | mGluR5 | *z* | 0.544 | 0.348 | 0.567 | 0.328 |
|  |  | *p* | 0.911 | 0.908 | 0.927 | 0.890 |
|  | MU | *z* | 0.452 | 0.604 | 0.435 | 0.611 |
|  |  | *p* | 0.911 | 0.908 | 0.927 | 0.890 |
|  | NA | *z* | 0.036 | 0.267 | 0.031 | 0.276 |
|  |  | *p* | 0.971 | 0.908 | 0.959 | 0.890 |
|  | NMDA | *z* | 0.173 | 0.602 | 0.162 | 0.607 |
|  |  | *p* | 0.911 | 0.908 | 0.927 | 0.890 |
|  | SERT_DAS | *z* | 0.744 | 0.879 | 0.721 | 0.872 |
|  |  | *p* | 0.911 | 0.908 | 0.927 | 0.890 |
|  | SERT_MADAM | *z* | 0.587 | 1.069 | 0.562 | 1.057 |
|  |  | *p* | 0.911 | 0.908 | 0.927 | 0.890 |
|  | VACh | *z* | 0.267 | 0.355 | 0.289 | 0.341 |
|  |  | *p* | 0.911 | 0.908 | 0.927 | 0.890 |
| **First-order gradient** | 5HT1A | *z* | 0.028 | 0.706 | 0.071 | 0.721 |
|  |  | *p* | 0.999 | 0.809 | 0.989 | 0.838 |
|  | 5HT1B | *z* | 0.697 | 0.705 | 0.667 | 0.722 |
|  |  | *p* | 0.999 | 0.809 | 0.989 | 0.838 |
|  | 5HT2A | *z* | 0.365 | 0.270 | 0.341 | 0.237 |
|  |  | *p* | 0.999 | 0.868 | 0.989 | 0.838 |
|  | 5HT4 | *z* | 0.125 | 0.664 | 0.161 | 0.647 |
|  |  | *p* | 0.999 | 0.809 | 0.989 | 0.838 |
|  | CB1 | *z* | 0.136 | 0.282 | 0.149 | 0.268 |
|  |  | *p* | 0.999 | 0.868 | 0.989 | 0.838 |
|  | D1 | *z* | 0.237 | 0.219 | 0.226 | 0.250 |
|  |  | *p* | 0.999 | 0.868 | 0.989 | 0.838 |
|  | D2 | *z* | 0.023 | 0.615 | 0.073 | 0.635 |
|  |  | *p* | 0.999 | 0.809 | 0.989 | 0.838 |
|  | DAT | *z* | 0.282 | 0.569 | 0.212 | 0.561 |
|  |  | *p* | 0.999 | 0.809 | 0.989 | 0.838 |
|  | FDOPA | *z* | 0.298 | 0.257 | 0.278 | 0.266 |
|  |  | *p* | 0.999 | 0.868 | 0.989 | 0.838 |
|  | GABAa | *z* | 0.067 | 0.769 | 0.010 | 0.753 |
|  |  | *p* | 0.999 | 0.809 | 0.989 | 0.838 |
|  | KappaOp | *z* | 1.261 | 0.470 | 1.250 | 0.464 |
|  |  | *p* | 0.190 | 0.842 | 0.164 | 0.838 |
|  | mGluR5 | *z* | 0.001 | 0.702 | 0.057 | 0.693 |
|  |  | *p* | 0.999 | 0.809 | 0.989 | 0.838 |
|  | MU | *z* | 1.279 | 0.898 | 1.254 | 0.895 |
|  |  | *p* | 0.190 | 0.809 | 0.164 | 0.838 |
|  | NA | *z* | 0.024 | 0.121 | 0.018 | 0.138 |
|  |  | *p* | 0.999 | 0.915 | 0.989 | 0.894 |
|  | NMDA | *z* | 0.066 | 0.371 | 0.031 | 0.336 |
|  |  | *p* | 0.999 | 0.868 | 0.989 | 0.838 |
|  | SERT_DAS | *z* | 0.832 | 0.597 | 0.809 | 0.567 |
|  |  | *p* | 0.866 | 0.809 | 0.829 | 0.838 |
|  | SERT_MADAM | *z* | 1.321 | 0.534 | 1.248 | 0.517 |
|  |  | *p* | 0.190 | 0.809 | 0.164 | 0.838 |
|  | VACh | *z* | 0.314 | 0.792 | 0.255 | 0.811 |
|  |  | *p* | 0.999 | 0.809 | 0.989 | 0.838 |
| **Second-order gradient** | 5HT1A | *z* | 0.250 | 0.506 | 0.280 | 1.358 |
|  |  | *p* | 0.669 | 0.795 | 0.687 | 0.479 |
|  | 5HT1B | *z* | 0.303 | 0.244 | 0.340 | 0.105 |
|  |  | *p* | 0.669 | 0.848 | 0.687 | 0.927 |
|  | 5HT2A | *z* | 0.854 | 1.031 | 0.823 | 0.396 |
|  |  | *p* | 0.424 | 0.404 | 0.496 | 0.858 |
|  | 5HT4 | *z* | 0.395 | 1.074 | 0.372 | 1.247 |
|  |  | *p* | 0.669 | 0.404 | 0.687 | 0.479 |
|  | CB1 | *z* | 0.313 | 0.715 | 0.308 | 0.787 |
|  |  | *p* | 0.669 | 0.637 | 0.687 | 0.858 |
|  | D1 | *z* | 1.305 | 0.970 | 1.330 | 0.267 |
|  |  | *p* | 0.062 | 0.404 | 0.075 | 0.891 |
|  | D2 | *z* | 0.328 | 0.129 | 0.366 | 0.517 |
|  |  | *p* | 0.669 | 0.859 | 0.687 | 0.858 |
|  | DAT | *z* | 0.245 | 0.417 | 0.214 | 0.451 |
|  |  | *p* | 0.669 | 0.824 | 0.687 | 0.858 |
|  | FDOPA | *z* | 0.734 | 0.661 | 0.755 | 0.599 |
|  |  | *p* | 0.541 | 0.675 | 0.496 | 0.858 |
|  | GABAa | *z* | 0.267 | 0.758 | 0.240 | 0.998 |
|  |  | *p* | 0.669 | 0.637 | 0.687 | 0.782 |
|  | KappaOp | *z* | 0.199 | 0.585 | 0.237 | 0.813 |
|  |  | *p* | 0.695 | 0.675 | 0.687 | 0.858 |
|  | mGluR5 | *z* | 0.331 | 0.817 | 0.305 | 1.258 |
|  |  | *p* | 0.669 | 0.631 | 0.687 | 0.479 |
|  | MU | *z* | 0.358 | 0.415 | 0.407 | 0.414 |
|  |  | *p* | 0.669 | 0.824 | 0.687 | 0.858 |
|  | NA | *z* | 0.520 | 0.279 | 0.532 | 0.077 |
|  |  | *p* | 0.669 | 0.848 | 0.687 | 0.927 |
|  | NMDA | *z* | 1.341 | 0.611 | 1.292 | 0.115 |
|  |  | *p* | 0.062 | 0.675 | 0.075 | 0.927 |
|  | SERT_DAS | *z* | 0.646 | 0.142 | 0.597 | 0.277 |
|  |  | *p* | 0.635 | 0.859 | 0.687 | 0.891 |
|  | SERT_MADAM | *z* | 0.064 | 0.230 | 0.018 | 0.611 |
|  |  | *p* | 0.881 | 0.848 | 0.969 | 0.858 |
|  | VACh | *z* | 0.256 | 0.295 | 0.297 | 0.710 |
|  |  | *p* | 0.669 | 0.848 | 0.687 | 0.858 |
| **Third-order gradient** | 5HT1A | *z* | 1.947 * | 1.931 *** | 1.936 * | 1.863 ** |
|  |  | *p* | 0.014 | 0.000 | 0.011 | 0.004 |
|  | 5HT1B | *z* | 0.037 | 0.187 | 0.035 | 0.276 |
|  |  | *p* | 0.966 | 0.924 | 0.966 | 0.908 |
|  | 5HT2A | *z* | 0.234 | 0.103 | 0.227 | 0.032 |
|  |  | *p* | 0.966 | 0.924 | 0.966 | 0.966 |
|  | 5HT4 | *z* | 1.140 | 0.926 | 1.129 | 0.824 |
|  |  | *p* | 0.330 | 0.549 | 0.330 | 0.582 |
|  | CB1 | *z* | 0.657 | 0.492 | 0.651 | 0.404 |
|  |  | *p* | 0.853 | 0.887 | 0.850 | 0.908 |
|  | D1 | *z* | 0.048 | 0.085 | 0.043 | 0.171 |
|  |  | *p* | 0.966 | 0.924 | 0.966 | 0.908 |
|  | D2 | *z* | 1.680 | 0.926 | 1.687 * | 1.061 |
|  |  | *p* | 0.070 | 0.549 | 0.041 | 0.420 |
|  | DAT | *z* | 0.424 | 0.532 | 0.427 | 0.540 |
|  |  | *p* | 0.966 | 0.887 | 0.966 | 0.908 |
|  | FDOPA | *z* | 0.134 | 0.294 | 0.130 | 0.208 |
|  |  | *p* | 0.966 | 0.924 | 0.966 | 0.908 |
|  | GABAa | *z* | 1.134 | 0.886 | 1.130 | 0.817 |
|  |  | *p* | 0.330 | 0.549 | 0.330 | 0.582 |
|  | KappaOp | *z* | 0.298 | 0.586 | 0.307 | 0.504 |
|  |  | *p* | 0.966 | 0.887 | 0.966 | 0.908 |
|  | mGluR5 | *z* | 1.162 | 1.107 | 1.159 | 1.005 |
|  |  | *p* | 0.330 | 0.441 | 0.330 | 0.420 |
|  | MU | *z* | 0.036 | 0.206 | 0.045 | 0.135 |
|  |  | *p* | 0.966 | 0.924 | 0.966 | 0.908 |
|  | NA | *z* | 0.130 | 0.123 | 0.133 | 0.184 |
|  |  | *p* | 0.966 | 0.924 | 0.966 | 0.908 |
|  | NMDA | *z* | 0.085 | 0.129 | 0.082 | 0.195 |
|  |  | *p* | 0.966 | 0.924 | 0.966 | 0.908 |
|  | SERT_DAS | *z* | 0.266 | 0.275 | 0.275 | 0.249 |
|  |  | *p* | 0.966 | 0.924 | 0.966 | 0.908 |
|  | SERT_MADAM | *z* | 0.067 | 0.485 | 0.075 | 0.419 |
|  |  | *p* | 0.966 | 0.887 | 0.966 | 0.908 |
|  | VACh | *z* | 0.907 | 1.133 | 0.908 | 1.253 |
|  |  | *p* | 0.592 | 0.441 | 0.548 | 0.333 |
| **Fourth-order gradient** | 5HT1A | *z* | 0.003 | 0.242 | 0.068 | 0.192 |
|  |  | *p* | 0.995 | 0.928 | 0.942 | 0.960 |
|  | 5HT1B | *z* | 0.485 | 0.394 | 0.491 | 0.321 |
|  |  | *p* | 0.863 | 0.837 | 0.872 | 0.862 |
|  | 5HT2A | *z* | 0.235 | 0.435 | 0.236 | 0.605 |
|  |  | *p* | 0.863 | 0.837 | 0.872 | 0.576 |
|  | 5HT4 | *z* | 0.119 | 0.069 | 0.148 | 0.051 |
|  |  | *p* | 0.934 | 0.947 | 0.913 | 0.974 |
|  | CB1 | *z* | 0.257 | 0.130 | 0.300 | 0.001 |
|  |  | *p* | 0.863 | 0.933 | 0.872 | 0.999 |
|  | D1 | *z* | 0.375 | 0.004 | 0.328 | 0.142 |
|  |  | *p* | 0.863 | 0.992 | 0.872 | 0.960 |
|  | D2 | *z* | 0.096 | 0.127 | 0.044 | 0.094 |
|  |  | *p* | 0.934 | 0.933 | 0.942 | 0.960 |
|  | DAT | *z* | 0.979 | 0.708 | 0.980 | 0.677 |
|  |  | *p* | 0.619 | 0.642 | 0.504 | 0.543 |
|  | FDOPA | *z* | 0.541 | 0.400 | 0.518 | 0.289 |
|  |  | *p* | 0.863 | 0.837 | 0.872 | 0.862 |
|  | GABAa | *z* | 0.305 | 0.364 | 0.262 | 0.440 |
|  |  | *p* | 0.863 | 0.837 | 0.872 | 0.808 |
|  | KappaOp | *z* | 0.502 | 0.574 | 0.504 | 0.536 |
|  |  | *p* | 0.863 | 0.686 | 0.872 | 0.671 |
|  | mGluR5 | *z* | 0.252 | 0.218 | 0.217 | 0.284 |
|  |  | *p* | 0.863 | 0.928 | 0.872 | 0.862 |
|  | MU | *z* | 0.387 | 0.306 | 0.360 | 0.321 |
|  |  | *p* | 0.863 | 0.887 | 0.872 | 0.862 |
|  | NA | *z* | 0.647 | 0.774 | 0.636 | 0.794 |
|  |  | *p* | 0.863 | 0.642 | 0.872 | 0.457 |
|  | NMDA | *z* | 0.351 | 0.705 | 0.394 | 0.843 |
|  |  | *p* | 0.863 | 0.642 | 0.872 | 0.457 |
|  | SERT_DAS | *z* | 0.470 | 0.784 | 0.535 | 0.856 |
|  |  | *p* | 0.863 | 0.642 | 0.872 | 0.457 |
|  | SERT_MADAM | *z* | 0.581 | 0.646 | 0.603 | 0.654 |
|  |  | *p* | 0.863 | 0.651 | 0.872 | 0.543 |
|  | VACh | *z* | 0.449 | 0.113 | 0.404 | 0.099 |
|  |  | *p* | 0.863 | 0.933 | 0.872 | 0.960 |
| **Fifth-order gradient** | 5HT1A | *z* | 0.555 | 0.277 | 0.134 | 0.201 |
|  |  | *p* | 0.808 | 0.953 | 0.977 | 0.789 |
|  | 5HT1B | *z* | 0.184 | 0.114 | 0.658 | 0.465 |
|  |  | *p* | 0.840 | 0.953 | 0.641 | 0.557 |
|  | 5HT2A | *z* | 0.365 | 0.172 | 0.017 | 0.205 |
|  |  | *p* | 0.808 | 0.953 | 0.977 | 0.789 |
|  | 5HT4 | *z* | 0.446 | 0.336 | 0.145 | 0.034 |
|  |  | *p* | 0.808 | 0.953 | 0.977 | 0.968 |
|  | CB1 | *z* | 0.777 | 0.636 | 0.299 | 0.505 |
|  |  | *p* | 0.628 | 0.485 | 0.910 | 0.557 |
|  | D1 | *z* | 0.009 | 0.037 | 0.029 | 0.261 |
|  |  | *p* | 0.981 | 0.953 | 0.977 | 0.789 |
|  | D2 | *z* | 0.221 | 0.109 | 0.017 | 0.214 |
|  |  | *p* | 0.808 | 0.953 | 0.977 | 0.789 |
|  | DAT | *z* | 0.337 | 0.317 | 0.009 | 0.460 |
|  |  | *p* | 0.808 | 0.953 | 0.977 | 0.557 |
|  | FDOPA | *z* | 0.247 | 0.125 | 0.086 | 0.313 |
|  |  | *p* | 0.808 | 0.953 | 0.977 | 0.789 |
|  | GABAa | *z* | 0.319 | 0.206 | 0.209 | 0.069 |
|  |  | *p* | 0.808 | 0.953 | 0.977 | 0.962 |
|  | KappaOp | *z* | 0.050 | 0.283 | 0.410 | 0.130 |
|  |  | *p* | 0.937 | 0.953 | 0.750 | 0.918 |
|  | mGluR5 | *z* | 0.247 | 0.153 | 0.195 | 0.078 |
|  |  | *p* | 0.808 | 0.953 | 0.977 | 0.962 |
|  | MU | *z* | 0.337 | 0.630 | 0.372 | 0.013 |
|  |  | *p* | 0.808 | 0.485 | 0.750 | 0.968 |
|  | NA | *z* | 0.088 | 0.052 | 0.496 | 0.188 |
|  |  | *p* | 0.911 | 0.953 | 0.641 | 0.789 |
|  | NMDA | *z* | 0.350 | 0.018 | 0.122 | 0.326 |
|  |  | *p* | 0.808 | 0.953 | 0.977 | 0.789 |
|  | SERT_DAS | *z* | 0.296 | 0.036 | 0.527 | 0.807 |
|  |  | *p* | 0.808 | 0.953 | 0.641 | 0.358 |
|  | SERT_MADAM | *z* | 0.084 | 0.094 | 0.611 | 0.562 |
|  |  | *p* | 0.911 | 0.953 | 0.641 | 0.557 |
|  | VACh | *z* | 0.091 | 0.089 | 0.180 | 0.247 |
|  |  | *p* | 0.911 | 0.953 | 0.977 | 0.789 |

The trend-surface regression model with nine coefficients was applied to group-level gradient maps and the neurotransmitter PET/SPECT scans. Fisher’s r-to-z–transformed correlation coefficients were computed between the TSM coefficients representing gradient maps and those representing neurotransmitter maps. The p-value was estimated using a permutation test as the proportion of permuted z-values that were lower than the observed Fisher r-to-z–transformed correlation coefficients, with FDR correction applied. The correction was applied across 18 neurotransmitters within each gradient, and separately for each hemisphere and subgroup. **p*<0.05, ***p*<0.01, ****p*<0.001.

**Table S15.** Spearman correlations between gradient-neurotransmitter similarity and mental health outcomes in HBS

|  |  | **Anxiety Sensitivity** | **Depressive Severity** | **Childhood**  **Trauma** | **Emotional**  **Neglect** | **Physical**  **Neglect** | **Emotional Abuse** | **Physical**  **Abuse** |
| --- | --- | --- | --- | --- | --- | --- | --- | --- |
| **5HT1A_ Resting-** | *r_s_* | 0.160 | 0.190 * | 0.130 | 0.100 | 0.120 | 0.100 | 0.140 |
| **State 1_Left** | *p* | 0.072 | 0.021 | 0.145 | 0.245 | 0.169 | 0.234 | 0.125 |
| **5HT1A_ Resting-** | *r_s_* | -0.130 | 0.050 | 0.030 | 0.010 | 0.060 | 0.000 | 0.070 |
| **State 1_Right** | *p* | 0.145 | 0.567 | 0.774 | 0.952 | 0.538 | 0.988 | 0.431 |
| N = 179 |  |  |  |  |  |  |  |  |
| **5HT1A_ Resting-** | *r_s_* | 0.080 | -0.020 | -0.040 | -0.060 | 0.020 | 0.030 | -0.060 |
| **State 2_Left** | *p* | 0.451 | 0.855 | 0.757 | 0.612 | 0.836 | 0.836 | 0.612 |
| **5HT1A_ Resting-** | *r_s_* | -0.060 | 0.020 | -0.060 | 0.010 | -0.070 | -0.100 | 0.000 |
| **State 2_Right** | *p* | 0.601 | 0.855 | 0.612 | 0.934 | 0.601 | 0.359 | 0.958 |
| N = 187 |  |  |  |  |  |  |  |  |
| **5HT1A_ Resting-** | *r_s_* | 0.010 | 0.140 | -0.150 | -0.160 | -0.070 | -0.030 | -0.040 |
| **State 3_Left** | *p* | 0.933 | 0.112 | 0.093 | 0.078 | 0.471 | 0.761 | 0.737 |
| **5HT1A_ Resting-** | *r_s_* | -0.100 | -0.090 | -0.070 | -0.100 | 0.030 | -0.010 | -0.040 |
| **State 3_Right** | *p* | 0.333 | 0.374 | 0.471 | 0.333 | 0.756 | 0.938 | 0.737 |
| N = 173 |  |  |  |  |  |  |  |  |
| **D1_ Resting-** | *r_s_* | 0.060 | 0.040 | 0.030 | 0.030 | 0.060 | -0.040 | 0.010 |
| **State 1_Left** | *p* | 0.573 | 0.664 | 0.664 | 0.664 | 0.571 | 0.664 | 0.847 |
| N = 204 |  |  |  |  |  |  |  |  |
| **D1_ Resting-** | *r_s_* | 0.060 | -0.020 | -0.070 | -0.080 | 0.000 | -0.080 | 0.010 |
| **State 2_Left** | *p* | 0.500 | 0.875 | 0.409 | 0.378 | 0.992 | 0.378 | 0.929 |
| N = 200 |  |  |  |  |  |  |  |  |
| **D1_ Resting-** | *r_s_* | -0.040 | 0.140 | -0.020 | -0.030 | 0.090 | -0.050 | 0.010 |
| **State 3_Left** | *p* | 0.729 | 0.103 | 0.890 | 0.745 | 0.329 | 0.714 | 0.890 |
| N = 183 |  |  |  |  |  |  |  |  |
| **DAT_ Resting-** | *r_s_* | 0.030 | 0.010 | 0.020 | -0.020 | 0.080 | 0.020 | 0.080 |
| **State 1_Left** | *p* | 0.817 | 0.884 | 0.817 | 0.864 | 0.392 | 0.817 | 0.392 |
| N = 180 |  |  |  |  |  |  |  |  |
| **DAT_ Resting-** | *r_s_* | 0.010 | -0.030 | -0.080 | -0.070 | 0.000 | -0.130 | -0.020 |
| **State 2_Left** | *p* | 0.929 | 0.805 | 0.388 | 0.435 | 0.969 | 0.135 | 0.853 |
| N = 181 |  |  |  |  |  |  |  |  |
| **DAT_ Resting-** | *r_s_* | -0.180 * | 0.040 | 0.030 | 0.030 | 0.080 | 0.030 | -0.060 |
| **State 3_Left** | *p* | 0.048 | 0.707 | 0.778 | 0.778 | 0.494 | 0.778 | 0.613 |
| N = 156 |  |  |  |  |  |  |  |  |

To simplify, only names of neurotransmitters were listed to indicate the gradient-neurotransmitter similarity. The similarity between 5HT1A and the third-order gradient maps, D1 and the second-order gradient maps, DAT and the fourth-order gradient maps were included to correlate with mental health outcomes. The correction was applied across behavioral measures within each neurotransmitter and separately for each resting-state. Depressive severity was measured as the sum score of IDS-SR, and anxiety sensitivity was assessed with the ASI sum score. Childhood trauma and its subcategories were measured using the CTQ-SF. **p*<0.05.

**Table S16.** Spearman correlations between gradient-neurotransmitter similarity and mental health outcomes in MIND-Set

|  |  | **Anxiety Sensitivity** | **Depressive Severity** | **Comorbidity** | **Childhood**  **Trauma** | **Emotional**  **Neglect** | **Psychological Abuse** | **Physical**  **Abuse** |
| --- | --- | --- | --- | --- | --- | --- | --- | --- |
| **5HT1A_ Resting-** | *r_s_* | -0.050 | 0.030 | 0.050 | -0.050 | -0.050 | 0.010 | -0.090 |
| **State 1_Left** | *p* | 0.498 | 0.674 | 0.510 | 0.513 | 0.500 | 0.913 | 0.218 |
| **5HT1A_ Resting-** | *r_s_* | 0.150 * | 0.130 | 0.070 | -0.080 | -0.070 | -0.050 | -0.060 |
| **State 1_Right** | *p* | 0.038 | 0.057 | 0.400 | 0.288 | 0.360 | 0.510 | 0.401 |
| N = 237 |  |  |  |  |  |  |  |  |
| **5HT1A_ Resting-** | *r_s_* | 0.080 | -0.010 | 0.040 | 0.080 | 0.080 | 0.080 | 0.090 |
| **State 2_Left** | *p* | 0.362 | 0.919 | 0.651 | 0.362 | 0.362 | 0.362 | 0.324 |
| **5HT1A_ Resting-** | *r_s_* | -0.070 | 0.030 | -0.020 | 0.030 | -0.010 | 0.030 | 0.040 |
| **State 2_Right** | *p* | 0.452 | 0.749 | 0.803 | 0.748 | 0.925 | 0.745 | 0.651 |
| N = 207 |  |  |  |  |  |  |  |  |
| **5HT1A_ Resting-** | *r_s_* | -0.010 | 0.020 | 0.070 | 0.050 | 0.070 | 0.050 | -0.030 |
| **State 3_Left** | *p* | 0.909 | 0.875 | 0.400 | 0.652 | 0.380 | 0.652 | 0.736 |
| **5HT1A_ Resting-** | *r_s_* | 0.090 | 0.080 | -0.010 | -0.010 | -0.040 | 0.010 | 0.020 |
| **State 3_Right** | *p* | 0.255 | 0.349 | 0.896 | 0.924 | 0.736 | 0.909 | 0.875 |
| N = 236 |  |  |  |  |  |  |  |  |
| **D2_ Resting-** | *r_s_* | -0.020 | 0.030 | 0.030 | 0.010 | 0.020 | 0.050 | -0.060 |
| **State 1_Left** | *p* | 0.821 | 0.767 | 0.767 | 0.876 | 0.833 | 0.544 | 0.371 |
| N = 266 |  |  |  |  |  |  |  |  |
| **D2_ Resting-** | *r_s_* | -0.010 | -0.040 | 0.040 | 0.050 | 0.100 | 0.050 | 0.040 |
| **State 2_Left** | *p* | 0.862 | 0.513 | 0.513 | 0.495 | 0.149 | 0.513 | 0.513 |
| N = 261 |  |  |  |  |  |  |  |  |
| **D2_ Resting-** | *r_s_* | 0.030 | 0.080 | 0.070 | 0.070 | 0.080 | 0.070 | 0.060 |
| **State 3_Left** | *p* | 0.594 | 0.238 | 0.252 | 0.258 | 0.242 | 0.275 | 0.355 |
| N = 278 |  |  |  |  |  |  |  |  |
| **D1_ Resting-** | *r_s_* | 0.000 | 0.020 | -0.040 | -0.010 | -0.070 | 0.030 | 0.020 |
| **State 1_Left** | *p* | 0.973 | 0.849 | 0.557 | 0.882 | 0.350 | 0.785 | 0.849 |
| N = 279 |  |  |  |  |  |  |  |  |
| **D1_ Resting-** | *r_s_* | 0.130 * | 0.090 | 0.050 | 0.030 | 0.040 | 0.040 | 0.010 |
| **State 2_Left** | *p* | 0.046 | 0.172 | 0.485 | 0.701 | 0.529 | 0.592 | 0.922 |
| N = 272 |  |  |  |  |  |  |  |  |
| **D1_ Resting-** | *r_s_* | 0.090 | 0.070 | -0.060 | 0.080 | 0.080 | 0.060 | 0.020 |
| **State 3_Left** | *p* | 0.150 | 0.244 | 0.334 | 0.244 | 0.197 | 0.340 | 0.742 |
| N = 278 |  |  |  |  |  |  |  |  |

The similarity between 5HT1A and the third-order gradient maps, D2 and the third-order gradient maps, D1 and the second-order gradient maps were included to correlate with mental health outcomes. The correction was applied across behavioral measures within each neurotransmitter and separately for each resting-state. Depressive severity was measured as the sum score of IDS-SR, and anxiety sensitivity was assessed with the ASI sum score. Childhood trauma and its subcategories were assessed using the NEMESIS. Comorbidity was indexed by the number of diagnosed psychiatric disorder clusters per participant. **p*<0.05.

**Table S17.** Spearman correlations between gradient-neurotransmitter similarity and mental health outcomes in HBS (controlling for age, sex, head motion)

|  |  | **Anxiety Sensitivity** | **Depressive Severity** | **Childhood**  **Trauma** | **Emotional**  **Neglect** | **Physical**  **Neglect** | **Emotional Abuse** | **Physical**  **Abuse** |
| --- | --- | --- | --- | --- | --- | --- | --- | --- |
| **5HT1A_ Resting-** | *r_s_* | 0.160 | 0.200 * | 0.100 | 0.090 | 0.150 | 0.010 | 0.010 |
| **State 1_Left** | *p* | 0.066 | 0.022 | 0.276 | 0.299 | 0.099 | 0.930 | 0.930 |
| **5HT1A_ Resting-** | *r_s_* | -0.130 | 0.050 | 0.010 | 0.010 | 0.080 | -0.010 | 0.020 |
| **State 1_Right** | *p* | 0.166 | 0.643 | 0.930 | 0.930 | 0.364 | 0.930 | 0.918 |
| N = 179 |  |  |  |  |  |  |  |  |
| **5HT1A_ Resting-** | *r_s_* | 0.100 | -0.010 | -0.060 | -0.070 | 0.000 | -0.020 | -0.110 |
| **State 2_Left** | *p* | 0.314 | 0.992 | 0.567 | 0.553 | 0.998 | 0.856 | 0.271 |
| **5HT1A_ Resting-** | *r_s_* | -0.040 | 0.000 | -0.030 | 0.030 | -0.020 | -0.070 | 0.120 |
| **State 2_Right** | *p* | 0.806 | 0.998 | 0.839 | 0.839 | 0.856 | 0.553 | 0.194 |
| N = 187 |  |  |  |  |  |  |  |  |
| **5HT1A_ Resting-** | *r_s_* | 0.010 | 0.130 | -0.160 | -0.160 | -0.040 | -0.100 | -0.100 |
| **State 3_Left** | *p* | 0.930 | 0.153 | 0.074 | 0.067 | 0.687 | 0.286 | 0.272 |
| **5HT1A_ Resting-** | *r_s_* | -0.090 | -0.090 | -0.070 | -0.100 | 0.050 | -0.030 | -0.020 |
| **State 3_Right** | *p* | 0.335 | 0.286 | 0.425 | 0.286 | 0.597 | 0.756 | 0.855 |
| N = 173 |  |  |  |  |  |  |  |  |
| **D1_ Resting-** | *r_s_* | 0.060 | 0.040 | 0.020 | 0.030 | 0.050 | -0.030 | 0.050 |
| **State 1_Left** | *p* | 0.565 | 0.680 | 0.747 | 0.680 | 0.587 | 0.680 | 0.577 |
| N = 204 |  |  |  |  |  |  |  |  |
| **D1_ Resting-** | *r_s_* | 0.040 | -0.010 | -0.110 | -0.090 | -0.060 | -0.110 | -0.100 |
| **State 2_Left** | *p* | 0.643 | 0.907 | 0.198 | 0.257 | 0.462 | 0.190 | 0.198 |
| N = 200 |  |  |  |  |  |  |  |  |
| **D1_ Resting-** | *r_s_* | -0.040 | 0.130 | -0.030 | -0.040 | 0.070 | -0.090 | -0.050 |
| **State 3_Left** | *p* | 0.630 | 0.125 | 0.669 | 0.662 | 0.402 | 0.368 | 0.598 |
| N = 183 |  |  |  |  |  |  |  |  |
| **DAT_ Resting-** | *r_s_* | 0.030 | 0.010 | 0.010 | -0.020 | 0.110 | -0.010 | 0.010 |
| **State 1_Left** | *p* | 0.853 | 0.921 | 0.921 | 0.921 | 0.228 | 0.940 | 0.940 |
| N = 180 |  |  |  |  |  |  |  |  |
| **DAT_ Resting-** | *r_s_* | 0.030 | -0.040 | -0.080 | -0.070 | -0.010 | -0.100 | 0.010 |
| **State 2_Left** | *p* | 0.773 | 0.757 | 0.398 | 0.501 | 0.917 | 0.304 | 0.917 |
| N = 181 |  |  |  |  |  |  |  |  |
| **DAT_ Resting-** | *r_s_* | -0.190 * | 0.050 | 0.050 | 0.050 | 0.040 | 0.090 | -0.020 |
| **State 3_Left** | *p* | 0.029 | 0.665 | 0.665 | 0.665 | 0.690 | 0.356 | 0.809 |
| N = 156 |  |  |  |  |  |  |  |  |

To simplify, only names of neurotransmitters were listed to indicate the gradient-neurotransmitter similarity. The similarity between 5HT1A and the third-order gradient maps, D1 and the second-order gradient maps, DAT and the fourth-order gradient maps were included to correlate with mental health outcomes. The correction was applied across behavioral measures within each neurotransmitter and separately for each resting-state. Depressive severity was measured as the sum score of IDS-SR, and anxiety sensitivity was assessed with the ASI sum score. Childhood trauma and its subcategories were measured using the CTQ-SF. **p*<0.05.

**Table S18.** Spearman correlations between gradient-neurotransmitter similarity and mental health outcomes in MIND-Set (controlling for age, sex, medication)

|  |  | **Anxiety Sensitivity** | **Depressive Severity** | **Comorbidity** | **Childhood**  **Trauma** | **Emotional**  **Neglect** | **Psychological Abuse** | **Physical**  **Abuse** |
| --- | --- | --- | --- | --- | --- | --- | --- | --- |
| **5HT1A_ Resting-** | *r_s_* | -0.070 | 0.020 | 0.050 | -0.060 | -0.080 | -0.010 | -0.110 |
| **State 1_Left** | *p* | 0.332 | 0.801 | 0.506 | 0.386 | 0.279 | 0.833 | 0.148 |
| **5HT1A_ Resting-** | *r_s_* | 0.130 | 0.130 | 0.070 | -0.090 | -0.100 | -0.060 | -0.070 |
| **State 1_Right** | *p* | 0.071 | 0.071 | 0.332 | 0.229 | 0.171 | 0.398 | 0.332 |
| N = 234 |  |  |  |  |  |  |  |  |
| **5HT1A_ Resting-** | *r_s_* | 0.060 | -0.020 | 0.000 | 0.110 | 0.110 | 0.120 | 0.140 |
| **State 2_Left** | *p* | 0.488 | 0.794 | 0.984 | 0.203 | 0.199 | 0.170 | 0.079 |
| **5HT1A_ Resting-** | *r_s_* | -0.040 | 0.060 | -0.020 | 0.060 | 0.060 | 0.060 | 0.080 |
| **State 2_Right** | *p* | 0.668 | 0.445 | 0.824 | 0.445 | 0.463 | 0.487 | 0.354 |
| N = 203 |  |  |  |  |  |  |  |  |
| **5HT1A_ Resting-** | *r_s_* | -0.040 | -0.020 | 0.040 | 0.060 | 0.060 | 0.050 | -0.010 |
| **State 3_Left** | *p* | 0.712 | 0.840 | 0.728 | 0.515 | 0.515 | 0.563 | 0.881 |
| **5HT1A_ Resting-** | *r_s_* | 0.080 | 0.070 | -0.050 | -0.020 | -0.030 | -0.020 | -0.010 |
| **State 3_Right** | *p* | 0.367 | 0.462 | 0.641 | 0.840 | 0.751 | 0.840 | 0.881 |
| N = 234 |  |  |  |  |  |  |  |  |
| **D2_ Resting-** | *r_s_* | -0.030 | 0.010 | 0.030 | -0.020 | -0.020 | 0.010 | -0.100 |
| **State 1_Left** | *p* | 0.762 | 0.878 | 0.794 | 0.810 | 0.810 | 0.878 | 0.156 |
| N = 263 |  |  |  |  |  |  |  |  |
| **D2_ Resting-** | *r_s_* | -0.020 | -0.060 | 0.020 | 0.090 | 0.140 | 0.100 | 0.100 |
| **State 2_Left** | *p* | 0.773 | 0.359 | 0.773 | 0.167 | 0.035 | 0.128 | 0.128 |
| N = 257 |  |  |  |  |  |  |  |  |
| **D2_ Resting-** | *r_s_* | 0.000 | 0.050 | 0.020 | 0.090 | 0.080 | 0.080 | 0.060 |
| **State 3_Left** | *p* | 0.952 | 0.388 | 0.691 | 0.126 | 0.179 | 0.201 | 0.327 |
| N = 275 |  |  |  |  |  |  |  |  |
| **D1_ Resting-** | *r_s_* | 0.010 | 0.030 | -0.040 | 0.000 | -0.060 | 0.030 | 0.040 |
| **State 1_Left** | *p* | 0.885 | 0.701 | 0.655 | 0.944 | 0.410 | 0.681 | 0.558 |
| N = 276 |  |  |  |  |  |  |  |  |
| **D1_ Resting-** | *r_s_* | 0.130 | 0.090 | 0.060 | 0.030 | 0.050 | 0.050 | 0.020 |
| **State 2_Left** | *p* | 0.054 | 0.193 | 0.409 | 0.618 | 0.443 | 0.483 | 0.775 |
| N = 268 |  |  |  |  |  |  |  |  |
| **D1_ Resting-** | *r_s_* | 0.070 | 0.050 | -0.060 | 0.040 | 0.030 | 0.020 | -0.040 |
| **State 3_Left** | *p* | 0.323 | 0.522 | 0.404 | 0.545 | 0.656 | 0.802 | 0.545 |
| N = 275 |  |  |  |  |  |  |  |  |

The similarity between 5HT1A and the third-order gradient maps, D2 and the third-order gradient maps, D1 and the second-order gradient maps were included to correlate with mental health outcomes. The correction was applied across behavioral measures within each neurotransmitter and separately for each resting-state. Depressive severity was measured as the sum score of IDS-SR, and anxiety sensitivity was assessed with the ASI sum score. Childhood trauma and its subcategories were assessed using the NEMESIS. Comorbidity was indexed by the number of diagnosed psychiatric disorder clusters per participant.

**Table S19.** Spearman correlations between gradient-neurotransmitter similarity and mental health outcomes in MIND-Set, separately for patients and healthy controls

|  |  | **Anxiety Sensitivity** | **Depressive Severity** | **Comorbidity** | **Childhood**  **Trauma** | **Emotional**  **Neglect** | **Psychological Abuse** | **Physical**  **Abuse** |
| --- | --- | --- | --- | --- | --- | --- | --- | --- |
| **5HT1A_ Resting-** | *r_s_* | -0.080 | -0.010 | 0.030 | -0.090 | -0.100 | -0.020 | -0.130 |
| **State 1_Left_patient** | *p* | 0.363 | 0.862 | 0.803 | 0.332 | 0.280 | 0.803 | 0.117 |
| **5HT1A_ Resting-** | *r_s_* | 0.180 | 0.150 | 0.020 | -0.140 | -0.120 | -0.090 | -0.090 |
| **State 1_Right_patient** | *p* | 0.063 | 0.083 | 0.803 | 0.100 | 0.183 | 0.332 | 0.316 |
| N = 178 |  |  |  |  |  |  |  |  |
| **5HT1A_ Resting-** | *r_s_* | 0.130 | -0.040 | 0.070 | 0.110 | 0.090 | 0.090 | 0.140 |
| **State 2_Left_patient** | *p* | 0.181 | 0.709 | 0.504 | 0.256 | 0.346 | 0.350 | 0.130 |
| **5HT1A_ Resting-** | *r_s_* | -0.010 | 0.060 | 0.000 | 0.010 | -0.030 | 0.000 | 0.040 |
| **State 2_Right_patient** | *p* | 0.982 | 0.571 | 0.984 | 0.982 | 0.757 | 0.984 | 0.709 |
| N = 159 |  |  |  |  |  |  |  |  |
| **5HT1A_ Resting-** | *r_s_* | -0.050 | -0.060 | 0.020 | -0.030 | 0.020 | -0.010 | -0.110 |
| **State 3_Left_patient** | *p* | 0.643 | 0.586 | 0.828 | 0.826 | 0.826 | 0.910 | 0.238 |
| **5HT1A_ Resting-** | *r_s_* | 0.100 | 0.100 | -0.030 | -0.030 | -0.060 | 0.000 | 0.040 |
| **State 3_Right_patient** | *p* | 0.286 | 0.260 | 0.826 | 0.826 | 0.608 | 0.968 | 0.736 |
| N = 177 |  |  |  |  |  |  |  |  |
| **D2_ Resting-** | *r_s_* | -0.040 | -0.010 | 0.010 | -0.030 | -0.050 | 0.010 | -0.100 |
| **State 1_Left_patient** | *p* | 0.697 | 0.903 | 0.903 | 0.705 | 0.643 | 0.903 | 0.230 |
| N = 197 |  |  |  |  |  |  |  |  |
| **D2_ Resting-** | *r_s_* | 0.000 | -0.120 | 0.060 | 0.110 | 0.110 | 0.100 | 0.090 |
| **State 2_Left_patient** | *p* | 0.976 | 0.136 | 0.382 | 0.139 | 0.139 | 0.182 | 0.203 |
| N = 196 |  |  |  |  |  |  |  |  |
| **D2_ Resting-** | *r_s_* | -0.020 | 0.020 | -0.010 | 0.020 | 0.040 | 0.020 | 0.000 |
| **State 3_Left_patient** | *p* | 0.884 | 0.884 | 0.915 | 0.884 | 0.716 | 0.884 | 0.978 |
| N = 207 |  |  |  |  |  |  |  |  |
| **D1_ Resting-** | *r_s_* | 0.020 | 0.070 | -0.040 | 0.010 | -0.070 | 0.060 | 0.010 |
| **State 1_Left_patient** | *p* | 0.778 | 0.404 | 0.669 | 0.869 | 0.367 | 0.428 | 0.869 |
| N = 210 |  |  |  |  |  |  |  |  |
| **D1_ Resting-** | *r_s_* | 0.140 | 0.090 | 0.010 | 0.060 | 0.060 | 0.080 | 0.030 |
| **State 2_Left_patient** | *p* | 0.067 | 0.254 | 0.896 | 0.439 | 0.439 | 0.268 | 0.671 |
| N = 205 |  |  |  |  |  |  |  |  |
| **D1_ Resting-** | *r_s_* | 0.160 * | 0.130 | -0.110 | 0.080 | 0.090 | 0.080 | -0.020 |
| **State 3_Left_patient** | *p* | 0.027 | 0.079 | 0.148 | 0.288 | 0.239 | 0.260 | 0.807 |
| N = 208 |  |  |  |  |  |  |  |  |
| **5HT1A_ Resting-** | *r_s_* | -0.040 | 0.090 | 0.000 | 0.020 | 0.090 | 0.060 | 0.060 |
| **State1_Left_healthy control** | *p* | 0.934 | 0.891 | 1.000 | 0.960 | 0.891 | 0.891 | 0.891 |
| **5HT1A_ Resting-** | *r_s_* | -0.030 | -0.020 | 0.050 | 0.070 | 0.010 | 0.050 | 0.020 |
| **State1_Right_healthy control** | *p* | 0.949 | 0.960 | 0.892 | 0.891 | 0.961 | 0.892 | 0.960 |
| N = 59 |  |  |  |  |  |  |  |  |
| **5HT1A_ Resting-** | *r_s_* | -0.110 | 0.190 | 0.060 | -0.040 | 0.020 | 0.050 | -0.160 |
| **State2_Left_healthy control** | *p* | 0.745 | 0.535 | 0.839 | 0.840 | 0.897 | 0.840 | 0.535 |
| **5HT1A_ Resting-** | *r_s_* | -0.260 | 0.040 | -0.190 | 0.190 | 0.160 | 0.170 | 0.110 |
| **State2_Right_healthy control** | *p* | 0.343 | 0.840 | 0.535 | 0.535 | 0.535 | 0.535 | 0.745 |
| N = 48 |  |  |  |  |  |  |  |  |
| **5HT1A_ Resting-** | *r_s_* | -0.030 | 0.020 | 0.000 | 0.270 | 0.250 | 0.200 | 0.220 |
| **State3_Left_healthy control** | *p* | 0.912 | 0.935 | 1.000 | 0.188 | 0.220 | 0.310 | 0.310 |
| **5HT1A_ Resting-** | *r_s_* | 0.120 | 0.150 | -0.220 | 0.090 | 0.060 | 0.010 | -0.050 |
| **State3_Right_healthy control** | *p* | 0.579 | 0.432 | 0.310 | 0.732 | 0.854 | 0.963 | 0.871 |
| N = 59 |  |  |  |  |  |  |  |  |
| **D2_ Resting-** | *r_s_* | 0.070 | 0.120 | 0.020 | 0.110 | 0.270 | 0.170 | 0.080 |
| **State1_Left_healthy control** | *p* | 0.646 | 0.600 | 0.886 | 0.600 | 0.092 | 0.383 | 0.646 |
| N = 68 |  |  |  |  |  |  |  |  |
| **D2_ Resting-** | *r_s_* | -0.070 | 0.120 | -0.130 | -0.230 | 0.030 | -0.160 | -0.200 |
| **State2_Left_healthy control** | *p* | 0.674 | 0.662 | 0.662 | 0.232 | 0.821 | 0.565 | 0.335 |
| N = 65 |  |  |  |  |  |  |  |  |
| **D2_ Resting-** | *r_s_* | -0.020 | 0.010 | 0.160 | 0.190 | 0.150 | 0.170 | 0.250 |
| **State3_Left_healthy control** | *p* | 0.917 | 0.937 | 0.355 | 0.322 | 0.371 | 0.355 | 0.124 |
| N = 71 |  |  |  |  |  |  |  |  |
| **D1_ Resting-** | *r_s_* | -0.090 | 0.020 | -0.070 | -0.080 | 0.010 | -0.090 | 0.090 |
| **State1_Left_healthy control** | *p* | 0.638 | 0.929 | 0.638 | 0.638 | 0.963 | 0.638 | 0.638 |
| N = 69 |  |  |  |  |  |  |  |  |
| **D1_ Resting-** | *r_s_* | -0.050 | -0.110 | 0.000 | -0.140 | -0.060 | -0.180 | -0.140 |
| **State2_Left_healthy control** | *p* | 0.734 | 0.556 | 1.000 | 0.478 | 0.709 | 0.361 | 0.478 |
| N = 67 |  |  |  |  |  |  |  |  |
| **D1_ Resting-** | *r_s_* | -0.130 | -0.040 | -0.050 | 0.090 | 0.110 | -0.020 | 0.170 |
| **State3_Left_healthy control** | *p* | 0.523 | 0.784 | 0.727 | 0.641 | 0.589 | 0.873 | 0.385 |
| N = 70 |  |  |  |  |  |  |  |  |

The similarity between 5HT1A and the third-order gradient maps, D2 and the third-order gradient maps, D1 and the second-order gradient maps were included to correlate with mental health outcomes. The correction was applied across behavioral measures within each neurotransmitter and separately for each resting-state. Depressive severity was measured as the sum score of IDS-SR, and anxiety sensitivity was assessed with the ASI sum score. Childhood trauma and its subcategories were assessed using the NEMESIS. Comorbidity was indexed by the number of diagnosed psychiatric disorder clusters per participant. **p*<0.05.


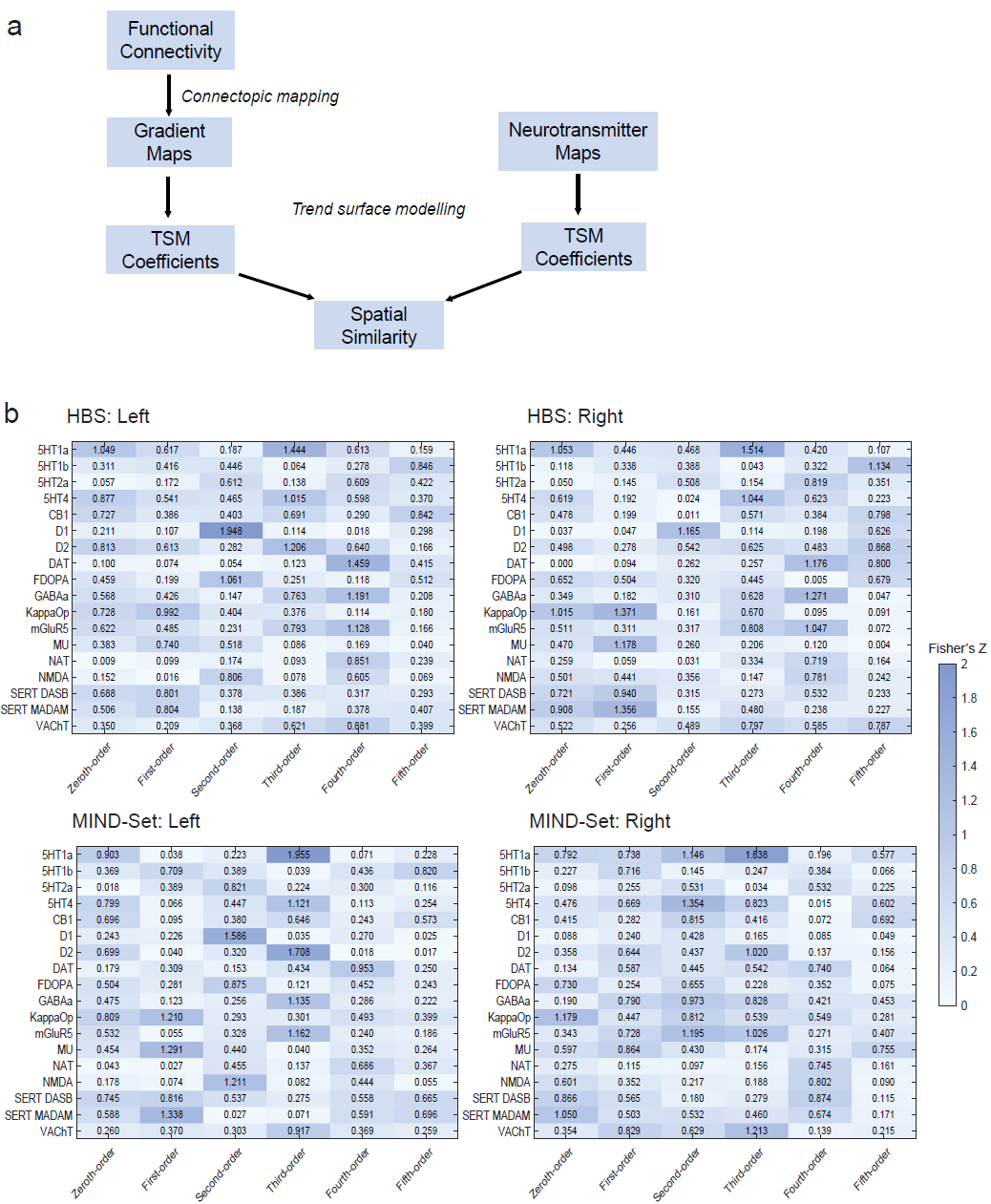


**Fig.S1 a** Schematic illustration of the pipeline used to estimate spatial similarities (correlation coefficients) between gradient maps and neurotransmitter maps. **b** Heat maps showing correlation coefficients (Fisher r-to-z transformed, absolute values) between gradient and neurotransmitter maps, with resting-state 1 shown as an example for each dataset


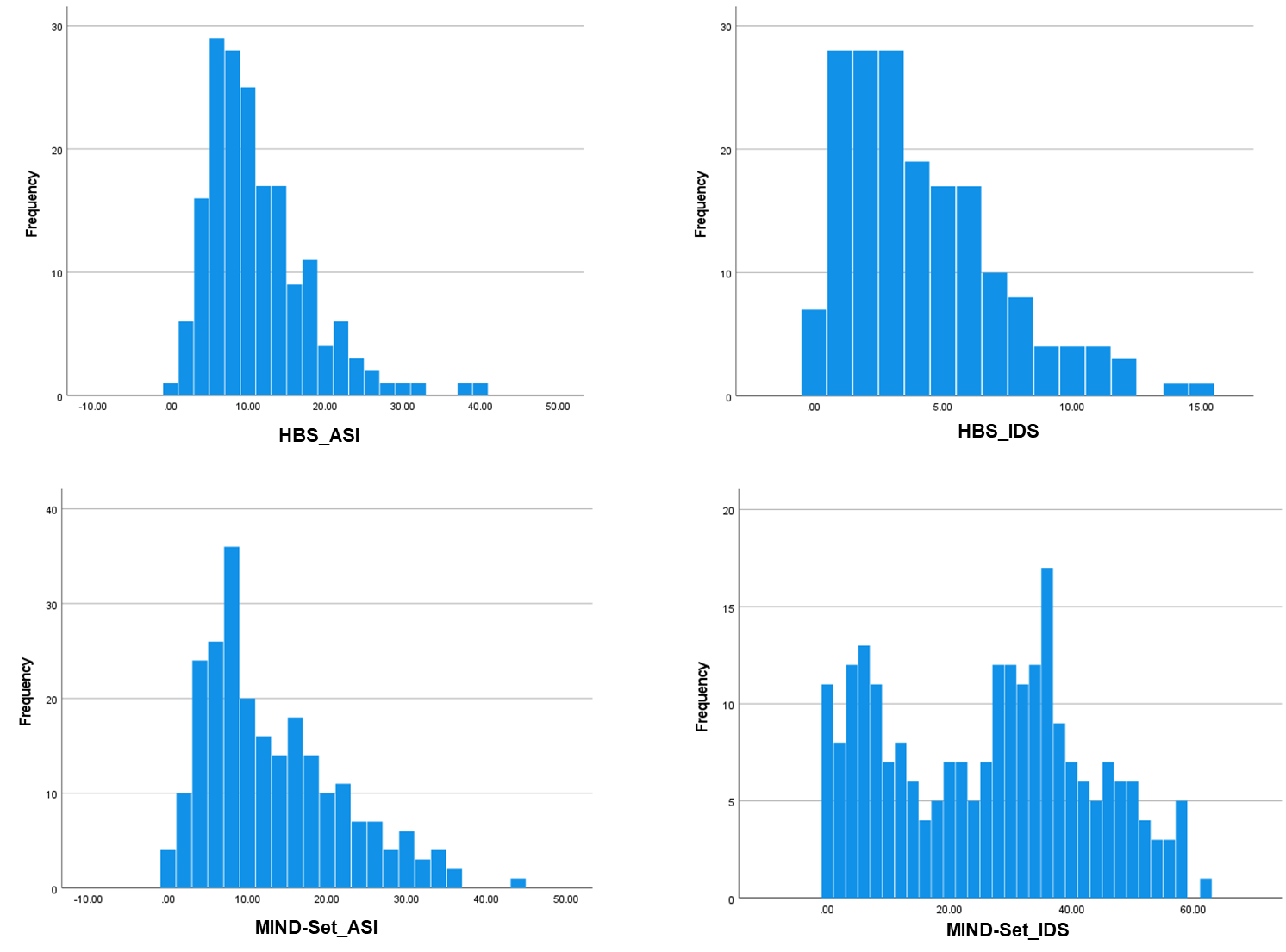


**Fig.S2** Distributions of ASI and IDS scores in the HBS (upper panel) and the MIND-Set sample (lower panel).

**References**

Alakurtti K, Johansson JJ, Joutsa J, et al (2015) Long-term test–retest reliability of striatal and extrastriatal dopamine D2/3 receptor binding: study with [11C] raclopride and high-resolution PET. J Cereb Blood Flow Metab 35:1199–1205

Beliveau V, Ganz M, Feng L, et al (2017) A high-resolution in vivo atlas of the human brain’s serotonin system. J Neurosci 37:120–128

DuBois JM, Rousset OG, Rowley J, et al (2016) Characterization of age/sex and the regional distribution of mglur5 availability in the healthy human brain measured by high-resolution [11 c] abp688 pet. Eur J Nucl Med Mol Imaging 43:152–162

Dukart J, Holiga Š, Chatham C, et al (2018) Cerebral blood flow predicts differential neurotransmitter activity. Sci Rep 8:4074

Fazio P, Schain M, Varnäs K, et al (2016) Mapping the distribution of serotonin transporter in the human brainstem with high-resolution PET: validation using postmortem autoradiography data. Neuroimage 133:313–320

Galovic M, Erlandsson K, Fryer TD, et al (2021) Validation of a combined image derived input function and venous sampling approach for the quantification of [18F] GE-179 PET binding in the brain. Neuroimage 237:118194

Gómez FJG, Huertas I, Ramírez JAL, Solís DG (2018) Elaboración de una plantilla de SPM para la normalización de imágenes de PET con 18F-DOPA. Imagen Diagnóstica 9:23–25

Hansen JY, Shafiei G, Markello RD, et al (2022) Mapping neurotransmitter systems to the structural and functional organization of the human neocortex. Nat Neurosci 25:1569–1581

Hesse S, Becker G-A, Rullmann M, et al (2017) Central noradrenaline transporter availability in highly obese, non-depressed individuals. Eur J Nucl Med Mol Imaging 44:1056–1064

Kaller S, Rullmann M, Patt M, et al (2017) Test–retest measurements of dopamine d 1-type receptors using simultaneous pet/mri imaging. Eur J Nucl Med Mol Imaging 44:1025–1032

Kantonen T, Karjalainen T, Isojärvi J, et al (2020) Interindividual variability and lateralization of μ-opioid receptors in the human brain. Neuroimage 217:116922

Laurikainen H, Tuominen L, Tikka M, et al (2019) Sex difference in brain CB1 receptor availability in man. Neuroimage 184:834–842

Oldehinkel M, Llera A, Faber M, et al (2022) Mapping dopaminergic projections in the human brain with resting-state fMRI. Elife 11:e71846

Savli M, Bauer A, Mitterhauser M, et al (2012) Normative database of the serotonergic system in healthy subjects using multi-tracer PET. Neuroimage 63:447–459

Shokri‐Kojori E, Naganawa M, Ramchandani VA, et al (2022) Brain opioid segments and striatal patterns of dopamine release induced by naloxone and morphine. Hum Brain Mapp 43:1419–1430
